# The Heterogeneity in Retrieved Relations between the Personality Trait ‘Harm Avoidance’ and Gray Matter Volumes Due to Variations in the VBM and ROI Labeling Processing Settings

**DOI:** 10.1371/journal.pone.0153865

**Published:** 2016-04-20

**Authors:** Peter Van Schuerbeek, Chris Baeken, Johan De Mey

**Affiliations:** 1 Departement of Radiology, UZ-Brussel, Vrije Universiteit (VUB), Brussels, Belgium; 2 Departement of Psychiatry, UZ-Brussel, Vrije Universiteit Brussel (VUB), Brussel, Belgium; 3 Departement of Psychiatry and Medical Psychology, Ghent University, Ghent, Belgium; Chinese Academy of Sciences, CHINA

## Abstract

Concerns are raising about the large variability in reported correlations between gray matter morphology and affective personality traits as ‘Harm Avoidance’ (HA). A recent review study (Mincic 2015) stipulated that this variability could come from methodological differences between studies. In order to achieve more robust results by standardizing the data processing procedure, as a first step, we repeatedly analyzed data from healthy females while changing the processing settings (voxel-based morphology (VBM) or region-of-interest (ROI) labeling, smoothing filter width, nuisance parameters included in the regression model, brain atlas and multiple comparisons correction method). The heterogeneity in the obtained results clearly illustrate the dependency of the study outcome to the opted analysis settings. Based on our results and the existing literature, we recommended the use of VBM over ROI labeling for whole brain analyses with a small or intermediate smoothing filter (5-8mm) and a model variable selection step included in the processing procedure. Additionally, it is recommended that ROI labeling should only be used in combination with a clear hypothesis and that authors are encouraged to report their results uncorrected for multiple comparisons as supplementary material to aid review studies.

## Introduction

The personality trait ‘Harm Avoidance’ (HA) from Cloninger’s psychobiological model of personality describes one’s tendency to inhibit actions and behaviors in anticipation to expected risks and personal harm [1–3]. In general, this temperamental dimension is closely related to one’s emotions of fear and anxiety and to similar affective personality traits as e.g. trait anxiety and neuroticism [4]. In healthy individuals, elevated HA scores were found to be indicative for an increased risk to develop a mood or anxiety disorder [5,6].

Given the hypothesized link between personality and one’s vulnerability for psychopathologies, a growing number of researchers tried to associate individual differences in brain morphology to differences in negative emotionality related personality traits (e.g. HA) [7]. Such an association could be of interest since it would indicate the existence of a neuroanatomical basis for affective traits. A recent review [7] revealed consistent reduced gray matter volumes (GMV) in the left medial orbitofrontal cortex (OFC) extending into the rostral anterior cingulate cortex (ACC) (Brodmann Area (BA) 32) and increased GMV in the left amygdala extending into the anterior parahippocampal gyrus in healthy individuals scoring high on negative emotionality traits. However, this review also showed a large heterogeneity in reported correlations.

To link brain morphology to personality, one can use a whole brain voxel-based morphology (VBM) approach. In VBM, each individual brain is first segmented into GMV and white matter volume (WMV) maps and normalized to a common template. Subsequently, a regression analysis is performed in each voxel from these tissue maps. The main advantage of VBM is its high sensitivity for small morphological variations within brain regions. However, a major drawback is that statistical tests are performed in up to 500,000 voxels. Consequently, VBM has an intrinsic high risk for false positive findings (type I errors) [8]. To limit this increased chance for type I errors, a region-of-interest (ROI) labeling approach can be used as an alternative method. In ROI labeling, each brain is parcellated into several anatomical regions according to an anatomical atlas. Subsequently, based on the hypothesis made, a regression or correlation analysis is performed on a limited number of these brain areas. Compared to VBM, ROI labeling has the advantage of a reduced risk for type I errors, but since tissue volumes are averaged over a larger brain area, this technique is only sensitive for more global morphological alterations. As such, VBM and ROI labeling are mostly considered as complementary techniques [9,10].

As stated by [7], the heterogeneity in reported relations between GMV and HA could partly be explained by methodological differences between studies. First of all, it is shown that the segmentation accuracy depends on used MRI machine and scanning sequence [11,12]. Secondly, several studies [13–15] revealed confounding effects of sample demographics (age and gender). Thirdly, since in ROI labeling more global regional GMV is related to HA, while in VBM, local GMV within the voxels is related to HA, one can hypothesize that the choice for one or the other technique could lead to various correlation results. Fourthly, [16,17] showed that, in general, the outcome in VBM studies is affected by the included nuisance covariates in the regression model and width of the used smoothing filter (necessary to satisfy the assumptions of Gaussian random field theory). Lastly, since the different packages nowadays used for automatic ROI labeling, differ in ROI segmentation algorithms and brain atlases [18], one can hypothesize that dissimilarities in the definition of a ROI between software packages can lead to differences in the outcome of the subsequent statistical tests. As illustrated in Table 1, the published studies [13,19–22] relating HA to GMV, differed in sample demographics, MRI scanner, used technique (VBM or ROI labeling), regression model and smoothing kernel size.

**Table 1:** Summary of studies relating GMV to HA.

Study	Materials and methods		Relations betwen GMV and HA
Van Schuerbeek et al. 2011 [22]	MRI scanner	1.5T Intera (Philips), 3T Achieva (Philips)	Left Superior Frontal Gyrus (+)
	Participants	76 (all females, age: 18–30)	Left Lingual Gyrus (+)
	Method	VBM	Left Inferior Frontal Gyrus (-)
	Preprocessing options	Smoothing FWHM = 8mm	Left Anterior Cingulate (-)
	Corrected variables	Age, Brain volume, NS, RD, P, SD, CO, ST	Left Cerebellar Tonsil (-)
	Multiple comparisons correction method	*p*unc<0.001 + K_e_>50	
			
Gardini et al. 2009 [21]	MRI scanner	3T Intera (Philips)	Right Cuneus (-)
	Participants	85 (58 males, 27 females, age: 32.69±6.48)	Right Inferior Parietal Lobule (-)
	Method	VBM	Left Precuneus (-)
	Preprocessing options	Smoothing FWHM = 12mm	Left Middle Occipital Gyrus (-)
	Corrected variables	Age, Years of education, Gender	Left Middle Frontal Gyrus (-)
	Multiple comparisons correction method	*p*FDR<0.05 + K_e_>0	Left and right Inferior Frontal Gyrus (-)
			
Yamasue et al. 2008 [13]	MRI scanner	1.5T Signa (GE)	Right Hippocamapus (-)
	Participants	183 (117 males, 66 females, age: 20–40)	
	Method	VBM	
	Preprocessing options	Smoothing FWHM = 12mm	
	Corrected variables	Brain volume	
	Multiple comparisons correction method	*p*FDR<0.05 + K_e_>0	
			
Iidaka et al. 2006 [20]	MRI scanner	3T Alegra (Siemens)	Left Amygdala (+)
	Participants	56 (30 males, 26 females, age: 22.3±3.1)	Left Orbital Gyrus (+)
	Method	VBM	Right Middle Temporal Gyrus (+)
	Preprocessing options	Smoothing FWHM = 12mm	Right Angular Gyrus (+)
	Corrected variables	Age, Gender, BDI	
	Multiple comparisons correction method	*p*unc<0.001 + K_e_>100	
			
Pujol et al. 2002 [19]	MRI scanner	1.5T Signa (GE)	Right Anterior Cingulate (+)
	Participants	100 (50 males, 50 females, age: 20–40)	
	Method	ROI labeling	
	Preprocessing options	Manual ROI labeling	
	Corrected variables	Brain volume	
	Multiple comparisons correction method	Bonferroni p<0.001	

VBM: voxel-based morphology, ROI: region-of-interest, *p*_unc_: uncorrected significance, *p*_FDR_: false discovery corrected significance, K_e_: cluster extend, NS: novelty seeking, RD: reward dependence, P: persistence, SD: Self-directedness, CO: cooperativeness, ST: self-transcendence. Reported positive relations were indicated by (+) and negative relations by (-).

Additionally, the VBM studies included in Table 1 [13,19–22] varied in the applied multiple comparisons correction method, to reduce the chance for a type I error. In general, regularly used correction methods in the neuroimaging literature are the family-wise error (FWE) correction, the false discovery rate (FDR) correction and the combination of an uncorrected voxel significance threshold (*p*_unc_) with a cluster size (K_e_) threshold. The FWE-correction method corrects the significance of a result (*p*_FWE_) for the number of voxels tested, to control the probability that only one of the voxels, surviving the corrected threshold, is a type I error. The FDR-correction method corrects the significance of a result (*p*_FDR_) for the rate of expected false positive findings. The combination of a *p*_unc_-threshold with a K_e-_threshold is inspired by the fact that the likelihood to find a significant statistical result in a whole cluster simply by chance, decreases when the size of a cluster increases [8]. Since none of the mentioned multiple comparisons correction methods discriminate real from false findings, but rather filter weak statistical results (FWE and FDR corrections) or small clusters (combined thresholds method), it can be hypothesized that the variety in used correction methods (Table 1) adds to the heterogeneity of the reported correlations between GMV and HA using VBM.

To study the heterogeneity and overlap in retrieved relations between GMV and HA due to variations in chosen processing settings, we repeatedly analyzed the same dataset with VBM and ROI labeling while changing the processing settings (smoothing filter, regression model and multiple comparisons correction method in VBM and brain atlas and regression model in ROI labeling). Our general hypothesized was that, even when processing the same data from a group of young healthy females gathered with the same scanner and sequence, differences in used analysis strategy can lead to very heterogeneous results.

## Materials and Methods

### Participants

To control for the confounding effects of age, gender and psychopathological vulnerability [13–15], we collected MRI data taken from 95 healthy females (age: 18–30 years). 25 datasets were taken from our previous VBM study [22] and 70 datasets were taken from fMRI studies going on at our department. The corresponding participants were all staff member or student at our hospital or at one of the participating universities: Vrije Universiteit Brussel (VUB) and Ghent University (UGent). All subjects were recruited by local advertising. For inclusion of the data in this study, the corresponding participant had to be medication free (except for birth-control medication), right-handed as assessed with the Van Strien questionnaire [23], free of any psychiatric disorder as assessed with the Dutch version of the Mini-International Neuropsychiatric Interview (Mini, [24]), without any personal psychiatric disorder history and being non depressed (defined as having a score lower than 9 on the 21 item Beck Depression Inventory [25]). All volunteers gave their written informed consent and were financially compensated. This study was approved by the Institutional Ethical Board of the University Hospital of the Vrije Universiteit Brussel (UZ Brussel, VUB) and in accordance with the guidelines laid down in the declaration of Helsinki [26].

### Imaging Protocol

For each participant, we collected the 3D T1-TFE anatomical scan data (TI/TR/ TE = 940.4/7.6/3.7 ms, flip angle = 8°, FOV = 240×240×200mm, resolution = 1×1×2mm and 100 axial slices) acquired at our 3T Achieva MRI system (Philips, Best, The Netherlands) with a quadrature transmit-receive head coil.

### TCI

All participants completed the Dutch version of the temperament and character inventory (TCI) [27] by answering “True” or “False” to 240 statements [3]. Based on the given answers, HA on a scale ranging from 0 to 40, novelty seeking (NS) on a scale from 0 to 40, reward dependence (RD) on a scale from 0 to 30, persistence (P) on a scale from 0 to 10, self-directedness (SD) on a scale from 0 to 50, cooperativeness (CO) on a scale from 0 to 50 and self-transcendence (ST) on a scale from 0 to 30, were determined.

### VBM Analyses

#### Preprocessing

All datasets were preprocessed in SPM8 (Statistical Parametric Mapping, Welcome Department of Imaging Neuroscience, London, UK) using the Diffeomorphic Anatomical Registration using Exponentiated Lie Algebra (DARTEL) normalization scheme [28]. First of all, all non-brain tissues were removed from the scans and the individual brains were segmented into GMV and WMV maps. Secondly, using DARTEL normalization, these individual maps were normalized to the MNI brain template space (Montreal Neurological Institute) in two steps. In the first step, a template was generated based on the tissue maps from all subjects and the deformation fields, to map the individual tissue maps to this template, were determined. In the second step, the generated template was normalized and the determined normalization parameters were used to normalize all individual GMV maps. During the normalization step, all tissue maps were resampled to an isotropic image resolution (1.5x1.5x1.5 mm³). The normalized tissue maps were modulated with the Jacobian determinant of the deformation field to correct for local expansions and contractions of the individual anatomy during the normalization stage and to end up with maps representing the GMV or WMV in each voxel rather than tissue densities. In this study, we limited the remaining of the VBM analyses to the GMV maps. The total gray matter volume (TGMV) for each individual was determined as the sum of all voxels from the GMV map.

#### The smoothing filter

To study the effect of changing the smoothing filter width on the study outcome, we produced three sets of smoothed GMV maps. The first set was smoothed with a small Gaussian filter (FWHM = 5mm). This filter was chosen since it was shown that such small filters are already sufficient to ensure the validity of the performed parametric tests [17]. The second set was smoothed with the SPM8 default Gaussian filter (FWHM = 8mm). The third set was smoothed with a large Gaussian filter (FWHM = 12mm). This latter filter was chosen since it was used by [13,20,21].

#### VBM sample homogeneity check

To be sure that our results were not affected by artifacts or segmentation and normalization errors, we inspected all data twice. First, all original scans and smoothed GMV maps were visually inspected for artifacts. Secondly, a sample homogeneity check using covariance check (from the VBM8 toolbox) was performed for each smoothed data set. In this test, for each GMV map, the squared distance to the sample mean was calculated. If this distance was larger than twice the sample standard deviation, both the GMV map and the original image were again visually inspected. After this check, all normalized GMV maps were found to be eligible for further analysis.

#### The regression analyses

On each smoothed data set, four regression analyses were performed, varying in regression model. The model used in the first analysis was chosen as our most basic model containing solely HA and TGMV as variables (model 1 (M1): HA, TGMV). Given the important age related changes in brain morphology [15], in the second analysis, age was added to M1 as a potential nuisance variable (model 2 (M2): HA, TGMV, Age). Since high HA in combination with low SD was found to be more sensitive as predictor for possible future affective psychopathologies than HA solely [29], SD was added to M2 in the third analysis (model 3 (M3): HA, TGMV, Age, SD). In the last analysis, all remaining personality traits were added to M3 (model 4 (M4): HA, TGMV, Age, NS, RD, P, SD, CO, ST), since all personality traits were found to correlate with brain morphology [22].

#### Multiple comparisons correction

To study the variability in filtering the results due to various multiple comparisons correction methods applied in the VBM analyses, for each cluster surviving *p*_unc_<0.001, the FWE and FDR-corrected significances for the peak voxel (the highest significance found within the cluster) and the probability that a similar cluster was found by chance (the cluster probability *p*_clus_) were determined. A result was considered as significant only if *p*_FWE_, *p*_FDR_ or *p*_clus_ was less than 0.05.

#### Comparison of the obtained VBM results

For each VBM analysis, a binary map was created, representing those voxels revealing a significant correlation between GMV and HA (1) or not (0). To study the overlap in the obtained results between analyses, the corresponding binary maps were added to each other and we searched for those voxels having a value equal to the number of maps added.

### ROI Labeling

#### Using BrainSuite

The first ROI labeling analysis was performed using the BrainSuite software tool [30] following all default cortical surface extraction and labeling stages (BrainSuite.org). In the first step, the brain was extracted from the image by removing all non-brain tissues. Secondly, to improve brain tissue classification, signal non-uniformities due to susceptibility differences at air-tissue boundaries and hardware imperfections were corrected. Subsequently, the brain was segmented into gray matter, white matter and cerebrospinal fluid (CSF). As a first step to parcellate the brain into its different anatomical areas, it was labeled into the left and right cerebrum, the ventricles, the cerebellum and the brainstem. From this labeled brain, a cerebrum mask was created which was used to limit all following processing steps to the cerebrum. To distinguish the cortex from the inner cortical area, the inner cerebellar cortex boundary was determined and an inner cortical mask was created. However, due to noise and image artifacts, segmentation errors occurred at the transition of gray matter to white matter, leading to bumps and holes at the inner cortex mask. To correct for this, the mask was scrubbed, smoothed by a graph-based topology correction and finally a wisp removal filter was applied. From the corrected cortical mask, a surface mesh of the inner cortex was created. Starting from this inner surface mesh and by using the segmented tissue fractions in each voxel, the inner surface was iteratively moved outwards, to determine the pial (outer cortical) surface. Finally, the individual brain was parcellated by an iterative registration of the pial and inner surfaces and the cerebrum volume mask to the BCI-DNI_brain atlas (brainsuite.org/svreg_atlas_description) subdivided into 95 ROIs (see the results table provided in S2 File for a full list of included anatomical labels). This latter step resulted in the labeled cerebrum volume and surface maps and in the determination of the GMV and TGMV for each ROI.

#### Using FreeSurfer

A second ROI labeling analysis was performed using FreeSurfer (http://surfer.nmr.mgh.harvard.edu). The automatic processing of each individual brain scan included the removal of non-brain tissues, a Talairach transformation, segmentation of the subcortical white matter and deep gray matter volumetric structures (aseg atlas [31]), intensity normalization, tessellation of the gray matter white matter boundary, a topology correction and a surface deformation step to end up with the reconstruction of the cortical surface and the volumetric segmentation into white matter, gray matter and cerebrospinal fluid. Subsequently, a surface inflation step and a registration to a spherical atlas step were performed. Finally, each cerebral cortex was parcellated into ROIs according to the cortical atlases provided by the FreeSurfer software (the Desikan-Killiany atlas [32], the Desikan-Killiany-Tourville (DKT 40) atlas [33] and some labels from the Talairach atlas). All 169 ROIs were used in the statistical analyses (see the results table provided in S3 File for a full list of anatomical labels included).

#### Using IBASPM

A last ROI labeling analysis was done using IBASPM [34]. In this analysis, we started from the VBM data from the VBM analyzes after segmention, DARTEL normalization and modulation steps. Although, IBASPM did not require smoothing, but since the provided help in the DARTEL normalization toolbox advices smoothing to correct aliasing during the modulation step, we selected the VBM dataset smoothed using the smallest smoothing kernel (FWHM = 5mm). The GMV maps were labeled according to the provided “Atlas 116 (116 brain structures)”. A list with the labeled structures is provided in S4 File. Per subject, the total GMV and the GMV per ROI were determined.

#### Statistical analyses

All statistical analyses were performed in SPSS separately for the ROIs obtained in BrainSuite and FreeSurfer.

Prior to the statistical analyses, all ROIs were checked for possible ROI labeling errors. First of all, all parcellated brains were inspected visually. Secondly, for each ROI a box plot was generated. The ROIs indicated as having an extreme high or low GMV were inspected more closely. Based on these checks, from the brains parcellated using the BrainSuite software, 3 subjects were excluded from further analyses due to severe whole brain ROI labeling errors. Local labeling errors were found in 16 subjects. No ROI labeling errors were found for the ROIs defined by FreeSurfer. The GMV for the ill labeled ROIs were replaced by a missing value. If no clear labeling error could be revealed, the outlier remained in the data.

Similar to the VBM analyses, 4 regression analyses using models M1, M2, M3 and M4 respectively, were performed per ROI. For each analysis, the obtained significances for HA as a predicting parameter for regional GMV, were Bonferroni and FDR corrected for the number of ROIs tested.

## Results

### Behavioral Results

The measured scores for HA ranged from 1 to 32 (mean = 15, SD = 7), for NS from 7 to 33 (mean = 22, SD = 6), for RD from 9 to 24 (mean = 19, SD = 3), for P from 0 to 8 (mean = 4, SD = 2), for SD from 9 to 42 (mean = 32, SD = 7), for CO from 24 to 42 (mean = 35, SD = 4) and for ST from 1 to 28 (mean = 9, SD = 6). These scores were similar to those reported for the females in [13].

Table 2 summarizes the mutual correlations between all personality traits and age. After Bonferroni correction (*p*<0.05), a negative correlation was found between HA and NS and between NS and P. A positive correlation was found between RD and CO. However, since all mutual correlations are rather weak and based on the review published in [35], we considered the personality traits and age as independent variables in our regression models.

**Table 2:** Summary of the mutual correlations between the personality traits and age.

	HA	NS	RD	P	SD	CO	ST
**HA**	---						
**NS**	-0.36 (<0.001)	---					
**RD**	0.11 (0.309)	0.04 (0.697)	---				
**P**	-0.07 (0.492)	-0.41 (<0.001)	0.06 (0.587)	---			
**SD**	-0.56 (<0.001)	0.09 (0.380)	-0.03 (0.774)	0.08 (0.464)	---		
**CO**	-0.10 (0.326)	-0.01 (0.954)	0.43 (<0.001)	0.08 (0.467)	0.16 (0.121)	---	
**ST**	0.01 (0.924)	0.10 (0.336)	-0.00 (0.984)	0.27 (0.010)	-0.26 (0.013)	0.24 (0.019)	---
**Age**	-0.12 (0.272)	0.04 (0.695)	0.23 (0.026)	0.18 (0.088)	0.31 (0.002)	0.16 (0.124)	-0.04 (0.726)

For each correlation we reported the correlation coefficient and the corresponding significance (between brackets) uncorrected for multiple comparisons.

### VBM Results

For review purposes, the results obtained without any multiple comparisons correction method applied, are given in S1 File. In the text, we only mentioned the results surviving the FWE (*p*_FWE_<0.05) or FDR (*p*_FDR_<0.05) correction or with a significant cluster probability (*p*_clus_<0.05).

#### Smoothing FWHM = 5mm

The analysis for M1 revealed a positive correlation between GMV and HA in the right precentral gyrus (K_e_ = 96, t = 5.41, *p*_FWE_ = 0.048, *p*_clus_ = 0.008) and a negative correlation in the right superior temporal pole (K_e_ = 101, t = 4.54, *p*_clus_ = 0.007) and in an area that was not labeled in the AAL or Talairach atlases (K_e_ = 58, t = 4.56, *p*_clus_ = 0.032). For M2, a positive correlation between GMV and HA was seen in the right precentral gyrus (K_e_ = 86, t = 5.27, *p*_clus_ = 0.011) and a negative correlation was seen in the left middle cingulate gyrus (K_e_ = 60, t = 4.12, *p*_clus_ = 0.029) and in the right superior temporal pole (K_e_ = 90, t = 4.41, *p*_clus_ = 0.010). Using M3, we obtained a positive correlation between GMV and HA in the left anterior cingulate cortex (K_e_ = 87, t = 4.55, *p*_clus_ = 0.011) and in the right precentral gyrus (K_e_ = 133, t = 4.73, *p*_clus_ = 0.002) and a negative correlation in the right precuneus (K_e_ = 73, t = 4.78, *p*_clus_ = 0.018), the right middle temporal pole (K_e_ = 75, t = 4.24, *p*_clus_ = 0.017) and the left inferior semi-lunar lobule (K_e_ = 188, t = 4.11, *p*_clus_ = 0.001). At last, using M4 a positive correlation was found in the right precentral gyrus (K_e_ = 56, t = 3.90, *p*_clus_ = 0.033), the left anterior cingulate gyrus (K_e_ = 65, t = 4.18, p_clus_ = 0.023) and the right precentral gyrus (K_e_ = 90, t = 3.83, *p*_clus_ = 0.009). A negative correlation was found in the right cuneus (K_e_ = 66, t = 4.89, *p*_clus_ = 0.022).

#### Smoothing FWHM = 8mm

The analysis using M1 revealed a positive correlation in the right inferior frontal gyrus (K_e_ = 197, t = 4.09, *p*_clus_ = 0.030) and in the right precentral gyrus (K_e_ = 346, t = 5.07, *p*_clus_ = 0.006). Using M2 a positive correlation between GMV and HA was found in the right inferior frontal gyrus (K_e_ = 199, t = 4.19, *p*_clus_ = 0.029) and in the right precentral gyrus (K_e_ = 319, t = 4.90, *p*_clus_ = 0.008). For M3, a positive correlation between GMV and HA was found in the left anterior cingulate gyrus (K_e_ = 178, t = 5.13, *p*_FWE_ = 0.049, *p*_FDR_ = 0.041, *p*_clus_ = 0.038) and in the right precentral gyrus (Ke = 346, t = 5.07, *p*_FDR_ = 0.041, *p*_clus_ = 0.005). A negative correlation was seen in an area not labeled by the AAL or Talairach atlas (K_e_ = 284, t = 4.66, *p*_clus_ = 0.011). At last, the analysis with M4 revealed a positive correlation between GMV and HA in a right sub-gyral area (K_e_ = 189, t = 4.94, *p*_clus_ = 0.032), and in two clusters in the right precentral gyrus (cluster 1: K_e_ = 200, t = 4.58, *p*_clus_ = 0.028; cluster 2: K_e_ = 279, t = 4.18, *p*_clus_ = 0.012).

#### Smoothing FWHM = 12mm

Even without any multiple comparisons correction applied, no correlations between GMV and HA were found for the analyses using M1 and M2. The correlations found using M3 and M4 did not survive any multiple comparisons correction.

### Overlap between the VBM Results

Since the analyses for the smoothing with filter FWHM = 12mm did not reveal any significant results, these analyses were omitted from the results comparisons.

#### Mutual comparisons of the results obtained using different regression models

**Fig 1:**
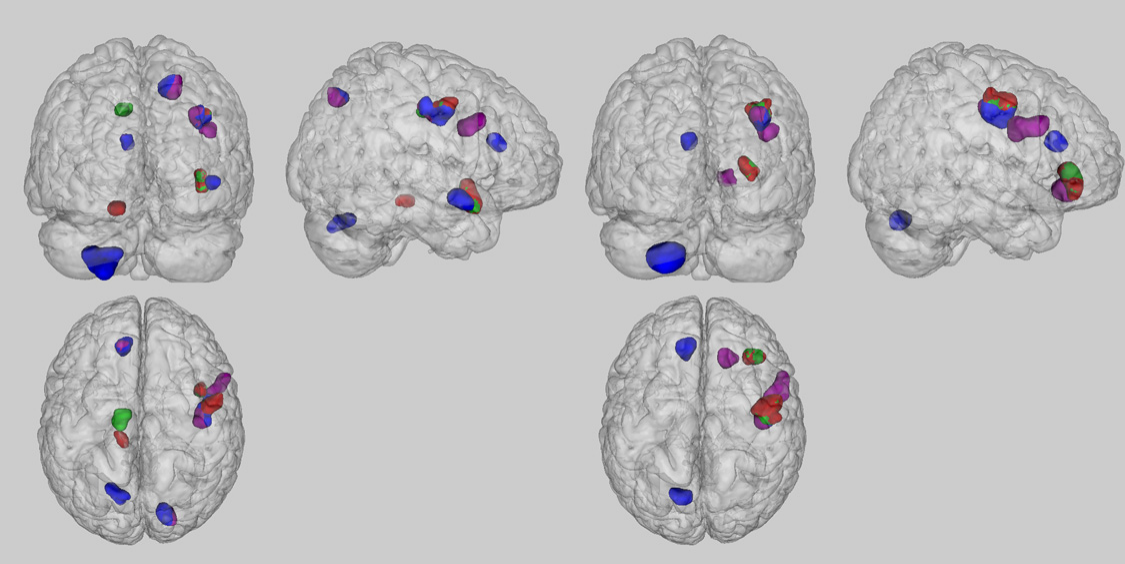
Superior, left and posterior view of the significant results found in the various analyses. The maps compare the results obtained from the smoothed FWHM = 5mm (left) and FWHM = 8mm (right) analyses using M1 (red), M2 (green), M3 (blue) and M4 (purple).

As presented in Table 3, The only cluster consistently found independent of used model, was situated in the right precentral gyrus. For the analyses performed on the smoothed FWHM = 5mm data, an additional overlap between the result maps of the analysis M1 and M2 was seen in the right superior temporal gyrus and between M3 and M4 in the left anterior cingulate cortex and right precuneus (Fig 1 left). For the analyses performed on the smoothed FWHM = 8mm data, an additional overlap between the results from analysis M1 and M2 was found in the right middle frontal gyrus (Fig 1 right).

**Table 3:** Results from the mutual comparisons of the obtained results from the analyses varying in used regression model.

		Number of voxels	Talairach	Anatomical labels	
Smoothing	Models		x,y,z {mm}	AAL atlas	TD atlas
FWHM = 5mm	M1+M2	88	[49.5; 9.0; -19.5]	Right Superior Temporal Pole	Right Superior Temporal Gyrus
		86	[39.0; -12.0; 31.5]	Not in atlas	Right Precentral Gyrus
	M1+M3	74	[39.0; -12.0; 31.5]	Not in atlas	Right Precentral Gyrus
	M1+M4	38	[39.0; -12.0; 31.5]	Not in atlas	Right Precentral Gyrus
	M2+M3	71	[39.0; -12.0; 31.5]	Not in atlas	Right Precentral Gyrus
	M2+M4	37	[39.0; -12.0; 31.5]	Not in atlas	Right Precentral Gyrus
	M3+M4	64	[-12.0; 36.0; 12.0]	Not in atlas	Left Anterior Cingulate Cortex
		46	[40.5; -12.0; 30.0]	Not in atlas	Right Precentral Gyrus
		15	[40.5; -18.0; 36.0]	Right Postcentral Gyrus	Right Precentral Gyrus
		51	[16.5; -75.0; 42.0]	Right Cuneus	Right Precuneus
	M1+M2+M3+M4	37	[39.0; -12.0; 31.5]	Not in atlas	Right Precentral Gyrus
FWHM = 8mm	M1+M2	191	[42.0; 31.5; -13.5]	Right Orbital Inferior Frontal Gyrus	Right Middle Frontal Gyrus
		317	[40.5; -10.5; 31.5]	Not in atlas	Right Precentral Gyrus
	M1+M3	225	[40.5; -10.5; 31.5]	Not in atlas	Right Precentral Gyrus
	M1+M4	159	[40.5; -10.5; 31.5]	Not in atlas	Right Precentral Gyrus
	M2+M3	152	[40.5; -10.5; 31.5]	Not in atlas	Right Precentral Gyrus
	M2+M4	212	[40.5; -10.5; 31.5]	Not in atlas	Right Precentral Gyrus
	M3+M4	240	[42.0; -10.5; 28.5]	Not in atlas	Right Precentral Gyrus
	M1+M2+M3+M4	144	[40.5; -10.5; 31.5]	Not in atlas	Right Precentral Gyrus

#### Comparison of the VBM results obtained using different smoothing filters

As presented in Table 4, the comparison of the VBM results obtained using the smoothing filters FWHM = 5mm and FWHM = 8mm revealed an overlap in the right precentral gyrus for all models. An additional overlap was found in the right anterior cingulate cortex for M3 and in the right inferior frontal gyrus for M4.

**Table 4:** Results from the pair wise comparisons of the result maps from the various analyses performed on the smoothed FWHM = 5mm and smoothed FWHM = 8mm datasets.

	Number of voxels	Talairach	Anatomical labels	
Models		x,y,z {mm}	AAL atlas	TD atlas
M1	88	[40.5; -10.5; 31.5]	Not in atlas	Right Precentral Gyrus
M2	80	[40.5; -10.5; 31.5]	Not in atlas	Right Precentral Gyrus
M3	117	[-19.5; -81.0; -46.5]	Not in atlas	Not in atlas
	81	[-12.0; 36.0; 12.0]	Not in atlas	Left Anterior Cingulate Cortex
	125	[42.0; -9.0; 28.5]	Not in atlas	Right Precentral Gyrus
M4	41	[51.0; 3.0; 22.5]	Right Precentral Gyrus	Right Inferior Frontal Gyrus
	87	[40.5; -12.0; 30.0]	Not in atlas	Right Precentral Gyrus

### ROI Labeling Results

The details of the results for the ROIs obtained in BrainSuite are given in S2 File. The details of the results from the ROI labeling analysis using FreeSurfer are provided in S3 File. However, none of the statistical tests revealed a significant relation between GMV and HA surviving Bonferroni or FDR correction for the number of ROIs tested.

For the ROIs from BrainSuite, interesting correlations between HA and GMV were found at trend level (significant at *p*_unc_<0.05) in the right fusiform gyrus (M1: t = 2.11, *p* = 0.038, M2: t = 2.01, *p* = 0.047, M4: t = 2.47, *p* = 0.016), left nucleus accumbens (M1: t = 2.50, *p* = 0.014, M2: t = 2.57, *p* = 0.012), left fusiform gyrus (M3: t = -2.23, *p* = 0.028, M4: t = -2.12, *p* = 0.037) right amygdale (M3: t = 2.85, *p* = 0.006, M4: t = 2.63, *p* = 0.010), left insula (M3: t = -2.03, *p* = 0.046), left globus pallidus (M3: t = -2.25, *p* = 0.027), right postcentral gyrus (M4: t = 2.14, *p* = 0.036), right parahippocampal gyrus (M4: t = -2.19, *p* = 0.031) and left putamen (M4: t = -1.99, *p* = 0.050).

The ROIs from the aseg atlas in FreeSurfer showed an interesting correlation at trend level in the right pallidum (M2: t = -2.05, *p* = 0.043, M3: t = -2.19, *p* = 0.031) and left thalamus proper (M4: t = -0.75, *p* = 0.033).

The ROIs from the Desikan-Killiany atlas showed a correlation at trend level in the left pars triangularis (M1: t = 3.11, *p* = 0.002), left rostral anterior cingulate (M1: t = -2.27, *p* = 0.026, M2: t = -2.10, *p* = 0.039, M3: t = -2.61, *p* = 0.011), left rostral middle frontal gyrus (M1: t = 2.00, p = 0.049, M3: t = 2.72, *p* = 0.008, M4: t = 2.75, *p* = 0.007), right pars orbitalis (M1: t = -3.27, *p* = 0.002, M2: t = -3.56, *p* = 0.001, M3: t = -2.80, *p* = 0.006), right precentral gyrus (M1: t = -2.31, *p* = 0.023, M3: t = -2.38, *p* = 0.019), right entorhinal gyrus (M3: t = -2.17, *p* = 0.032), right rostral middle frontal gyrus (M3: t = 2.00, *p* = 0.049), right inferior parietal gyrus (M4: t = 2.18, *p* = 0.032) and right pars triangularis (M4: t = 2.23, *p* = 0.029).

The ROIs from the DKT 40 atlas revealed correlations at trend level in the left paracentral gyrus (M1: t = -2.27, *p* = 0.026, M2: t = -2.33, *p* = 0.022), left pars triangularis (M1: t = 2.72, *p* = 0.008, M2: t = 2.50, *p* = 0.014), left rostral middle frontal gyrus (M1: t = 2.59, *p* = 0.011, M2: t = 2.50, *p* = 0.014, M3: t = 2.52, *p* = 0.013, M4: 2.61, *p* = 0.012), right pars orbitalis (M1: t = -3.27, *p* = 0.002, M2: t = -3.55, *p* = 0.001, M3: t = -2.94, *p* = 0.004), right precentral gyrus (M1: t = -2.28, *p* = 0.025, M2: t = -2.40, *p* = 0.018, M3: t = -2.40, *p* = 0.018), right rostral middle frontal gyrus (M1: t = 2.89, *p* = 0.005, M2: t = 2.86, *p* = 0.005, M3: t = 2.57, *p* = 0.012, M4: t = 2.77, *p* = 0.007), right entorhinal gyrus (M3: t = -2.66, *p* = 0.009), right inferior parietal gyrus (M4: t = 2.11, *p* = 0.038) and right pars triangularis (M4: t = 2.38, p = 0.019)

The ROIs from the Talairach atlas revealed a correlation between GMV and HA at trend level in the left Brodmann area (BA) 45 (M1: t = 3.22, *p* = 0.002, M2: t = 3.03, *p* = 0.003, M4: t = 2.31, *p* = 0.023), right BA 4a (M2: t = -1.99, *p* = 0.049, M3: t = -2.11, *p* = 0.038), right BA 6 (M2: t = -2.03, p = 0.046), right perirhinal cortex (M3: t = -2.00, *p* = 0.049) and right BA 45 (M4: t = 2.10, *p* = 0.039).

The ROIs defined by IBASPM, revealed solely a correlation between GMV and HA at trend level in the right Heschl gyrus using M1 (t = 2.19, *p* = 0.031) and M4 (t = 2.02, *p* = 0.046). However, these correlations did not survive FDR or FWE corrections.

## Discussion

In the current study, we compared several brain morphology analysis approaches to relate individual differences in GMV to the personality trait HA. More specifically, we repeatedly analyzed the same data from healthy young females, using VBM and ROI labeling with various processing settings. The aim of this study was threefold. First of all, we aimed to illustrate that the subtle correlations found in small clusters using VBM, are hardly retrievable when using a ROI approach due to the averaging of GMV over larger brain areas. Secondly, given the various processing settings used in published studies and since most researchers do not argue the chosen settings, we intended to illustrate the non-negligible effects these settings have on the study outcome. At last, given that it is used to report solely those results surviving a multiple comparisons correction while omitting those that did not survive such a correction, despite the already mentioned theoretical concerns [8], we intended to illustrate in practice the various filtering effects of the most often used multiple comparisons correction methods in the VBM literature. With this example, we intended to stimulate a more thought, standardized and argued use and reporting for these settings. Additionally, we intended to stimulate researchers to make their multiple comparisons uncorrected results available to the scientific world in addition to the published corrected results, to aid latter review studies.

The performed VBM analyses revealed heterogeneous results when smoothing was done with a small (FWHM = 5mm) or intermediate (FWHM = 8mm) filter, while the VBM analyses done using a large smoothing filter (FWHM = 12mm) and the ROI labeling analyses revealed only negative results.

When comparing the outcome from the VBM analyses with those from the ROI labeling analyses, our results seems to suggest that VBM is more sensitive to detect possible small local correlations between GMV and HA. The lack of any overlap between the results from our VBM and ROI labeling analyses is contrary to the results from previous comparison studies showing that both techniques are comparable sensitive in detecting hippocampal atrophy in patients compared to healthy controls [9,10,36]. Compared to these comparison studies, in the current study, we tried to explain the observed variation in local morphology within a healthy subject group. The variation within a healthy subjects group is evidently smaller than the reduction in GMV observed in patients, given the consistent significant differences found between both groups. Moreover, the similarity in obtained results using VBM and ROI labeling in clinical groups, indicate that disease states affect the regional morphology globally. On the other hand, the small cluster sizes found in our VBM studies and the lack of any significant correlation found in our ROI labeling studies indicate that personality affect brain morphology rather locally. Our results seem to indicate that the local relations between GMV and HA found using VBM, are hardly retrievable using ROI labeling due to the averaging of GMV over larger brain areas [9,10]. However, since VBM analyses are more sensitive for false positive findings and given the heterogeneity in the obtained VBM results, one can also state that the negative findings found in the ROI labeling analyses, are actually the true results.

In this study we also illustrated the various filtering effects of the most common applied multiple comparisons correction methods. As argued by [8], the Bonferroni and FDR correction were found to be very strict and resulted in deleting all results that were significant at an uncorrected threshold in most of the VBM analyses and in all ROI analyses. Applying a multiple comparisons correction based on the cluster size was found to be less stringent and resulted in most VBM analyses in some clusters that survived the applied correction. Additionally, since both the Bonferroni correction and the FDR correction become more stringent by an increasing number of ROIs tested, one can speculate that some of the trends found, could have survived a multiple comparisons correction, if we would have limited the ROI labeling analyses to only a limited number of ROIs in the frontal and limbic cortex based on a clear hypothesis. Given this, it seems advisable to use ROI labeling only in combination with a specific hypothesis. To perform whole brain analyses, VBM seems to be a better approach.

Although, we do not underestimate the importance of reducing the risk for false positive findings, we recommend that authors report their uncorrected results as supplementary material in addition to the multiple comparisons corrected results in the body of the manuscript, to take care of the risk for false negative findings and to aid latter review studies. This recommendation is inspired by the fact that none of the applied correction methods do discriminate real from false positive findings but only filter weak results. Since all correlation coefficients reported in the literature for the fitting of an explanatory model, including personality traits, to GMV ranged from 0.4 to 0.6 [13,19–22], it is hard to have strong statistical results even for very large sample sizes [37–39].

Our VBM analyses varying in used smoothing filter, revealed small clusters in the FWHM = 5mm analyses which disappeared when increasing the smoothing filter width. This tendency is in line with the interpretation that possible correlations between GMV and HA are more local and that smoothing the GMV maps using larger filters, results in averaging out these local morphological variations. Moreover, the simulations done by [17] revealed that VBM studies generally benefit from smaller filter widths. Important to note, the FWHM = 8mm analyses revealed significant correlations in the frontal cortex which were not found in the FWHM = 5mm analyses (Fig 1). A possible explanation for these observations is that smoothing the GMV maps reduced the noise in the maps and consequently, increased the sensitivity of the analyses. However, one can also state that the applied larger smoothing filter is able to induce accidental correlations between GMV and HA. Based on [17] and our own findings, we advise to use a smoothing filter width between 5mm and 8mm.

The comparison of the VBM results obtained using different regression models, showed a model independent relation between GMV and HA in the right precentral gyrus. Additionally, each analysis revealed correlations between GMV and HA that could not be found with any of the other models. Compared to [7], none of our VBM analyses succeeded in revealing a negative correlation in the left medial frontal gyrus or a positive correlation in the left amygdale. Noteworthy, despite the reuse of data from a previous study [22], none of the correlations reported in that paper, were retrieved by any of the analyses performed in the current study. One explanation could be that in [22] data from different scanners and scanned with different imaging sequences were combined while for the current study, only data from one scanner and scanned with the same sequence were included. It is shown that the chosen imaging protocol and MRI scanner can both affect the VBM outcome [11,12].

The variability in the obtained results depending on the used regression model, shows that an inconsistent inclusion of nuisance parameters in a VBM study adds to the heterogeneity of the reported correlations in the existing literature. However, given that so many factors besides age and personality (psychopathological vulnerability (BDI), education, training, alcohol use, medication, …) are known to affect brain morphology, it is impossible to control for all possible confounding factors given that including too many redundant variables in a regression model would drop the power of the analysis. Moreover, most of these factors do affect local brain morphology differently in different brain regions while they do not necessarily affect the morphology in all brain regions. To relate differences in HA to variations in local GMV, while optimally controlling for the most relevant confounding factors, it would be advisable to include, per brain area, a model parameter selection step in the VBM procedure [16]. To determine the best regression model, structural equation modeling techniques such as Akaike Information Criterion (AIC) or Bayesian Information Criterion (BIC) can be used [16]. Such a model selection criterion searches for the “best” model that explains the variability in local GMV by factors as for instance HA, while controlling the number of redundant parameters. Moreover, including a model selection criterion has the additional advantage that interesting mutual interactions between these factors in the brain, can be studied.

Although, none of the ROI analyses succeeded in revealing results surviving any multiple comparisons correction, the interesting trends found varied depending on the anatomical atlas used. In general, FreeSurfer seems to give more robust ROI labeling and fitting results than BrainSuite or IBASPM. Interestingly, almost all regression analyses performed on the ROIs from FreeSurfer, revealed a negative correlation between GMV and HA at trend level in the rostral anterior cingulated gyrus, which is in accordance with the review paper of [7].

In the current study we used real data rather than simulated data, since the used simulation method could have benefited one or the other analysis. Moreover, a review study as the one performed by [7] also starts from the assumption that methodological differences between studies do not prevent these studies to repeat a real relation between GMV and HA. The main drawback of using real data is that we do not know the true result. Consequently, we cannot conclude which analysis resulted in the most reliable results and which analyses resulted in mainly false positive or false negative findings. However, even without knowing the true result, the variability in obtained findings still illustrate the major impact of the chosen processing settings on the final study outcome

## Conclusion

The heterogeneity in obtained relations between GMV and HA in the repeated analyses performed in this study, illustrate the impact of the chosen morphology processing technique (VBM or ROI labeling) and the opted settings for the smoothing filter, regression model, anatomical atlas and the applied multiple comparisons correction on the study outcome. In general, our study seems to recommend the use of VBM over ROI labeling for whole brain analyses with small or intermediate smoothing filters (5mm≤FWHM≤8mm) and a model variable selection strategy per brain area included in the processing procedure. Additionally, it is recommended to use ROI labeling only to test a clear hypothesis and that authors should be encouraged to report their results uncorrected for multiple comparisons as supplementary material. These recommendations are in line with the recommendation suggested by [7,16,17,40].

## Supporting Information

S1 File Uncorrected results from the performed VBM analyses. Those results that remained significant after multiple comparisons correction were indicated.
(PDF)

S2 File Results from the performed ROI analyses using BrainSuite. None of the correlations survived multiple comparisons correction.
(PDF)

S3 File Results from the performed ROI analyses using FreeSurfer. None of the correlations survived multiple comparisons correction.
(PDF)

S4 File Results from the performed ROI analyses using IBASPM. None of the correlations survived multiple comparisons correction.
(PDF)
