# Supplementary material for: The Heterogeneity in Retrieved Relations between the Personality Trait ‘Harm Avoidance’ and Gray Matter Volumes Due to Variations in the VBM and ROI Labeling Processing Settings
Source: PLoS One. 2016 Apr 20;11(4):e0153865. doi: 10.1371/journal.pone.0153865 (PMC4838261; doi:10.1371/journal.pone.0153865)
Supplement: S1 File — Those results that remained significant after multiple comparisons correction were indicated. (PDF) [file pone.0153865.s001.pdf]

| Positive correlations found between GMV and HA | | | | | | | | |
|---|---|---|---|---|---|---|---|---|
| **Model = M1** | | | | | | | | |
| **Smoothing filter: FWHM=5mm** | | | | | | | | |
| | **peak** | **peak** | **peak** | **peak** | **cluster** | **Talairach** | **Anatomical labels** | |
| **Ke** | **T** | ***p* unc** | ***p* FWE** | ***p* FDR** | ***p* clus** | **x,y,z {mm}** | **AAL atlas** | **TD atlas** |
| 1 | 3,40 | 0,001 | 1,000 | 0,991 | 0,794 | [-7,5; -7,5; 76,5] | Not in atlas | Left Superior Frontal Gyrus |
| 1 | 3,34 | 0,001 | 1,000 | 0,991 | 0,794 | [4,5; -6,0; 75,0] | Not in atlas | Right Superior Frontal Gyrus |
| 1 | 3,33 | 0,001 | 1,000 | 0,991 | 0,794 | [-34,5; -24,0; -3,0] | Not in atlas | Left Extra Nuclear |
| 1 | 3,31 | 0,001 | 1,000 | 0,991 | 0,794 | [-42,0; -19,5; -9,0] | Not in atlas | Left Sub-Gyral |
| 1 | 3,26 | 0,001 | 1,000 | 0,991 | 0,794 | [-48,0; 36,0; 13,5] | Left Triangular Inferior Frontal Gyrus | Left Inferior Frontal Gyrus |
| 1 | 3,23 | 0,001 | 1,000 | 0,991 | 0,794 | [-37,5; -25,5; -3,0] | Not in atlas | Left Extra Nuclear |
| 1 | 3,23 | 0,001 | 1,000 | 0,991 | 0,794 | [6,0; -18,0; 55,5] | Right Supplementary Motor Area | Right Medial Frontal Gyrus |
| 1 | 3,23 | 0,001 | 1,000 | 0,991 | 0,794 | [-34,5; -51,0; 48,0] | Left Inferior Parietal Gyrus | Left Inferior Parietal Lobule |
| 1 | 3,23 | 0,001 | 1,000 | 0,991 | 0,794 | [-42,0; -25,5; 0,0] | Not in atlas | Left Sub-Gyral |
| 1 | 3,22 | 0,001 | 1,000 | 0,991 | 0,794 | [10,5; 9,0; 63,0] | Right Supplementary Motor Area | Right Superior Frontal Gyrus |
| 1 | 3,21 | 0,001 | 1,000 | 0,991 | 0,794 | [-9,0; -6,0; 13,5] | Not in atlas | Left Thalamus |
| 1 | 3,21 | 0,001 | 1,000 | 0,991 | 0,794 | [40,5; -21,0; 6,0] | Right Heshl Gyrus | Right Insula |
| 1 | 3,20 | 0,001 | 1,000 | 0,991 | 0,794 | [39,0; -67,5; 3,0] | Right Middle Occipital Gyrus | Right Middle Occipital Gyrus |
| 1 | 3,19 | 0,001 | 1,000 | 0,991 | 0,794 | [-22,5; -33,0; 57,0] | Left Postcentral Gyrus | Left Postcentral Gyrus |
| 1 | 3,19 | 0,001 | 1,000 | 0,991 | 0,794 | [12,0; 9,0; 1,5] | Right Caudate | Right Caudate |
| 1 | 3,19 | 0,001 | 1,000 | 0,991 | 0,794 | [48,0; -6,0; -24,0] | Right Middle Temporal Gyrus | Right Fusiform Gyrus |
| 2 | 3,37 | 0,001 | 1,000 | 0,991 | 0,694 | [54,0; -3,0; -4,5] | Right Superior Temporal Gyrus | Right Superior Temporal Gyrus |
| 2 | 3,31 | 0,001 | 1,000 | 0,991 | 0,694 | [37,5; -10,5; 9,0] | Right Insula | Right Insula |
| 3 | 3,43 | 0,000 | 1,000 | 0,991 | 0,620 | [30,0; 34,5; 45,0] | Right Middle Frontal Gyrus | Right Middle Frontal Gyrus |
| 3 | 3,35 | 0,001 | 1,000 | 0,991 | 0,620 | [-6,0; -3,0; 42,0] | Left Middle Cingulum | Left Cingulate Gyrus |
| 3 | 3,35 | 0,001 | 1,000 | 0,991 | 0,620 | [-54,0; -70,5; 16,5] | Not in atlas | Left Middle Temporal Gyrus |
| 3 | 3,31 | 0,001 | 1,000 | 0,991 | 0,620 | [9,0; -6,0; 15,0] | Not in atlas | Lateral Ventricle |
| 3 | 3,31 | 0,001 | 1,000 | 0,991 | 0,620 | [45,0; -3,0; -22,5] | Right Middle Temporal Gyrus | Right Sub-Gyral |
| 3 | 3,27 | 0,001 | 1,000 | 0,991 | 0,620 | [-19,5; -43,5; 58,5] | Left Superior Parietal Gyrus | Left Sub-Gyral |
| 4 | 3,45 | 0,000 | 1,000 | 0,991 | 0,560 | [-6,0; -13,5; 48,0] | Left Supplementary Motor Area | Left Medial Frontal Gyrus |
| 4 | 3,29 | 0,001 | 1,000 | 0,991 | 0,560 | [4,5; -9,0; 13,5] | Not in atlas | Right Thalamus |
| 4 | 3,28 | 0,001 | 1,000 | 0,991 | 0,560 | [-40,5; -12,0; 54,0] | Left Precentral Gyrus | Left Precentral Gyrus |
| 6 | 3,75 | 0,000 | 1,000 | 0,991 | 0,468 | [-66,0; -33,0; -24,0] | Not in atlas | Not in atlas |
| 6 | 3,48 | 0,000 | 1,000 | 0,991 | 0,468 | [42,0; -16,5; 39,0] | Right Precentral Gyrus | Right Precentral Gyrus |
| 6 | 3,46 | 0,000 | 1,000 | 0,991 | 0,468 | [-37,5; -6,0; 55,5] | Left Precentral Gyrus | Left Precentral Gyrus |
| 7 | 3,65 | 0,000 | 1,000 | 0,991 | 0,431 | [55,5; 12,0; 30,0] | Right Precentral Gyrus | Right Inferior Frontal Gyrus |
| 7 | 3,47 | 0,000 | 1,000 | 0,991 | 0,431 | [36,0; 19,5; 9,0] | Right Insula | Right Insula |
| 7 | 3,34 | 0,001 | 1,000 | 0,991 | 0,431 | [-13,5; -48,0; 37,5] | Left Middle Cingulum | Left Precuneus |
| 9 | 4,01 | 0,000 | 0,994 | 0,991 | 0,370 | [6,0; -66,0; 51,0] | Right Precuneus | Right Precuneus |
| 11 | 3,89 | 0,000 | 0,999 | 0,991 | 0,321 | [-13,5; 0,0; 67,5] | Left Superior Frontal Gyrus | Left Superior Frontal Gyrus |
| 11 | 3,77 | 0,000 | 1,000 | 0,991 | 0,321 | [-46,5; -43,5; 52,5] | Left Inferior Parietal Gyrus | Left Inferior Parietal Lobule |
| 11 | 3,56 | 0,000 | 1,000 | 0,991 | 0,321 | [-12,0; 49,5; 27,0] | Left Superior Frontal Gyrus | Left Superior Frontal Gyrus |
| 11 | 3,47 | 0,000 | 1,000 | 0,991 | 0,321 | [7,5; -25,5; 34,5] | Right Middle Cingulum | Right Cingulate Gyrus |
| 12 | 3,59 | 0,000 | 1,000 | 0,991 | 0,299 | [36,0; 30,0; 0,0] | Right Triangular Inferior Frontal Gyrus | Right Inferior Frontal Gyrus |
| 16 | 3,76 | 0,000 | 1,000 | 0,991 | 0,232 | [7,5; 21,0; -15,0] | Right Rectus | Right Subcallosal Gyrus |
| 20 | 3,62 | 0,000 | 1,000 | 0,991 | 0,184 | [-48,0; -55,5; -31,5] | Left Crus 1 Cerebelum | Left Cerebellar Tonsil |
| 23 | 4,10 | 0,000 | 0,980 | 0,991 | 0,156 | [48,0; -13,5; 45,0] | Right Precentral Gyrus | Right Precentral Gyrus |
| 29 | 4,09 | 0,000 | 0,982 | 0,991 | 0,114 | [55,5; -16,5; 21,0] | Right Rolandic Operculum | Right Postcentral Gyrus |
| 34 | 4,45 | 0,000 | 0,732 | 0,738 | 0,089 | [21,0; 60,0; 4,5] | Right Superior Frontal Gyrus | Right Superior Frontal Gyrus |
| 38 | 3,78 | 0,000 | 1,000 | 0,991 | 0,074 | [-12,0; 34,5; 16,5] | Left Anterior Cingulum | Left Anterior Cingulate Gyrus |
| 43 | 3,88 | 0,000 | 0,999 | 0,991 | 0,059 | [-28,5; 45,0; 28,5] | Left Middle Frontal Gyrus | Left Superior Frontal Gyrus |
| 96 | 5,41 | 0,000 | 0,048 | 0,055 | 0,008 | [45,0; -7,5; 36,0] | Right Precentral Gyrus | Right Precentral Gyrus |

| Negative correlations found between GMV and HA | | | | | | | | |
|---|---|---|---|---|---|---|---|---|
| **Model = M1** | | | | | | | | |
| **Smoothing filter: FWHM=5mm** | | | | | | | | |
| | **peak** | **peak** | **peak** | **peak** | **cluster** | **Talairach** | **Anatomical labels** | |
| **Ke** | **T** | ***p* unc** | ***p* FWE** | ***p* FDR** | ***p* clus** | **x,y,z {mm}** | **AAL atlas** | **TD atlas** |
| 1 | 3,23 | 0,001 | 1,000 | 0,991 | 0,794 | [3,0; 34,5; 54,0] | Right Medial Superior Frontal Gyrus | Not in atlas |
| 1 | 3,22 | 0,001 | 1,000 | 0,991 | 0,794 | [15,0; -25,5; 39,0] | Not in atlas | Right Cingulate Gyrus |
| 1 | 3,22 | 0,001 | 1,000 | 0,991 | 0,794 | [9,0; 51,0; -3,0] | Right Medial Superior Frontal Gyrus | Right Medial Frontal Gyrus |
| 1 | 3,20 | 0,001 | 1,000 | 0,991 | 0,794 | [16,5; -36,0; -24,0] | Not in atlas | Not in atlas |
| 1 | 3,19 | 0,001 | 1,000 | 0,991 | 0,794 | [7,5; -43,5; -37,5] | Not in atlas | Not in atlas |
| 1 | 3,19 | 0,001 | 1,000 | 0,991 | 0,794 | [57,0; -57,0; -4,5] | Right Inferior Temporal Gyrus | Right Inferior Temporal Gyrus |
| 2 | 3,47 | 0,000 | 1,000 | 0,779 | 0,694 | [22,5; -24,0; -10,5] | Not in atlas | Right Parahippocampal Gyrus |
| 3 | 3,61 | 0,000 | 1,000 | 0,779 | 0,620 | [-3,0; -72,0; 37,5] | Left Cuneus | Left Precuneus |
| 3 | 3,52 | 0,000 | 1,000 | 0,779 | 0,620 | [-43,5; -1,5; -36,0] | Left Inferior Temporal Gyrus | Not in atlas |
| 3 | 3,48 | 0,000 | 1,000 | 0,779 | 0,620 | [9,0; -81,0; -12,0] | Right Cerebelum 6 | Right Declive |
| 3 | 3,35 | 0,001 | 1,000 | 0,870 | 0,620 | [-52,5; -66,0; -4,5] | Left Inferior Temporal Gyrus | Left Middle Occipital Gyrus |
| 3 | 3,28 | 0,001 | 1,000 | 0,975 | 0,620 | [66,0; -10,5; -22,5] | Right Middle Temporal Gyrus | Not in atlas |
| 3 | 3,27 | 0,001 | 1,000 | 0,975 | 0,620 | [9,0; -87,0; -12,0] | Right Crus 1 Cerebelum | Right Lingual Gyrus |
| 4 | 3,41 | 0,000 | 1,000 | 0,779 | 0,560 | [15,0; -73,5; 45,0] | Right Cuneus | Right Precuneus |
| 5 | 3,50 | 0,000 | 1,000 | 0,779 | 0,510 | [13,5; -1,5; -16,5] | Right ParaHippocampal Gyrus | Right Parahippocampal Gyrus |
| 5 | 3,46 | 0,000 | 1,000 | 0,779 | 0,510 | [7,5; 1,5; -21,0] | Not in atlas | Not in atlas |
| 5 | 3,41 | 0,000 | 1,000 | 0,779 | 0,510 | [61,5; -18,0; 45,0] | Right Postcentral Gyrus | Not in atlas |
| 7 | 4,12 | 0,000 | 0,975 | 0,563 | 0,431 | [-30,0; 45,0; 6,0] | Left Middle Frontal Gyrus | Left Sub-Gyral |
| 7 | 3,54 | 0,000 | 1,000 | 0,779 | 0,431 | [60,0; 9,0; -27,0] | Right Middle Temporal Pole | Not in atlas |
| 7 | 3,51 | 0,000 | 1,000 | 0,779 | 0,431 | [16,5; -94,5; 24,0] | Right Superior Occipital Gyrus | Right Cuneus |
| 8 | 3,71 | 0,000 | 1,000 | 0,779 | 0,398 | [60,0; 28,5; 1,5] | Right Triangular Inferior Frontal Gyrus | Right Inferior Frontal Gyrus |
| 8 | 3,43 | 0,000 | 1,000 | 0,779 | 0,398 | [30,0; -55,5; -24,0] | Right Cerebelum 6 | Right Culmen |
| 12 | 4,09 | 0,000 | 0,983 | 0,563 | 0,299 | [7,5; 51,0; 6,0] | Right Anterior Cingulum | Right Medial Frontal Gyrus |
| 12 | 3,56 | 0,000 | 1,000 | 0,779 | 0,299 | [54,0; 25,5; -12,0] | Right Orbital Inferior Frontal Gyrus | Right Inferior Frontal Gyrus |
| 13 | 3,97 | 0,000 | 0,997 | 0,583 | 0,280 | [-13,5; -1,5; 75,0] | Not in atlas | Not in atlas |
| 13 | 3,64 | 0,000 | 1,000 | 0,779 | 0,280 | [64,5; 10,5; 18,0] | Right Precentral Gyrus | Not in atlas |
| 13 | 3,57 | 0,000 | 1,000 | 0,779 | 0,280 | [55,5; 4,5; -15,0] | Right Middle Temporal Pole | Right Middle Temporal Gyrus |
| 14 | 4,43 | 0,000 | 0,758 | 0,292 | 0,263 | [18,0; -76,5; -4,5] | Right Lingual Gyrus | Right Lingual Gyrus |
| 16 | 4,03 | 0,000 | 0,992 | 0,569 | 0,232 | [15,0; -40,5; 57,0] | Right Superior Parietal Gyrus | Right Paracentral Lobule |
| 16 | 3,44 | 0,000 | 1,000 | 0,779 | 0,232 | [-24,0; -73,5; -42,0] | Left Cerebelum 8 | Left Inferior Semi-Lunar Lobule |
| 21 | 3,93 | 0,000 | 0,998 | 0,583 | 0,174 | [-3,0; -84,0; -12,0] | Left Crus1 Cerebelum | Not in atlas |
| 39 | 3,82 | 0,000 | 1,000 | 0,717 | 0,071 | [-13,5; -15,0; 39,0] | Left Middle Cingulum | Left Cingulate Gyrus |
| 58 | 4,65 | 0,000 | 0,501 | 0,292 | 0,032 | [-15,0; -30,0; -27,0] | Not in atlas | Not in atlas |
| 101 | 4,54 | 0,000 | 0,629 | 0,292 | 0,007 | [46,5; 7,5; -16,5] | Right Superior Temporal Pole | Right Superior Temporal Gyrus |

**Positive correlations found between GMV and HA**

**Model = M2**
**Smoothing filter: FWHM=5mm**

| Ke | peak T | peak *p* unc | peak *p* FWE | peak *p* FDR | cluster *p* clus | Talairach x,y,z {mm} | Anatomical labels AAL atlas | TD atlas |
|---|---|---|---|---|---|---|---|---|
| 1 | 3,44 | 0,000 | 1,000 | 0,988 | 0,794 | [-42,0; -19,5; -9,0] | Not in atlas | Left Sub-Gyral |
| 1 | 3,36 | 0,001 | 1,000 | 0,988 | 0,794 | [-7,5; -7,5; 76,5] | Not in atlas | Left Superior Frontal Gyrus |
| 1 | 3,26 | 0,001 | 1,000 | 0,988 | 0,794 | [-34,5; -24,0; -3,0] | Not in atlas | Left Extra-Nuclear |
| 1 | 3,25 | 0,001 | 1,000 | 0,988 | 0,794 | [6,0; -36,0; 64,5] | Right Paracentral Lobule | Right Paracentral Lobule |
| 1 | 3,23 | 0,001 | 1,000 | 0,988 | 0,794 | [4,5; -6,0; 75,0] | Not in atlas | Right Superior Frontal Gyrus |
| 1 | 3,23 | 0,001 | 1,000 | 0,988 | 0,794 | [4,5; -9,0; 13,5] | Not in atlas | Right Superior Frontal Gyrus |
| 1 | 3,21 | 0,001 | 1,000 | 0,988 | 0,794 | [-42,0; -25,5; 0,0] | Not in atlas | Left Sub-Gyral |
| 1 | 3,20 | 0,001 | 1,000 | 0,988 | 0,794 | [4,5; -42,0; 63,0] | Right Precuneus | Right Postcentral Gyrus |
| 1 | 3,19 | 0,001 | 1,000 | 0,990 | 0,794 | [39,0; -67,5; 3,0] | Right Middle Occipital Gyrus | Right Middle Occipital Gyrus |
| 2 | 3,36 | 0,001 | 1,000 | 0,988 | 0,694 | [42,0; -16,5; 39,0] | Right Precentral Gyrus | Right Precentral Gyrus |
| 2 | 3,32 | 0,001 | 1,000 | 0,988 | 0,694 | [30,0; 33,0; 45,0] | Right Middle Frontal Gyrus | Right Middle Frontal Gyrus |
| 2 | 3,30 | 0,001 | 1,000 | 0,988 | 0,694 | [-48,0; 36,0; 13,5] | Left Triangular Inferior Frontal Gyrus | Left Inferior Frontal Gyrus |
| 2 | 3,23 | 0,001 | 1,000 | 0,988 | 0,694 | [-37,5; -6,0; 55,5] | Left Precentral Gyrus | Left Precentral Gyrus |
| 2 | 3,21 | 0,001 | 1,000 | 0,988 | 0,694 | [37,5; -10,5; 9,0] | Right Insula | Right Insula |
| 3 | 3,33 | 0,001 | 1,000 | 0,988 | 0,619 | [-12,0; 49,5; 27,0] | Left Superior Frontal Gyrus | Left Superior Frontal Gyrus |
| 3 | 3,31 | 0,001 | 1,000 | 0,988 | 0,619 | [36,0; 19,5; 9] | Right Insula | Right Insula |
| 4 | 3,51 | 0,000 | 1,000 | 0,988 | 0,560 | [-6,0; -13,5; 48,0] | Left Supplementary Motor Area | Left Medial Frontal Gyrus |
| 4 | 3,26 | 0,001 | 1,000 | 0,988 | 0,560 | [-10,5; 34,5; 39,0] | Left Medial Superior Frontal Gyrus | Left Medial Frontal Gyrus |
| 6 | 3,39 | 0,001 | 1,000 | 0,988 | 0,468 | [-12,0; 52,5; 10,5] | Left Medial Superior Frontal Gyrus | Left Medial Frontal Gyrus |
| 7 | 3,55 | 0,000 | 1,000 | 0,988 | 0,431 | [55,5; 12,0; 28,5] | Right Precentral Gyrus | Right Inferior Frontal Gyrus |
| 7 | 3,44 | 0,000 | 1,000 | 0,988 | 0,431 | [6,0; -25,5; 33,0] | Right Middle Cingulum | Right Cingulate Gyrus |
| 8 | 3,55 | 0,000 | 1,000 | 0,988 | 0,398 | [-46,5; -43,5; 52,5] | Left Inferior Parietal Gyrus | Left Inferior Parietal Gyrus |
| 8 | 3,34 | 0,001 | 1,000 | 0,988 | 0,398 | [48,0; -6,0; -24,0] | Right Middle Temporal Gyrus | Right Fusiform Gyrus |
| 9 | 3,50 | 0,000 | 1,000 | 0,988 | 0,369 | [-49,5; -55,5; -31,5] | Left Crus 1 Cerebelum | Left Cerebellar Tonsil |
| 10 | 3,56 | 0,000 | 1,000 | 0,988 | 0,343 | [-19,5; -43,5; 58,5] | Left Superior Parietal Gyrus | Left Sub-Gyral |
| 11 | 4,06 | 0,000 | 0,989 | 0,834 | 0,320 | [6,0; -66,0; 51,0] | Right Precuneus | Right Precuneus |
| 11 | 3,91 | 0,000 | 0,999 | 0,834 | 0,320 | [-66,0; -33,0; -24,0] | Not in atlas | Not in atlas |
| 11 | 3,83 | 0,000 | 1,000 | 0,834 | 0,320 | [-13,5; 0,0; 67,5] | Left Superior Frontal Gyrus | Left Superior Frontal Gyrus |
| 16 | 3,93 | 0,000 | 0,999 | 0,834 | 0,232 | [48,0; -13,5; 45,0] | Right Precuneus | Right Precentral Gyrus |
| 17 | 3,73 | 0,000 | 1,000 | 0,834 | 0,218 | [7,5; 21,0; -15,0] | Right Rectus | Right Subcallosal Gyrus |
| 18 | 3,78 | 0,000 | 1,000 | 0,834 | 0,206 | [36,0; 30,0; 0,0] | Right Triangular Inferior Frontal Gyrus | Right Inferior Frontal Gyrus |
| 26 | 4,02 | 0,000 | 0,994 | 0,834 | 0,133 | [55,5; -16,5; 21,0] | Right Rolandic Operculum | Right Postcentral Gyrus |
| 31 | 3,72 | 0,000 | 1,000 | 0,834 | 0,103 | [-12,0; 34,5; 16,5] | Left Anterior Cingulum | Left Anterior Cingulate Gyrus |
| 32 | 4,31 | 0,000 | 0,877 | 0,834 | 0,098 | [21,0; 60,0; 4,5] | Right Superior Frontal Gyrus | Right Superior Frontal Gyrus |
| 36 | 3,82 | 0,000 | 1,000 | 0,834 | 0,081 | [-28,5; 45,0; 28,5] | Left Middle Frontal Gyrus | Left Superior Frontal Gyrus |
| 86 | 5,27 | 0,000 | 0,078 | 0,070 | 0,011 | [45,0; -7,5; 36,0] | Right Precentral Gyrus | Right Precentral Gyrus |

**Negative correlations found between GMV and HA**

**Model = M2**
**Smoothing filter: FWHM=5mm**

| Ke | peak T | peak *p* unc | peak *p* FWE | peak *p* FDR | cluster *p* clus | Talairach x,y,z {mm} | Anatomical labels AAL atlas | TD atlas |
|---|---|---|---|---|---|---|---|---|
| 1 | 3,32 | 0,001 | 1,000 | 0,812 | 0,794 | [7,5; 1,5; -21,0] | Not in atlas | Not in atlas |
| 1 | 3,26 | 0,001 | 1,000 | 0,836 | 0,794 | [15,0; -25,5; 39,0] | Not in atlas | Right Cingulate Gyrus |
| 2 | 3,39 | 0,001 | 1,000 | 0,803 | 0,694 | [-43,5; -1,5; -36,0] | Left Inferior Temporal Gyrus | Not in atlas |
| 2 | 3,39 | 0,001 | 1,000 | 0,803 | 0,694 | [22,5; -24,0; -10,5] | Not in atlas | Right Parahippocampal Gyrus |
| 3 | 3,58 | 0,000 | 1,000 | 0,803 | 0,619 | [-3,0; -72,0; 37,5] | Left Cuneus | Left Precuneus |
| 3 | 3,45 | 0,000 | 1,000 | 0,803 | 0,619 | [15,0; -73,5; 45,0] | Right Cuneus | Right Precuneus |
| 3 | 3,44 | 0,000 | 1,000 | 0,803 | 0,619 | [61,5; -18,0; 45,0] | Right Postcentral Gyrus | Not in atlas |
| 3 | 3,44 | 0,000 | 1,000 | 0,803 | 0,619 | [9,0; -81,0; -12,0] | Right Cerebelum 6 | Right Declive |
| 3 | 3,38 | 0,001 | 1,000 | 0,803 | 0,619 | [16,5; -39,0; 49,5] | Right Paracentral Lobule | Right Paracentral Lobule |
| 3 | 3,36 | 0,001 | 1,000 | 0,803 | 0,619 | [60,0; 9,0; -27,0] | Right Middle Temporal Pole | Not in atlas |
| 3 | 3,29 | 0,001 | 1,000 | 0,836 | 0,619 | [13,5; -1,5; -16,5] | Right ParaHippocampal Gyrus | Right Parahippocampal Gyrus |
| 3 | 3,26 | 0,001 | 1,000 | 0,836 | 0,619 | [-52,5; -66,0; -4,5] | Left Inferior Temporal Gyrus | Left Middel Occipital Gyrus |
| 4 | 3,34 | 0,001 | 1,000 | 0,803 | 0,560 | [16,5; -94,5; 24,0] | Right Superior Occipital Gyrus | Right Cuneus |
| 5 | 3,63 | 0,000 | 1,000 | 0,803 | 0,510 | [60,0; 28,5; 0,0] | Right Triangular Inferior Frontal Gyrus | Right Inferior Frontal Gyrus |
| 6 | 3,34 | 0,001 | 1,000 | 0,803 | 0,468 | [30,0; -55,5; -24,0] | Right Cerebelum 6 | Right Culmen |
| 8 | 4,15 | 0,000 | 0,967 | 0,386 | 0,398 | [-30,0; 45,0; 6,0] | Left Middle Frontal Gyrus | Left Sub-Gyral |
| 8 | 3,37 | 0,001 | 1,000 | 0,803 | 0,398 | [-24,0; -73,5; -42,0] | Left Cerebelum 8 | Left Inferior Semi-Lunar Lobule |
| 9 | 3,46 | 0,000 | 1,000 | 0,803 | 0,369 | [64,5; 12,0; 18,0] | Right Precentral Gyrus | Not in atlas |
| 11 | 4,05 | 0,000 | 0,990 | 0,425 | 0,320 | [7,5; 51,0; 6,0] | Right Anterior Cingulum | Right Medial Frontal Gyrus |
| 13 | 3,52 | 0,000 | 1,000 | 0,803 | 0,280 | [-36,0; 10,5; -24,0] | Left Superior Temporal Pole | Left Superior Temporal Gyrus |
| 14 | 4,47 | 0,000 | 0,722 | 0,374 | 0,263 | [18,0; -76,5; -4,5] | Right Lingual Gyrus | Right Lingual Gyrus |
| 14 | 3,98 | 0,000 | 0,996 | 0,457 | 0,263 | [15,0; -40,5; 57,0] | Right Superior Parietal Gyrus | Right Paracentral Lobule |
| 14 | 3,77 | 0,000 | 1,000 | 0,739 | 0,263 | [-3,0; -84,0; -12,0] | Left Crus 1 Cerebelum | Not in atlas |
| 15 | 3,55 | 0,000 | 1,000 | 0,803 | 0,246 | [52,5; 25,5; -13,5] | Right Orbital Inferior Frontal Gyrus | Right Inferior Frontal Gyrus |
| 16 | 4,24 | 0,000 | 0,925 | 0,378 | 0,232 | [-13,5; -1,5; 75,0] | Not in atlas | Not in atlas |
| 16 | 3,58 | 0,000 | 1,000 | 0,803 | 0,232 | [55,5; 4,5; -15,0] | Right Middle Temporal Pole | Right Middle Temporal Gyrus |
| 38 | 4,48 | 0,000 | 0,702 | 0,374 | 0,074 | [-15,0; -30,0; -27,0] | Not in atlas | Not in atlas |
| 60 | 4,12 | 0,000 | 0,975 | 0,386 | 0,029 | [-12,0; -13,5; 39,0] | Left Middle Cingulum | Left Cingulate Gyrus |
| 90 | 4,41 | 0,000 | 0,784 | 0,374 | 0,010 | [46,5; 7,5; -16,5] | Right Superior Temporal Pole | Right Superior Temporal Gyrus |

**Positive correlations found between GMV and HA**

**Model = M3**
**Smoothing filter: FWHM=5mm**

| Ke | peak T | peak *p* unc | peak *p* FWE | peak *p* FDR | cluster *p* clus | Talairach x,y,z {mm} | Anatomical labels: AAL atlas | Anatomical labels: TD atlas |
|---|---|---|---|---|---|---|---|---|
| 1 | 3,38 | 0,001 | 1,000 | 0,993 | 0,794 | [16,5; 28,5; -1,5] | Not in atlas | Right Sub-Gyral |
| 1 | 3,33 | 0,001 | 1,000 | 0,993 | 0,794 | [-66,0; -33,0; -24,0] | Not in atlas | Not in atlas |
| 1 | 3,28 | 0,001 | 1,000 | 0,993 | 0,794 | [-42,0; 39,0; -10,5] | Left Orbital Inferior Frontal Gyrus | Left Middle Frontal Gyrus |
| 1 | 3,25 | 0,001 | 1,000 | 0,993 | 0,794 | [10,5; 52,5; -9,0] | Right Orbital Medial Frontal Gyrus | Right Medial Frontal gyrus |
| 1 | 3,24 | 0,001 | 1,000 | 0,993 | 0,794 | [-45,0; -42,0; 51,0] | Left Inferior Parietal Gyrus | Left Inferior Parietal Lobule |
| 1 | 3,23 | 0,001 | 1,000 | 0,993 | 0,794 | [-18,0; 60,0; 3,0] | Left Superior Frontal Gyrus | Left Medial Frontal Gyrus |
| 1 | 3,23 | 0,001 | 1,000 | 0,993 | 0,794 | [54,0; -18,0; 21,0] | Right Rolandic operculum | Right Postcentral Gyrus |
| 1 | 3,22 | 0,001 | 1,000 | 0,993 | 0,794 | [-25,5; 19,5; -15,0] | Left Insula | Left Inferior Frontal Gyrus |
| 1 | 3,21 | 0,001 | 1,000 | 0,993 | 0,794 | [-45,0; -22,5; -1,5] | Left Middle Temporal Gyrus | Left Superior Temporal Gyrus |
| 1 | 3,20 | 0,001 | 1,000 | 0,993 | 0,794 | [-7,5; 58,5; -6,0] | Left Orbital Medial Frontal Gyurs | Left Medial Frontal Gyrus |
| 1 | 3,20 | 0,001 | 1,000 | 0,993 | 0,794 | [48,0; -13,5; 46,5] | Right Precentral Gyrus | Right Precentral Gyrus |
| 1 | 3,19 | 0,001 | 1,000 | 0,993 | 0,794 | [-7,5; -27,0; 33,0] | Left Middle Cingulum | Left Cingulum Gyrus |
| 1 | 3,19 | 0,001 | 1,000 | 0,993 | 0,794 | [15,0; 37,5; 39,0] | Right Superior Frontal gyrus | Right Superior Frontal Gyrus |
| 2 | 3,41 | 0,000 | 1,000 | 0,993 | 0,694 | [42,0; 60,0; 7,5] | Not in atlas | Not in atlas |
| 2 | 3,35 | 0,001 | 1,000 | 0,993 | 0,694 | [12,0; -85,5; 28,5] | Right Cunues | Right Cuneus |
| 2 | 3,35 | 0,001 | 1,000 | 0,993 | 0,694 | [43,5; -6,0; -19,5] | Not in atlas | Right Sub-Gyral |
| 2 | 3,27 | 0,001 | 1,000 | 0,993 | 0,694 | [28,5; -6,0; -36,0] | Right Fusiform Gyrus | Right Uncus |
| 3 | 3,58 | 0,000 | 1,000 | 0,900 | 0,619 | [25,5; 67,5; -10,5] | Not in atlas | Not in atlas |
| 3 | 3,49 | 0,000 | 1,000 | 0,984 | 0,619 | [10,5; 37,5; -30,0] | Not in atlas | Right Orbital Gyrus |
| 3 | 3,31 | 0,001 | 1,000 | 0,993 | 0,619 | [36,0; -48,0; -13,5] | Right Fusiform Gyrus | Right Fusiform Gyrus |
| 6 | 3,58 | 0,000 | 1,000 | 0,900 | 0,467 | [-36,0; 43,5; -7,5] | Left Orbital Middle Frontal Gyrus | Left Middle Frontal Gyrus |
| 7 | 3,93 | 0,000 | 0,998 | 0,677 | 0,430 | [6,0; -66,0; 51,0] | Right Precuneus | Right Precuneus |
| 7 | 3,70 | 0,000 | 1,000 | 0,900 | 0,430 | [30,0; 34,5; 43,5] | Right Middle Frontal Gyrus | Right Middle Frontal Gyrus |
| 7 | 3,63 | 0,000 | 1,000 | 0,900 | 0,430 | [-4,5; -5,0; 42,0] | Left Middle Cingulum | Left Cingulate Gyrus |
| 7 | 3,36 | 0,001 | 1,000 | 0,993 | 0,430 | [9,0; -6,0; 15,0] | Not in atlas | Right Lateral Ventricle |
| 10 | 3,91 | 0,000 | 0,999 | 0,677 | 0,343 | [21,0; 30,0; -10,5] | Not in atlas | Right Sub-Gyral |
| 11 | 4,20 | 0,000 | 0,947 | 0,522 | 0,320 | [-21,0; -34,5; 57,0] | Not in atlas | Left Postcentral Gyrus |
| 11 | 3,57 | 0,000 | 1,000 | 0,900 | 0,320 | [-19,5; -43,5; 58,5] | Left Superior Parietal Gyrus | Left Sub-Gyral |
| 15 | 3,67 | 0,000 | 1,000 | 0,900 | 0,246 | [-13,5; 54,0; 13,5] | Left Superior Frontal Gyrus | Left Superior Frontal Gyrus |
| 18 | 4,14 | 0,000 | 0,972 | 0,529 | 0,206 | [6,0; -25,5; 33,0] | Right Middle Cingulum | Right Cingulate Gyrus |
| 23 | 4,30 | 0,000 | 0,889 | 0,489 | 0,155 | [55,5; 12,0; 28,5] | Right Precentral Gyrus | Right Inferior Frontal Gyrus |
| 37 | 4,72 | 0,000 | 0,431 | 0,251 | 0,077 | [21,0; 58,5; 4,5] | Right Superior Frontal gyrus | Right Superior Frontal Gyrus |
| 37 | 4,05 | 0,000 | 0,990 | 0,592 | 0,077 | [48,0; -6,0; -24,0] | Right Middle Temporal Gyrus | Right Fusiform Gyrus |
| 37 | 3,66 | 0,000 | 1,000 | 0,900 | 0,077 | [-28,5; 42,0; 27,0] | Left Middle Frontal Gyrus | Left Superior Frontal Gyrus |
| 87 | 4,55 | 0,000 | 0,630 | 0,295 | 0,011 | [-12,0; 34,5; 16,5] | Left Anterior Cingulum | Left Anterior Cingulate Gyrus |
| 133 | 4,73 | 0,000 | 0,425 | 0,251 | 0,002 | [45,0; -7,5; 34,5] | Right Precentral Gyrus | Right Precentral Gyrus |

**Negative correlations found between GMV and HA**

**Model = M3**
**Smoothing filter: FWHM=5mm**

| Ke | peak T | peak *p* unc | peak *p* FWE | peak *p* FDR | cluster *p* clus | Talairach x,y,z {mm} | Anatomical labels: AAL atlas | Anatomical labels: TD atlas |
|---|---|---|---|---|---|---|---|---|
| 1 | 3,56 | 0,000 | 1,000 | 0,703 | 0,794 | [24,0; -10,5; -7,5] | Not in atlas | Not in atlas |
| 1 | 3,52 | 0,000 | 1,000 | 0,703 | 0,794 | [-64,5; -34,5; 18,0] | Left Superior Temporal Gyrus | Left Superior Temporal Gyrus |
| 1 | 3,26 | 0,001 | 1,000 | 0,892 | 0,794 | [16,5; -76,5; -4,5] | Right Lingual Gyrs | Right Lingual Gyrus |
| 1 | 3,24 | 0,001 | 1,000 | 0,899 | 0,794 | [6,0; -93,0; -4,5] | Not in atlas | Right Lingual Gyrus |
| 2 | 3,40 | 0,000 | 1,000 | 0,723 | 0,694 | [30,0; -54,0; -24,0] | Right Cerebelum 6 | Right Culmen |
| 3 | 3,29 | 0,001 | 1,000 | 0,863 | 0,619 | [69,0; -18,0; -1,50] | Right Superior Temporal Gyrus | Not in atlas |
| 3 | 3,23 | 0,001 | 1,000 | 0,899 | 0,619 | [9,0; -67,5; 63,0] | Right Precuneus | Not in atlas |
| 4 | 3,70 | 0,000 | 1,000 | 0,703 | 0,560 | [60,0; 28,5; 0,0] | Right Triangular Inferior Frontal Gyrus | Right Inferior Fronatl Gyrus |
| 4 | 3,44 | 0,000 | 1,000 | 0,703 | 0,560 | [66,0; 3,0; -4,5] | Right Superior Temporal Pole | Not in atlas |
| 4 | 3,38 | 0,001 | 1,000 | 0,723 | 0,560 | [-60,0; -27,0; -21,0] | Left Inferior Temporal Gyrus | Left Inferior Temporal Gyrus |
| 5 | 3,45 | 0,000 | 1,000 | 0,703 | 0,510 | [64,5; 12,0; 16,5] | Right Precentral Gyrus | Not in atlas |
| 6 | 3,57 | 0,000 | 1,000 | 0,703 | 0,467 | [-15,0; -30,0; -27,0] | Not in atlas | Not in atlas |
| 7 | 3,77 | 0,000 | 1,000 | 0,703 | 0,430 | [7,5; 52,5; 7,5] | Right Anterior Cingulum | Right Medial Frontal Gyrus |
| 8 | 3,69 | 0,000 | 1,000 | 0,703 | 0,398 | [16,5; -94,5; 24,0] | Right Superior Occipital Gyrus | Right Cuneus |
| 8 | 3,64 | 0,000 | 1,000 | 0,703 | 0,398 | [66,0; -39,0; 12,0] | Right Middle Temporal Gyrus | Right Superior Temporal Gyrus |
| 9 | 3,48 | 0,000 | 1,000 | 0,703 | 0,369 | [-3,0; -84,0; -10,5] | Left Crus 1 Cerebelum | Not in atlas |
| 9 | 3,46 | 0,000 | 1,000 | 0,703 | 0,369 | [42,0; -12,0; 64,5] | Not in atlas | Not in atlas |
| 13 | 3,56 | 0,000 | 1,000 | 0,703 | 0,280 | [51,0; 1,5; 48,0] | Right Middle Frontal Gyrus | Right Precentral Gyrus |
| 14 | 3,63 | 0,000 | 1,000 | 0,703 | 0,262 | [60,0; 10,5; -21,0] | Right Middle Temporal Pole | Not in atlas |
| 14 | 3,63 | 0,000 | 1,000 | 0,703 | 0,262 | [-48,0; 18,0; -10,5] | Left Superior Temporal Pole | Left Superior Temporal Gyrus |
| 15 | 3,53 | 0,000 | 1,000 | 0,703 | 0,246 | [52,5; 24,0; -13,5] | Right Orbital Inferior Frontal Gyrus | Right Inferior Frontal Gyrus |
| 18 | 3,98 | 0,000 | 0,997 | 0,538 | 0,206 | [13,5; -40,5; 58,5] | Right Superior Parietal Gyrus | Right Sub-Gyral |
| 18 | 3,62 | 0,000 | 1,000 | 0,703 | 0,206 | [46,5; 7,5; -16,5] | Right Superior Temporal Pole | Right Superior Temporal Gyrus |
| 27 | 3,97 | 0,000 | 0,997 | 0,538 | 0,126 | [10,5; 1,5; -21,0] | Not in atlas | Not in atlas |
| 34 | 4,17 | 0,000 | 0,960 | 0,538 | 0,089 | [-3,0; -72,0; 39,0] | Left Precuneus | Left Precuneus |
| 73 | 4,78 | 0,000 | 0,370 | 0,338 | 0,018 | [16,5; -73,5; 45,0] | Right Cuneus | Right Precuneus |
| 75 | 4,24 | 0,000 | 0,929 | 0,538 | 0,017 | [55,5; 4,5; -15,0] | Right Middle Temporal Pole | Right Middle Temporal Gyrus |
| 188 | 4,11 | 0,000 | 0,980 | 0,538 | 0,001 | [-24,0; -75,0; -42,0] | Not in atlas | Left Inferior Semi-Lunar Lobule |

| Positive correlations found between GMV and HA | | | | | | | | |
|---|---|---|---|---|---|---|---|---|
| Model = M4 | | | | | | | | |
| Smoothing filter: FWHM=5mm | | | | | | | | |
| | peak | peak | peak | peak | cluster | Talairach | Anatomical labels | |
| Ke | T | p unc | p FWE | p FDR | p clus | x,y,z {mm} | AAL atlas | TD atlas |
| 1 | 3,48 | 0,000 | 1,000 | 0,930 | 0,793 | [-42,0; 39,0; -10,5] | Left Orbital Inferior Frontal Gyrus | Left Middle Frontal Gyrus |
| 1 | 3,35 | 0,001 | 1,000 | 0,930 | 0,793 | [-6,0; 39,0; -4,5] | Left Anterior Cingulum | Left Anterior Cingulate Gyrus |
| 1 | 3,32 | 0,001 | 1,000 | 0,930 | 0,793 | [30,0; 34,5; 43,5] | Right Middle Frontal Gyrus | Right Middle Frontal Gyrus |
| 1 | 3,31 | 0,001 | 1,000 | 0,930 | 0,793 | [25,5; 67,5; -10,5] | Not in atlas | Not in atlas |
| 1 | 3,28 | 0,001 | 1,000 | 0,930 | 0,793 | [10,5; 52,5; -9,0] | Right Orbital Medial Frontal Gyrus | Right Medial Frontal Gyrus |
| 1 | 3,28 | 0,001 | 1,000 | 0,930 | 0,793 | [-10,5; 34,5; 39,0] | Left Medial Superior Frontal Gyrus | Left Medial Frontal Gyrus |
| 1 | 3,23 | 0,001 | 1,000 | 0,948 | 0,793 | [-54,0; -27,0; 19,5] | Left SupraMarginal Gyrus | Left Postcentral Gyrus |
| 1 | 3,22 | 0,001 | 1,000 | 0,948 | 0,793 | [49,5; -16,5; -18,0] | Not in atlas | Right Sub-Gyral |
| 1 | 3,22 | 0,001 | 1,000 | 0,948 | 0,793 | [27,0; -4,5; -45,0] | Not in atlas | Not in atlas |
| 1 | 3,21 | 0,001 | 1,000 | 0,948 | 0,793 | [-7,5; -82,5; -15,0] | Left Crus 1 Cerebelum | Not in atlas |
| 2 | 3,60 | 0,000 | 1,000 | 0,803 | 0,691 | [16,5; 28,5; -3,0] | Not in atlas | Right Sub-Gyral |
| 2 | 3,54 | 0,000 | 1,000 | 0,856 | 0,691 | [-43,5; -7,5; -19,5] | Not in atlas | Left Sub-Gyral |
| 2 | 3,46 | 0,000 | 1,000 | 0,930 | 0,691 | [-16,5; -91,5; -6,0] | Left Lingual | Left Lingual Gyrus |
| 2 | 3,35 | 0,001 | 1,000 | 0,930 | 0,691 | [-66,0; -33,0; -24,0] | Not in atlas | Not in atlas |
| 2 | 3,35 | 0,001 | 1,000 | 0,930 | 0,691 | [-18,0; 1,5; 60,0] | Left Supplementary Motor Area | Left Middle Frontal Gyrus |
| 2 | 3,28 | 0,001 | 1,000 | 0,930 | 0,691 | [-42,0; -18,0; -10,5] | Not in atlas | Left Sub-Gyral |
| 2 | 3,27 | 0,001 | 1,000 | 0,930 | 0,691 | [-43,5; -22,5; -1,5] | Not in atlas | Left Superior Temporal Gyrus |
| 2 | 3,24 | 0,001 | 1,000 | 0,948 | 0,691 | [15,0; 37,5; 39,0] | Right Superior Frontal Gyrus | Right Superior Frontal Gyrus |
| 3 | 3,35 | 0,001 | 1,000 | 0,930 | 0,617 | [-34,5; -24,0; -3,0] | Not in atlas | Left Ectra-Nuclear |
| 3 | 3,31 | 0,001 | 1,000 | 0,930 | 0,617 | [-36,0; -12,0; -12,0] | Not in atlas | Left Sub-Gyral |
| 4 | 3,33 | 0,001 | 1,000 | 0,930 | 0,557 | [-7,5; -25,5; 33,0] | Left Middle Cingulum | Left Cingulate Gyrus |
| 5 | 3,40 | 0,001 | 1,000 | 0,930 | 0,507 | [-16,5; 60,0; 3,0] | Left Superior Frontal Gyrus | Left Medial Frontal Gyrus |
| 6 | 3,84 | 0,000 | 1,000 | 0,646 | 0,464 | [6,0; -66,0; 51,0] | Right Precuneus | Right Precuneus |
| 6 | 3,42 | 0,000 | 1,000 | 0,930 | 0,464 | [24,0; 31,5; -16,5] | Right Orbital Superior Frontal Gyrus | Right Middle Fronatl Gyrus |
| 8 | 3,39 | 0,001 | 1,000 | 0,930 | 0,395 | [-24,0; 24,0; -18,0] | Left Orbital Inferior Frontal Gyrus | Left Middle Frontal Gyrus |
| 9 | 3,71 | 0,000 | 1,000 | 0,702 | 0,366 | [21,0; 58,5; 4,5] | Right Superior Frontal Gyrus | Right Superior Frontal Gyrus |
| 10 | 4,22 | 0,000 | 0,945 | 0,579 | 0,340 | [-21,0; -34,5; 57,0] | Not in atlas | Left Postcentral Gyrus |
| 10 | 4,01 | 0,000 | 0,996 | 0,579 | 0,340 | [-36,0; 43,5; -7,5] | Left Orbital Middle Frontal Gyrus | Left Middle Frontal Gyrus |
| 10 | 3,68 | 0,000 | 1,000 | 0,702 | 0,340 | [-10,5; -48,0; 37,5] | Left Precuneus | Left Precuneus |
| 11 | 3,54 | 0,000 | 1,000 | 0,856 | 0,317 | [-10,5; 54,0; 13,5] | Left Medial Superior Frontal Gyrus | Left Medial Frontal Gyrus |
| 17 | 3,72 | 0,000 | 1,000 | 0,702 | 0,215 | [52,5; 0,0; 25,5] | Right Postcentral Gyrus | Right Precentral Gyrus |
| 17 | 3,70 | 0,000 | 1,000 | 0,702 | 0,215 | [21,0; 30,0; -10,5] | Not in atlas | Right Sub-Gyral |
| 18 | 4,38 | 0,000 | 0,832 | 0,579 | 0,202 | [4,5; -25,5; 31,5] | Right Middle Cingulum | Right Cingulate Gyrus |
| 20 | 4,57 | 0,000 | 0,628 | 0,579 | 0,180 | [-28,5; 12,0; 64,5] | Not in atlas | Not in atlas |
| 22 | 4,11 | 0,000 | 0,984 | 0,579 | 0,161 | [-9,0; 51,0; 30,0] | Left Medial Superior Frontal Gyrus | Left Superior Frontal Gyrus |
| 28 | 4,07 | 0,000 | 0,990 | 0,579 | 0,117 | [13,50; -81,0; 1,5] | Right Lingual Gyrus | Right Lingual Gyrus |
| 31 | 4,23 | 0,000 | 0,944 | 0,579 | 0,101 | [28,5; -4,5; -36,0] | Right Fusiform gyrus | Rigt Uncus |
| 34 | 4,04 | 0,000 | 0,993 | 0,579 | 0,087 | [48,0; -6,0; -24,0] | Right Middle Temporal Gyrus | Right Fusiform Gyrus |
| 56 | 3,90 | 0,000 | 0,999 | 0,646 | 0,033 | [54,0; 10,5; 28,5] | Right Precentral Gyrus | Right Inferior Frontal Gyrus |
| 65 | 4,18 | 0,000 | 0,965 | 0,579 | 0,023 | [-12,0; 36,0; 15,0] | Not in atlas | Left Anterior Cingulate Gyrus |
| 90 | 3,83 | 0,000 | 1,000 | 0,646 | 0,009 | [40,5; -10,5; 34,5] | Not in atlas | Right Precentral Gyrus |

| Negative correlations found between GMV and HA | | | | | | | | |
|---|---|---|---|---|---|---|---|---|
| Model = M4 | | | | | | | | |
| Smoothing filter: FWHM=5mm | | | | | | | | |
| | peak | peak | peak | peak | cluster | Talairach | Anatomical labels | |
| Ke | T | p unc | p FWE | p FDR | p clus | x,y,z {mm} | AAL atlas | TD atlas |
| 1 | 3,31 | 0,001 | 1,000 | 0,992 | 0,793 | [48,0; 6,0; -16,5] | Right Superior Temporal Pole | Right Middle Temporal Gyrus |
| 1 | 3,26 | 0,001 | 1,000 | 0,992 | 0,793 | [67,5; -19,5; -22,5] | Right Inferior Temporal Gyrus | Not in atlas |
| 1 | 3,25 | 0,001 | 1,000 | 0,992 | 0,793 | [-49,5; 19,5; -10,5] | Left Superior Temporal Pole | Left Superior Temporal Gyrus |
| 1 | 3,25 | 0,001 | 1,000 | 0,992 | 0,793 | [13,5; -40,5; 58,5] | Right Superior Parietal Gyrus | Right Sub-Gyral |
| 1 | 3,24 | 0,001 | 1,000 | 0,992 | 0,793 | [-37,5; -78,0; -36,0] | Left Crus 2 Cerebelum | Left Inferior Semi-Lunar Lobule |
| 1 | 3,23 | 0,001 | 1,000 | 0,992 | 0,793 | [4,5; -54,0; 40,5] | Right Precuneus | Right Precuneus |
| 1 | 3,20 | 0,001 | 1,000 | 0,992 | 0,793 | [60,0; 9,0; -28,5] | Not in atlas | Not in atlas |
| 1 | 3,19 | 0,001 | 1,000 | 0,992 | 0,793 | [64,5; 12,0; 18,0] | Right Precentral Gyrus | Not in atlas |
| 2 | 3,35 | 0,001 | 1,000 | 0,992 | 0,691 | [-9,0; -55,5; -34,5] | Left Cerebelum 9 | Left Cerebellar Tonsil |
| 2 | 3,31 | 0,001 | 1,000 | 0,992 | 0,691 | [-64,5; -34,5; 18,0] | Left Superior Temporal Gyrus | Left Superior Temporal Gyrus |
| 2 | 3,24 | 0,001 | 1,000 | 0,992 | 0,691 | [33,0; 4,5; -1,5] | Right Putamen | Right Claustrum |
| 2 | 3,20 | 0,001 | 1,000 | 0,992 | 0,691 | [-18,0; -28,5; -33,0] | Not in atlas | Not in atlas |
| 3 | 3,42 | 0,000 | 1,000 | 0,992 | 0,617 | [7,5; -43,5; -37,5] | Not in atlas | Not in atlas |
| 3 | 3,35 | 0,001 | 1,000 | 0,992 | 0,617 | [7,5; 51,0; 6,0] | Right Anterior Cingulum | Right Medial Frontal Gyrus |
| 3 | 3,27 | 0,001 | 1,000 | 0,992 | 0,617 | [36,0; -61,5; -25,5] | Right Crus 1 Cerebelum | Right Tuber |
| 4 | 3,75 | 0,000 | 1,000 | 0,992 | 0,557 | [60,0; 28,5; 0,0] | Right Triangular Inferior Frontal Gyrus | Right Inferior Frontal Gyrus |
| 4 | 3,53 | 0,000 | 1,000 | 0,992 | 0,557 | [16,5; -76,5; -4,5] | Right Lingual Gyrus | Right Lingual Gyrus |
| 4 | 3,51 | 0,000 | 1,000 | 0,992 | 0,557 | [-64,5; -3,0; -1,5] | Not in atlas | Left Superior Temporal Gyrus |
| 4 | 3,42 | 0,000 | 1,000 | 0,992 | 0,557 | [60,0; 1,5; -36,0] | Not in atlas | Not in atlas |
| 4 | 3,34 | 0,001 | 1,000 | 0,992 | 0,557 | [-18,0; -84,0; -45,0] | Not in atlas | Not in atlas |
| 4 | 3,34 | 0,001 | 1,000 | 0,992 | 0,557 | [42,0; -46,5; -33,0] | Right Crus 2 Cerebelum | Right Cerebellar Tonsil |
| 4 | 3,23 | 0,001 | 1,000 | 0,992 | 0,557 | [6,0; -72,0; -42,0] | Not in atlas | Not in atlas |
| 5 | 3,46 | 0,000 | 1,000 | 0,992 | 0,507 | [-37,5; -51,0; -31,5] | Left Crus 1 Cerebelum | Left Cerebellar Tonsil |
| 6 | 3,47 | 0,000 | 1,000 | 0,992 | 0,464 | [-1,5; -90,0; 3,0] | Left Calcarine | Left Lingual Gyrus |
| 6 | 3,43 | 0,000 | 1,000 | 0,992 | 0,464 | [9,0; 3,0; -22,5] | Not in atlas | Not in atlas |
| 6 | 3,36 | 0,001 | 1,000 | 0,992 | 0,464 | [49,5; 24,0; -15,0] | Right Orbital Inferior Frontal Gyrus | Not in atlas |
| 7 | 3,62 | 0,000 | 1,000 | 0,992 | 0,427 | [-22,5; 21,0; 55,5] | Left Superior Frontal Gyrus | Left Middle Frontal Gyrus |
| 8 | 3,58 | 0,000 | 1,000 | 0,992 | 0,395 | [-60,0; -27,0; -21,0] | Left Inferior Temporal Gyrus | Left Inferior Temporal Gyrus |
| 9 | 3,69 | 0,000 | 1,000 | 0,992 | 0,366 | [16,5; -96,0; 24,0] | Right Superior Occipital Gyrus | Right Cuneus |
| 9 | 3,62 | 0,000 | 1,000 | 0,992 | 0,366 | [64,5; -57,0; 15,0] | Not in atlas | Right Superior Temporal Gyrus |
| 9 | 3,48 | 0,000 | 1,000 | 0,992 | 0,366 | [28,5; -54,0; -22,5] | Right Cerebelum 6 | Right Culmen |
| 13 | 3,61 | 0,000 | 1,000 | 0,992 | 0,276 | [64,5; 4,5; -6,0] | Right Superior Temporal Pole | Not in atlas |
| 14 | 4,18 | 0,000 | 0,965 | 0,992 | 0,259 | [-3,0; -73,5; 39,0] | Left Cuneus | Left Precuneus |
| 14 | 3,91 | 0,000 | 0,999 | 0,992 | 0,259 | [-15,0; -30,0; -27,0] | Not in atlas | Not in atlas |
| 14 | 3,84 | 0,000 | 1,000 | 0,992 | 0,259 | [49,5; 3,0; 48,0] | Right Middle Frontal Gyrus | Right Precentral Gyrus |
| 15 | 3,81 | 0,000 | 1,000 | 0,992 | 0,243 | [-1,5; -52,5; -49,5] | Not in atlas | Not in atlas |
| 17 | 3,55 | 0,000 | 1,000 | 0,992 | 0,215 | [55,5; 3,0; -13,5] | Right Middle Temporal Pole | Right Temporal Gyrus |
| 21 | 3,69 | 0,000 | 1,000 | 0,992 | 0,170 | [-24,0; -73,5; -42,0] | Left Cerebelum 8 | Left Inferior Semi-Lunar Lobule |
| 66 | 4,89 | 0,000 | 0,294 | 0,321 | 0,022 | [16,5; -73,5; 45,0] | Right Cuneus | Right Precuneus |

| Positive correlations found between GMV and HA | | | | | | | | |
|---|---|---|---|---|---|---|---|---|
| **Model = M1** | | | | | | | | |
| **Smoothing filter: FWHM=8mm** | | | | | | | | |
| | **peak** | **peak** | **peak** | **peak** | **cluster** | **Talairach** | **Anatomical labels** | |
| **Ke** | **T** | ***p* unc** | ***p* FWE** | ***p* FDR** | ***p* clus** | **x,y,z {mm}** | **AAL atlas** | **TD atlas** |
| 1 | 3,23 | 0,001 | 1,000 | 0,933 | 0,902 | [-24,0; 19,5; 4,5] | Not in atlas | Left Extra-Nuclear |
| 1 | 3,22 | 0,001 | 1,000 | 0,933 | 0,902 | [-61,5; -55,5; 18,0] | Not in atlas | Left Superior Temporal Gyrus |
| 1 | 3,21 | 0,001 | 1,000 | 0,933 | 0,902 | [-12,0; -12,0; 16,5] | Left Thalamus | Left Thalamus |
| 2 | 3,29 | 0,001 | 1,000 | 0,933 | 0,849 | [-60,0; -57,0; 21,0] | Not in atlas | Left Superior Temporal Gyrus |
| 2 | 3,27 | 0,001 | 1,000 | 0,933 | 0,849 | [-13,5; -15,0; 18,0] | Left Thalamus | Left Thalamus |
| 3 | 3,36 | 0,001 | 1,000 | 0,913 | 0,807 | [9,0; 21,0; -15,0] | Right Rectus | Right Subcallosal Gyrus |
| 11 | 3,38 | 0,001 | 1,000 | 0,913 | 0,600 | [9,0; 37,5; 30,0] | Right Middle Cingulum | Right Medial Frontal Gyrus |
| 12 | 3,40 | 0,000 | 1,000 | 0,913 | 0,582 | [55,5; -18,0; 19,5] | Right Rolandic Operculum | Right Postcentral Gyrus |
| 12 | 3,35 | 0,001 | 1,000 | 0,913 | 0,582 | [-10,5; 49,5; 28,5] | Left Medial Superior Frontal Gyrus | Left Superior Frontal Gyrus |
| 21 | 3,61 | 0,000 | 0,997 | 0,913 | 0,456 | [-30,0; 43,5; 25,5] | Left Middle Frontal Gyrus | Left Middle Frontal Gyrus |
| 24 | 3,49 | 0,000 | 0,999 | 0,913 | 0,424 | [-21,0; -43,5; 57,0] | Left Superior Parietal Gyrus | Left Sub-Gyral |
| 24 | 3,49 | 0,000 | 0,999 | 0,913 | 0,424 | [54,0; 13,5; 27,0] | Right Opercular Inferior Frontal Gyrus | Left Inferior Frontal Gyrus |
| 25 | 3,54 | 0,000 | 0,999 | 0,913 | 0,414 | [43,5; -6,0; -22,5] | Not in atlas | Right Fusiform Gyrus |
| 29 | 3,56 | 0,000 | 0,998 | 0,913 | 0,377 | [-10,5; 34,5; 18,0] | Left Anterior Cingulum | Left Anterior Cingulate Gyrus |
| 39 | 3,59 | 0,000 | 0,997 | 0,913 | 0,305 | [-6,0; -31,5; 22,5] | Not in atlas | Left Posterior Cingulate |
| 54 | 4,17 | 0,000 | 0,693 | 0,618 | 0,229 | [21,0; 58,5; 6,0] | Right Superior Frontal Gyrus | Right Superior Frontal Gyrus |
| 197 | 4,09 | 0,000 | 0,772 | 0,618 | 0,030 | [36,0; 30,0; 0,0] | Right Triangular Inferior Frontal Gyrus | Right Inferior Frontal Gyrus |
| 346 | 5,07 | 0,000 | 0,059 | 0,077 | 0,006 | [42,0; -7,5; 37,5] | Right Precentral Gyrus | Right Precentral Gyrus |

| Negative correlations found between GMV and HA | | | | | | | | |
|---|---|---|---|---|---|---|---|---|
| **Model = M1** | | | | | | | | |
| **Smoothing filter: FWHM=8mm** | | | | | | | | |
| | **peak** | **peak** | **peak** | **peak** | **cluster** | **Talairach** | **Anatomical labels** | |
| **Ke** | **T** | ***p* unc** | ***p* FWE** | ***p* FDR** | ***p* clus** | **x,y,z {mm}** | **AAL atlas** | **TD atlas** |
| 7 | 3,61 | 0,000 | 0,996 | 0,582 | 0,685 | [-30,0; 43,5; 6,0] | Left Middle Frontal Gyrus | Left Sub-Gyral |
| 12 | 3,48 | 0,000 | 1,000 | 0,582 | 0,582 | [16,5; -76,5; -6,0] | Right Lingual Gyrus | Right Lingual Gyrus |
| 20 | 3,42 | 0,000 | 1,000 | 0,582 | 0,467 | [9,0; -82,5; -12,0] | Right Crus 1 Cerebelum | Not in atlas |
| 22 | 3,60 | 0,000 | 0,997 | 0,582 | 0,445 | [64,5; -9,0; -24,0] | Not in atlas | Not in atlas |
| 22 | 3,52 | 0,000 | 0,999 | 0,582 | 0,445 | [-16,5; -28,5; -27,0] | Not in atlas | Not in atlas |
| 24 | 3,46 | 0,000 | 1,000 | 0,582 | 0,424 | [-12,0; -15,0; 39,0] | Left Middle Cingulum | Left Cingulate Gyrus |
| 24 | 3,41 | 0,000 | 1,000 | 0,582 | 0,424 | [-18,0; -82,5; -42,0] | Not in atlas | Not in atlas |
| 34 | 3,74 | 0,000 | 0,981 | 0,582 | 0,338 | [66,0; 13,5; 13,5] | Right Precentral Gyrus | Not in atlas |
| 66 | 3,59 | 0,000 | 0,997 | 0,582 | 0,185 | [-36,0; 9,0; -24,0] | Left Superior Temporal Pole | Left Supeior Temporal Gyrus |
| 100 | 4,01 | 0,000 | 0,844 | 0,582 | 0,108 | [48,0; 9,0; -16,5] | Right Superior Temporal Pole | Right Superior Temporal Gyrus |

| Positive correlations found between GMV and HA | | | | | | | | |
|---|---|---|---|---|---|---|---|---|
| **Model = M2** | | | | | | | | |
| **Smoothing filter: FWHM=8mm** | | | | | | | | |
| | **peak** | **peak** | **peak** | **peak** | **cluster** | **Talairach** | **Anatomical labels** | |
| **Ke** | **T** | **p unc** | **p FWE** | **p FDR** | **p clus** | **x,y,z {mm}** | **AAL atlas** | **TD atlas** |
| 1 | 3,22 | 0,001 | 1,000 | 0,910 | 0,902 | [-43,5; -7,5; 36,0] | Left Postcentral Gyrus | Left Precentral Gyrus |
| 4 | 3,34 | 0,001 | 1,000 | 0,840 | 0,771 | [9,0; 21,0; -15,0] | Right Rectus | Right Subcallosal Gyrus |
| 7 | 3,34 | 0,001 | 1,000 | 0,840 | 0,685 | [55,5; -18,0; 19,5] | Right Rolandic Operculum | Right Postcentral Gyrus |
| 7 | 3,31 | 0,001 | 1,000 | 0,840 | 0,685 | [10,5; 37,5; 30,0] | Right Middle Cingulum | Right Medial Frontal Gyrus |
| 8 | 3,40 | 0,001 | 1,000 | 0,840 | 0,661 | [-60,0; -57,0; 21,0] | Not in atlas | Left Superior Temporal Gyrus |
| 10 | 3,31 | 0,001 | 1,000 | 0,840 | 0,619 | [54,0; 13,5; 27,0] | Right Opercular Inferior Frontal Gyrus | Right Inferior Frontal Gyrus |
| 12 | 3,48 | 0,000 | 1,000 | 0,840 | 0,581 | [-30,0; 43,5; 25,5] | Left Middle Frontal Gyrus | Left Middle Frontal Gyrus |
| 27 | 3,50 | 0,000 | 0,999 | 0,840 | 0,394 | [43,5; -6,0; -22,5] | Not in atlas | Right Fusiform Gyrus |
| 28 | 3,55 | 0,000 | 0,999 | 0,840 | 0,385 | [-7,5; -31,5; 22,5] | Not in atlas | Left Extra-Nuclear |
| 30 | 3,53 | 0,000 | 0,999 | 0,840 | 0,368 | [-10,5; 34,5; 18,0] | Left Anterior Cingulum | Left Anterior Cingulate Gyrus |
| 48 | 4,03 | 0,000 | 0,835 | 0,678 | 0,255 | [22,5; 60,0; 6,0] | Right Superior Frontal Gyrus | Right Superior Frontal Gyrus |
| 49 | 3,57 | 0,000 | 0,998 | 0,840 | 0,250 | [-21,0; -42,0; 58,5] | Left Superior Parietal Gyrus | Left Superior Parietal Lobule |
| 199 | 4,19 | 0,000 | 0,670 | 0,625 | 0,029 | [36,0; 30,0; 0,0] | Right Triangular Inferior Frontal Gyrus | Right Inferior Frontal Gyrus |
| 319 | 4,90 | 0,000 | 0,105 | 0,125 | 0,008 | [42,0; -7,5; 37,5] | Right Precentral Gyrus | Right Precentral Gyrus |

| Negative correlations found between GMV and HA | | | | | | | | |
|---|---|---|---|---|---|---|---|---|
| **Model = M2** | | | | | | | | |
| **Smoothing filter: FWHM=8mm** | | | | | | | | |
| | **peak** | **peak** | **peak** | **peak** | **cluster** | **Talairach** | **Anatomical labels** | |
| **Ke** | **T** | **p unc** | **p FWE** | **p FDR** | **p clus** | **x,y,z {mm}** | **AAL atlas** | **TD atlas** |
| 1 | 3,22 | 0,001 | 1,000 | 0,926 | 0,902 | [9,0; -82,5; -12,0] | Right Crus 1 Cerebelum | Not in atlas |
| 5 | 3,40 | 0,001 | 1,000 | 0,805 | 0,739 | [16,5; -76,5; -6,0] | Right Lingual Gyrus | Right Lingual Gyrus |
| 8 | 3,59 | 0,000 | 0,998 | 0,805 | 0,661 | [-30,0; 43,5; 4,5] | Left Middle Frontal Gyrus | Left Sub-Gyral |
| 8 | 3,35 | 0,001 | 1,000 | 0,812 | 0,661 | [-16,5; -28,5; -27,0] | Not in atlas | Not in atlas |
| 11 | 3,52 | 0,000 | 0,999 | 0,805 | 0,600 | [66,0; 13,5; 13,5] | Right Precentral Gyrus | Not in atlas |
| 11 | 3,29 | 0,001 | 1,000 | 0,857 | 0,600 | [-18,0; -82,5; -42,0] | Not in atlas | Not in atlas |
| 23 | 3,40 | 0,001 | 1,000 | 0,805 | 0,433 | [-45,0; 18,0; -12,0] | Left Superior Temporal Pole | Left Superior Temporal Gyrus |
| 30 | 3,80 | 0,000 | 0,968 | 0,701 | 0,368 | [63,0; -9,0; -24,0] | Right Inferior Temporal Gyrus | Not in atlas |
| 52 | 3,77 | 0,000 | 0,976 | 0,701 | 0,237 | [-12,0; -15,0; 39,0] | Left Middle Cingulum | Left Cingulate Gyrus |
| 73 | 3,96 | 0,000 | 0,888 | 0,701 | 0,164 | [48,0; 9,0; -16,5] | Right Superior Temporal Pole | Right Superior Temporal Gyrus |
| 117 | 3,90 | 0,000 | 0,927 | 0,701 | 0,084 | [-34,5; 9,0; -24,0] | Left Superior Temporal Pole | Left Superior Temporal Gyrus |

| Positive correlations found between GMV and HA | | | | | | | | |
|---|---|---|---|---|---|---|---|---|
| **Model = M3** | | | | | | | | |
| **Smoothing filter: FWHM=8mm** | | | | | | | | |
| | **peak** | **peak** | **peak** | **peak** | **cluster** | **Talairach** | **Anatomical labels** | |
| **Ke** | **T** | ***p* unc** | ***p* FWE** | ***p* FDR** | ***p* clus** | **x,y,z {mm}** | **AAL atlas** | **TD atlas** |
| 1 | 3,25 | 0,001 | 1,000 | 0,912 | 0,902 | [9,0; -37,5; 30,0] | Right Posterior Cingulum | Right Cingulate Gyrus |
| 1 | 3,25 | 0,001 | 1,000 | 0,912 | 0,902 | [-7,5; -37,5; 30,0] | Left Posterior Cingulum | Left Cingulate Gyrus |
| 1 | 3,19 | 0,001 | 1,000 | 0,983 | 0,902 | [4,5; -27,0; 33,0] | Right Middle Cingulum | Right Cingulate Gyrus |
| 4 | 3,28 | 0,001 | 1,000 | 0,912 | 0,770 | [25,5; 67,5; -9,0] | Not in atlas | Not in atlas |
| 13 | 3,32 | 0,001 | 1,000 | 0,899 | 0,564 | [16,5; 15,0; 45,0] | Right Superior Frontal Gyrus | Right Medial Frontal Gyrus |
| 17 | 3,67 | 0,000 | 0,992 | 0,709 | 0,504 | [36,0; 28,5; -1,5] | Right Insula | Right Sub-Gyral |
| 24 | 3,59 | 0,000 | 0,998 | 0,720 | 0,422 | [-42,0; -7,5; 33,0] | Left Postcentral Gyrus | Left Precentral Gyrus |
| 25 | 3,52 | 0,000 | 0,999 | 0,720 | 0,412 | [-63,0; -13,5; 16,5] | Left Postcentral Gyrus | Left Postcentral Gyrus |
| 27 | 3,54 | 0,000 | 0,999 | 0,720 | 0,394 | [45,0; 33,0; -9,0] | Right Orbital Inferior Frontal Gyrus | Right Inferior Frontal Gyrus |
| 29 | 3,59 | 0,000 | 0,998 | 0,720 | 0,376 | [16,5; 34,5; -12,0] | Not in atlas | Right Sub-Gyral |
| 38 | 3,81 | 0,000 | 0,964 | 0,544 | 0,310 | [-30,0; 43,5; 27,0] | Left Middle Frontal Gyrus | Left Superior Frontal Gyrus |
| 42 | 4,17 | 0,000 | 0,698 | 0,392 | 0,286 | [-39,0; 42,0; -9,0] | Left Orbital Middle Frontal Gyrus | Left Middle Frontal Gyrus |
| 45 | 4,07 | 0,000 | 0,795 | 0,415 | 0,270 | [22,5; 58,5; 4,5] | Right Superior Frontal Gyrus | Right Superior Frontal Gyrus |
| 64 | 3,84 | 0,000 | 0,954 | 0,544 | 0,191 | [54,0; 12,0; 27,0] | Right Opercular Inferior Frontal Gyrus | Right Inferior Frontal Gyrus |
| 103 | 4,31 | 0,000 | 0,536 | 0,336 | 0,103 | [45,0; -6,0; -24,0] | Right Middle Temporal Gyrus | Right Fusiform Gyrus |
| 103 | 3,91 | 0,000 | 0,919 | 0,544 | 0,103 | [-21,0; -42,0; 58,5] | Left Superior Parietal Gyrus | Left Superior Parietal Gyrus |
| 178 | 5,13 | 0,000 | 0,049 | 0,041 | 0,038 | [-10,5; 34,5; 18,0] | Left Anterior Cingulum | Left Anterior Cingulate Gyrus |
| 364 | 5,07 | 0,000 | 0,060 | 0,041 | 0,005 | [42,0; -6,0; 36,0] | Right Precentral Gyrus | Right Precentral Gyrus |

| Negative correlations found between GMV and HA | | | | | | | | |
|---|---|---|---|---|---|---|---|---|
| **Model = M3** | | | | | | | | |
| **Smoothing filter: FWHM=8mm** | | | | | | | | |
| | **peak** | **peak** | **peak** | **peak** | **cluster** | **Talairach** | **Anatomical labels** | |
| **Ke** | **T** | ***p* unc** | ***p* FWE** | ***p* FDR** | ***p* clus** | **x,y,z {mm}** | **AAL atlas** | **TD atlas** |
| 1 | 3,21 | 0,001 | 1,000 | 0,934 | 0,902 | [10,5; -69,0; 63,0] | Not in atlas | Right Precuneus |
| 3 | 3,24 | 0,001 | 1,000 | 0,934 | 0,806 | [22,5; -12,0; -7,5] | Not in atlas | Right Extra-Nuclear |
| 3 | 3,24 | 0,001 | 1,000 | 0,934 | 0,806 | [51,0 ; 24,0; -15,0] | Right Orbital Inferior Frontal gyrus | Not in atlas |
| 3 | 3,24 | 0,001 | 1,000 | 0,934 | 0,806 | [-36,0; 10,5; -22,5] | Left Superior Temporal Pole | Left Superior Temporal Gyrus |
| 4 | 3,28 | 0,001 | 1,000 | 0,934 | 0,770 | [-34,5; -82,5; 25,5] | Left Middle Occipital Gyrus | Left Superior Occipital Gyrus |
| 4 | 3,27 | 0,001 | 1,000 | 0,934 | 0,770 | [-48,0; 19,5; -10,5] | Left Superior Temporal Pole | Left Superior Temporal Gyrus |
| 5 | 3,44 | 0,000 | 1,000 | 0,934 | 0,739 | [66,0; -39,0; 10,5] | Right Middle Temporal Gyrus | Right Superior Temporal Gyrus |
| 17 | 3,37 | 0,001 | 1,000 | 0,934 | 0,504 | [-6,0; -76,5; 40,5] | Left Cuneus | Left Precuneus |
| 25 | 3,62 | 0,000 | 0,996 | 0,934 | 0,412 | [48,0; 9,0; -16,5] | Right Superior Temporal Pole | Right Superior Temporal Gyrus |
| 29 | 3,60 | 0,000 | 0,997 | 0,934 | 0,376 | [15,0; -73,5; 46,5] | Right Precuneus | Right Precuneus |
| 38 | 3,51 | 0,000 | 0,999 | 0,934 | 0,310 | [60,0; 9,0; -24,0] | Right Middle Temporal Pole | Not in atlas |
| 54 | 3,57 | 0,000 | 0,998 | 0,934 | 0,228 | [63,0; -6,0; -25,5] | Right Middle Temporal Gyrus | Not in atlas |
| 284 | 4,66 | 0,000 | 0,226 | 0,240 | 0,011 | [-18,0; -82,5; -42,0] | Not in atlas | Not in atlas |

**Positive correlations found between GMV and HA**

**Model = M4**
**Smoothing filter: FWHM=8mm**

| Ke | peak T | peak *p* unc | peak *p* FWE | peak *p* FDR | cluster *p* clus | Talairach x,y,z {mm} | Anatomical labels: AAL atlas | Anatomical labels: TD atlas |
|---|---|---|---|---|---|---|---|---|
| 1 | 3,20 | 0,001 | 1,000 | 0,976 | 0,901 | [-34,5; -28,5; -1,5] | Not in atlas | Left Extra-Nuclear |
| 2 | 3,24 | 0,001 | 1,000 | 0,963 | 0,847 | [-1,5; -28,5; 28,5] | Not in atlas | Left Cingulate Gyrus |
| 2 | 3,22 | 0,001 | 1,000 | 0,971 | 0,847 | [-36; -27,0; -3.0] | Not in atlas | Left Caudate |
| 3 | 3,27 | 0,001 | 1,000 | 0,943 | 0,805 | [21,0; 58,5; 4,5] | Right Superior Frontal Gyrus | Right Superior Frontal Gyrus |
| 3 | 3,26 | 0,001 | 1,000 | 0,943 | 0,805 | [-10,5; -48,0; 39,0] | Left Precuneus | Left Precuneus |
| 6 | 3,50 | 0,000 | 1,000 | 0,856 | 0,709 | [-30,0; 12,0; 66,0] | Not in atlas | Not in atlas |
| 6 | 3,41 | 0,000 | 1,000 | 0,867 | 0,709 | [-63,0; -13,5; 15,0] | Left Postcentral Gyrus | Left Postcentral Gyrus |
| 6 | 3,34 | 0,001 | 1,000 | 0,881 | 0,709 | [9,0; -37,5; 30,0] | Right Posterior Cingulum | Right Cingulate Gyrus |
| 9 | 3,35 | 0,001 | 1,000 | 0,881 | 0,637 | [-18,0; -90,0; -4,5] | Left Inferior Occipital Gyrus | Left Lingual Gyrus |
| 10 | 3,65 | 0,000 | 0,995 | 0,856 | 0,616 | [-42,0; -7,5; -21,0] | Not in atlas | Left Sub-Gyral |
| 10 | 3,32 | 0,001 | 1,000 | 0,898 | 0,616 | [57,0; -57,0; -19,5] | Right Inferior Temporal Gyrus | Not in atlas |
| 11 | 3,42 | 0,000 | 1,000 | 0,867 | 0,597 | [-21,0; -36,0; 57,0] | Not in atlas | Left Sub-Gyral |
| 12 | 3,50 | 0,000 | 1,000 | 0,856 | 0,579 | [42,0; 21,0; 15,0] | Not in atlas | Right Sub-Gyral |
| 12 | 3,41 | 0,001 | 1,000 | 0,867 | 0,579 | [28,5; -4,5; -36,0] | Right Fusiform Gyrus | Right Uncus |
| 13 | 3,45 | 0,000 | 1,000 | 0,867 | 0,562 | [13,5; 37,5; 36,0] | Right Superior Frontal Gyrus | Right Superior Frontal Gyrus |
| 16 | 3,54 | 0,000 | 0,999 | 0,856 | 0,516 | [-16,5; 1,5; 60,0] | Left Supplementary Motor area | Left Middle Frontal Gyrus |
| 17 | 3,50 | 0,000 | 1,000 | 0,856 | 0,502 | [-43,5; -7,5; 34,5] | Left Postcentral Gyrus | Left Precentral Gyrus |
| 24 | 3,71 | 0,000 | 0,989 | 0,837 | 0,420 | [-10,5; 49,5; 28,5] | Left Medial Superior Frontal Gyrus | Left Superior Frontal Gyrus |
| 26 | 3,56 | 0,000 | 0,999 | 0,856 | 0,400 | [4,5; -27,0; 33,0] | Right Middle Cingulum | Right Cingulate Gyrus |
| 40 | 3,59 | 0,000 | 0,998 | 0,856 | 0,295 | [43,5; 33,0; -7,5] | Right Orbital Inferior Frontal Gyrus | Right Inferior Frontal Gyrus |
| 105 | 4,89 | 0,000 | 0,119 | 0,117 | 0,098 | [-39,0; 42,0; -9,0] | Left Orbital Middle Frontal Gyrus | Left Middle Frontal Gyrus |
| 110 | 4,09 | 0,000 | 0,794 | 0,419 | 0,091 | [43,5; -9,0; -19,5] | Not in atlas | Right Sub-Gyral |
| 151 | 4,73 | 0,000 | 0,194 | 0,129 | 0,052 | [-9,0; 34,5; 19,5] | Left Anterior Cingulum | Left Anterior Cingulate Gyrus |
| 189 | 4,94 | 0,000 | 0,101 | 0,117 | 0,032 | [16,5; 34,5; -12,0] | Not in atlas | Right Sub-Gyral |
| 200 | 4,58 | 0,000 | 0,294 | 0,129 | 0,028 | [48,0; 0,0; 24,0] | Right Precentral Gyrus | Right Inferior Frontal Gyrus |
| 279 | 4,18 | 0,000 | 0,707 | 0,380 | 0,012 | [40,5; -7,5; 36,0] | Not in atlas | Right Precentral Gyrus |

**Negative correlations found between GMV and HA**

**Model = M4**
**Smoothing filter: FWHM=8mm**

| Ke | peak T | peak *p* unc | peak *p* FWE | peak *p* FDR | cluster *p* clus | Talairach x,y,z {mm} | Anatomical labels: AAL atlas | Anatomical labels: TD atlas |
|---|---|---|---|---|---|---|---|---|
| 1 | 3,22 | 0,001 | 1,000 | 0,934 | 0,901 | [49,5; 24,0; -15,0] | Right Orbital Inferior Frontal Gyrus | Not in atlas |
| 4 | 3,35 | 0,001 | 1,000 | 0,875 | 0,769 | [-7,5; -78,0; 42,0] | Left Superior Occipital Gyrus | Left Precuneus |
| 99 | 4,01 | 0,000 | 0,865 | 0,409 | 0,107 | [63,0; -52,5; 13,5] | Right Middle Temporal Gyrus | Right Superior Temporal Gyrus |
| 129 | 3,91 | 0,000 | 0,929 | 0,409 | 0,070 | [-18,0; -84,0; -40,5] | Not in atlas | Not in atlas |

| Positive correlations found between GMV and HA | | | | | | | | |
|---|---|---|---|---|---|---|---|---|
| **Model = M1** | | | | | | | | |
| **Smoothing filter: FWHM=12mm** | | | | | | | | |
| **Ke** | **peak T** | **peak *p* unc** | **peak *p* FWE** | **peak *p* FDR** | **cluster *p* clus** | **Talairach x,y,z {mm}** | **Anatomical labels AAL atlas** | **TD atlas** |
| No correlations found | | | | | | | | |

| Negative correlations found between GMV and HA | | | | | | | | |
|---|---|---|---|---|---|---|---|---|
| **Model = M1** | | | | | | | | |
| **Smoothing filter: FWHM=12mm** | | | | | | | | |
| **Ke** | **peak T** | **peak *p* unc** | **peak *p* FWE** | **peak *p* FDR** | **cluster *p* clus** | **Talairach x,y,z {mm}** | **Anatomical labels AAL atlas** | **TD atlas** |
| No correlations found | | | | | | | | |

| Positive correlations found between GMV and HA | | | | | | | | |
|---|---|---|---|---|---|---|---|---|
| **Model = M2**<br>**Smoothing filter: FWHM=12mm** | | | | | | | | |
| **Ke** | **peak T** | **peak *p* unc** | **peak *p* FWE** | **peak *p* FDR** | **cluster *p* clus** | **Talairach x,y,z {mm}** | **Anatomical labels AAL atlas** | **TD atlas** |
| No correlations found | | | | | | | | |

| Negative correlations found between GMV and HA | | | | | | | | |
|---|---|---|---|---|---|---|---|---|
| **Model = M2**<br>**Smoothing filter: FWHM=12mm** | | | | | | | | |
| **Ke** | **peak T** | **peak *p* unc** | **peak *p* FWE** | **peak *p* FDR** | **cluster *p* clus** | **Talairach x,y,z {mm}** | **Anatomical labels AAL atlas** | **TD atlas** |
| No correlations found | | | | | | | | |

| Positive correlations found between GMV and HA | | | | | | | | |
|---|---|---|---|---|---|---|---|---|
| **Model = M3** | | | | | | | | |
| **Smoothing filter: FWHM=12mm** | | | | | | | | |
| | **peak** | **peak** | **peak** | **peak** | **cluster** | **Talairach** | **Anatomical labels** | |
| **Ke** | **T** | ***p* unc** | ***p* FWE** | ***p* FDR** | ***p* clus** | **x,y,z {mm}** | **AAL atlas** | **TD atlas** |
| 13 | 3,25 | 0,001 | 0,834 | 0,852 | 0,879 | [40,5; -9,0; 36,0] | Not in atlas | Right Precentral Gyrus |

| Negative correlations found between GMV and HA | | | | | | | | |
|---|---|---|---|---|---|---|---|---|
| **Model = M3** | | | | | | | | |
| **Smoothing filter: FWHM=12mm** | | | | | | | | |
| | **peak** | **peak** | **peak** | **peak** | **cluster** | **Talairach** | **Anatomical labels** | |
| **Ke** | **T** | ***p* unc** | ***p* FWE** | ***p* FDR** | ***p* clus** | **x,y,z {mm}** | **AAL atlas** | **TD atlas** |
| 94 | 3,42 | 0,000 | 0,698 | 0,568 | 0,617 | [63,0; -1,5; -28,5] | Not in atlas | Not in atlas |

| Positive correlations found between GMV and HA | | | | | | | | |
|---|---|---|---|---|---|---|---|---|
| **Model = M4** | | | | | | | | |
| **Smoothing filter: FWHM=12mm** | | | | | | | | |
| | **peak** | **peak** | **peak** | **peak** | **cluster** | **Talairach** | **Anatomical labels** | |
| **Ke** | **T** | ***p* unc** | ***p* FWE** | ***p* FDR** | ***p* clus** | **x,y,z {mm}** | **AAL atlas** | **TD atlas** |
| No correlations found | | | | | | | | |

| Positive correlations found between GMV and HA | | | | | | | | |
|---|---|---|---|---|---|---|---|---|
| **Model = M4** | | | | | | | | |
| **Smoothing filter: FWHM=12mm** | | | | | | | | |
| | **peak** | **peak** | **peak** | **peak** | **cluster** | **Talairach** | **Anatomical labels** | |
| **Ke** | **T** | ***p* unc** | ***p* FWE** | ***p* FDR** | ***p* clus** | **x,y,z {mm}** | **AAL atlas** | **TD atlas** |
| 56 | 3,40 | 0,001 | 0,727 | 0,616 | 0,711 | [15,0; -42,0; -10,5] | Right Cerebelum 4-5 | Right Culmen |
