# Supplementary material for: The Heterogeneity in Retrieved Relations between the Personality Trait ‘Harm Avoidance’ and Gray Matter Volumes Due to Variations in the VBM and ROI Labeling Processing Settings
Source: PLoS One. 2016 Apr 20;11(4):e0153865. doi: 10.1371/journal.pone.0153865 (PMC4838261; doi:10.1371/journal.pone.0153865)
Supplement: S2 File — None of the correlations survived multiple comparisons correction. (PDF) [file pone.0153865.s002.pdf]

Results ROI labeling analyses using BrainSuite

	Significance HA as predictor in the model															
	M1				M2				M3				M4			
	t	punc	pFDR	pBonf	t	punc	pFDR	pBonf	t	punc	pFDR	pBonf	t	punc	pFDR	pBonf
Right Superior Frontal Gyrus	0.34	0.738	0.989	1.000	0.32	0.748	0.958	1.000	0.45	0.652	0.925	1.000	-0.07	0.948	0.998	1.000
Left Superior Frontal Gyrus	0.62	0.535	0.963	1.000	0.76	0.447	0.958	1.000	0.60	0.552	0.904	1.000	-0.02	0.987	0.998	1.000
Right Middle Frontal Gyrus	0.18	0.859	0.989	1.000	0.18	0.860	0.958	1.000	-0.28	0.783	0.925	1.000	-0.22	0.828	0.985	1.000
Left Middle Frontal Gyrus	0.69	0.495	0.963	1.000	0.63	0.530	0.958	1.000	0.59	0.555	0.904	1.000	0.64	0.522	0.972	1.000
Right Pars Opercularis	0.50	0.619	0.989	1.000	0.27	0.791	0.958	1.000	0.32	0.750	0.925	1.000	-0.09	0.930	0.998	1.000
Left Pars Opercularis	1.60	0.114	0.925	1.000	1.63	0.107	0.936	1.000	1.05	0.295	0.904	1.000	1.16	0.251	0.884	1.000
Right Pars Triangularis	-0.46	0.650	0.989	1.000	-0.32	0.751	0.958	1.000	-0.23	0.823	0.928	1.000	0.41	0.683	0.985	1.000
Left Pars Triangularis	0.99	0.325	0.925	1.000	0.77	0.443	0.958	1.000	0.38	0.708	0.925	1.000	0.24	0.815	0.985	1.000
Right Pars Orbitalis	-0.27	0.788	0.989	1.000	-0.33	0.740	0.958	1.000	-0.61	0.546	0.904	1.000	-0.29	0.770	0.985	1.000
Left Pars Orbitalis	0.75	0.455	0.963	1.000	0.85	0.396	0.958	1.000	0.92	0.359	0.904	1.000	0.13	0.901	0.998	1.000
Right Precentral Gyrus	0.18	0.857	0.989	1.000	0.16	0.871	0.958	1.000	0.07	0.945	0.967	1.000	0.20	0.839	0.985	1.000
Left Precentral Gyrus	0.73	0.470	0.963	1.000	0.62	0.534	0.958	1.000	0.86	0.395	0.904	1.000	0.69	0.494	0.972	1.000
Right Transvers Frontal Gyrus	-0.18	0.857	0.989	1.000	-0.72	0.943	0.965	1.000	-0.39	0.696	0.925	1.000	-0.16	0.873	0.985	1.000
Left Transvers Frontal Gyrus	0.37	0.710	0.989	1.000	0.34	0.738	0.958	1.000	0.53	0.596	0.904	1.000	0.00	0.997	0.998	1.000
Right Gyrus Rectus	-0.97	0.334	0.925	1.000	-0.74	0.464	0.958	1.000	-1.15	0.254	0.904	1.000	-1.48	0.142	0.781	1.000
Left Gyrus Rectus	-1.07	0.290	0.925	1.000	-0.96	0.340	0.958	1.000	-1.03	0.304	0.904	1.000	-0.96	0.340	0.931	1.000
Right Middle Orbitofrontal Gyrus	0.25	0.800	0.989	1.000	0.14	0.889	0.965	1.000	0.55	0.586	0.904	1.000	0.08	0.941	0.998	1.000
Left Middle Orbitofrontal Gyrus	0.05	0.957	0.989	1.000	0.04	0.968	0.968	1.000	-0.19	0.854	0.928	1.000	-0.30	0.763	0.985	1.000
Right Anterior Orbitofrontal Gyrus	0.01	0.989	0.989	1.000	0.06	0.954	0.965	1.000	-0.54	0.593	0.904	1.000	-0.65	0.515	0.972	1.000
Left Anterior Orbitofrontal Gyrus	0.12	0.905	0.989	1.000	0.21	0.836	0.958	1.000	-0.03	0.978	0.989	1.000	-0.17	0.866	0.985	1.000
Right Posterior Orbitofrontal Gyrus	-0.20	0.844	0.989	1.000	-0.35	0.729	0.958	1.000	-0.50	0.617	0.920	1.000	0.38	0.705	0.985	1.000
Left Posterior Orbitofrontal Gyrus	-0.15	0.885	0.989	1.000	-0.33	0.741	0.958	1.000	-0.20	0.845	0.928	1.000	-0.38	0.703	0.985	1.000
Right Lateral Orbitofrontal Gyrus	0.75	0.456	0.963	1.000	0.65	0.519	0.958	1.000	0.95	0.345	0.904	1.000	0.58	0.564	0.976	1.000
Left lateral Orbitofrontal Gyrus	-0.34	0.736	0.989	1.000	-0.49	0.624	0.958	1.000	-0.70	0.488	0.904	1.000	-0.27	0.791	0.985	1.000
Right Paracentral Lobule	0.92	0.360	0.925	1.000	0.85	0.399	0.958	1.000	1.37	0.174	0.904	1.000	0.99	0.328	0.931	1.000
Left Paracentral Lobule	0.97	0.336	0.925	1.000	0.92	0.362	0.958	1.000	0.82	0.414	0.904	1.000	0.54	0.594	0.985	1.000
Right Cingulate Gyrus	0.45	0.655	0.989	1.000	0.35	0.728	0.958	1.000	0.94	0.348	0.904	1.000	0.69	0.491	0.972	1.000
Left Cingulate Gyrus	-1.30	0.197	0.925	1.000	-1.34	0.185	0.936	1.000	-0.87	0.387	0.904	1.000	-1.31	0.194	0.813	1.000
Right Subcallosal Gyrus	-0.27	0.786	0.989	1.000	-0.21	0.831	0.958	1.000	-0.11	0.910	0.953	1.000	-0.22	0.825	0.985	1.000
Left Subcallosal Gyrus	0.59	0.557	0.963	1.000	0.48	0.631	0.958	1.000	1.23	0.224	0.904	1.000	0.78	0.436	0.972	1.000
Right Postcentral Gyrus	1.44	0.153	0.925	1.000	1.34	0.184	0.936	1.000	1.77	0.080	0.904	1.000	2.14	0.036	0.638	1.000
Left Postcentral Gyrus	-0.38	0.704	0.989	1.000	-0.36	0.718	0.958	1.000	1.14	0.258	0.904	1.000	0.74	0.464	0.972	1.000
Right Supramarginal Gyrus	1.69	0.095	0.925	1.000	1.61	0.111	0.936	1.000	1.81	0.073	0.904	1.000	1.78	0.079	0.698	1.000
Left Supramarginal Gyrus	0.19	0.853	0.989	1.000	0.07	0.941	0.965	1.000	0.37	9.710	1.000	1.000	0.64	0.524	0.972	1.000
Right Angular Gyrus	0.22	0.823	0.989	1.000	0.25	0.803	0.958	1.000	0.35	0.726	0.925	1.000	1.64	0.104	0.698	1.000
Left Angular Gyrus	1.43	0.156	0.925	1.000	1.49	0.139	0.936	1.000	1.19	0.236	0.904	1.000	1.17	0.247	0.884	1.000
Right Superior Parietal Gyrus	0.75	0.454	0.963	1.000	0.73	0.467	0.958	1.000	0.68	0.496	0.904	1.000	1.00	0.318	0.931	1.000
Left Superior Parietal Gyrus	1.62	0.109	0.925	1.000	1.64	0.105	0.936	1.000	1.30	0.198	0.904	1.000	0.74	0.463	0.972	1.000
Right Precuneus	-0.76	0.451	0.963	1.000	-0.90	0.371	0.958	1.000	-0.70	0.488	0.904	1.000	-0.37	0.710	0.985	1.000
Left Precuneus	-0.90	0.368	0.925	1.000	-0.74	0.461	0.958	1.000	-0.84	0.405	0.904	1.000	-0.66	0.509	0.972	1.000
Right Temporal Pole	0.81	0.421	0.963	1.000	0.92	0.359	0.958	1.000	0.69	0.493	0.904	1.000	1.39	0.169	0.811	1.000
Left Temporal Pole	0.45	0.654	0.989	1.000	0.53	0.599	0.958	1.000	1.06	0.295	0.904	1.000	1.00	0.322	0.931	1.000
Right Superior Temporal Gyrus	1.11	0.269	0.925	1.000	1.07	0.289	0.958	1.000	0.72	0.473	0.904	1.000	0.94	0.349	0.931	1.000
Left Superior Temporal Gyrus	-0.07	0.948	0.989	1.000	-0.21	0.838	0.958	1.000	0.94	0.350	0.904	1.000	0.51	0.611	0.985	1.000
Right Transverse Temporal Gyrus	-0.62	0.536	0.963	1.000	-0.63	0.531	0.958	1.000	-0.58	0.566	0.904	1.000	-0.34	0.734	0.985	1.000
Left Transverse Temporal Gyrus	-0.72	0.473	0.963	1.000	-0.75	0.458	0.958	1.000	-0.34	0.736	0.925	1.000	0.05	0.961	0.998	1.000
Right Middle Temporal Gyrus	1.53	0.129	0.925	1.000	1.69	0.095	0.936	1.000	0.95	0.346	0.904	1.000	1.71	0.090	0.698	1.000
Left Middle Temporal Gyrus	0.06	0.949	0.989	1.000	0.07	0.945	0.965	1.000	-0.08	0.939	0.967	1.000	0.00	0.997	0.998	1.000
Right Inferior Temporal Gyrus	0.43	0.671	0.989	1.000	0.48	0.631	0.958	1.000	0.53	0.595	0.904	1.000	0.63	0.530	0.972	1.000
Left Inferior Temporal Gyrus	-0.62	0.540	0.963	1.000	-0.54	0.589	0.958	1.000	-0.64	0.524	0.904	1.000	-0.67	0.504	0.972	1.000
Right Fusiforme Gyrus	2.11	0.038	0.925	1.000	2.01	0.047	0.936	1.000	1.82	0.073	0.904	1.000	2.47	0.016	0.638	1.000
Left Fusiforme Gyrus	-1.51	0.135	0.925	1.000	-1.52	0.132	0.936	1.000	-2.23	0.028	0.821	1.000	-2.12	0.037	0.638	1.000
Right Parahippocampal Gyrus	-1.62	0.109	0.925	1.000	-1.70	0.094	0.936	1.000	-1.61	0.111	0.904	1.000	-2.19	0.031	0.638	1.000
Left Parahippocampal Gyrus	1.07	0.288	0.925	1.000	1.11	0.269	0.958	1.000	1.02	0.309	0.904	1.000	0.72	0.473	0.972	1.000
Right Hippocampus	-0.76	0.450	0.963	1.000	-0.62	0.535	0.958	1.000	-0.30	0.767	0.925	1.000	-0.33	0.745	0.985	1.000
Left Hippocampus	-0.95	0.345	0.925	1.000	-0.89	0.377	0.958	1.000	-0.34	0.734	0.925	1.000	0.28	0.778	0.985	1.000
Right Amygdala	1.45	0.152	0.925	1.000	1.46	0.147	0.936	1.000	2.85	0.006	0.528	0.528	2.63	0.010	0.638	0.880
Left Amygdala	-0.98	0.329	0.925	1.000	-0.96	0.338	0.958	1.000	-0.15	0.884	0.937	1.000	-0.32	0.750	0.985	1.000
Right Superior Occipital Gyrus	1.34	0.183	0.925	1.000	1.34	0.185	0.936	1.000	1.20	0.235	0.904	1.000	1.03	0.305	0.931	1.000
Left Superior Occipital Gyrus	1.62	0.109	0.925	1.000	1.73	0.087	0.936	1.000	1.48	0.142	0.904	1.000	0.44	0.660	0.985	1.000
Right Middle Occipital Gyrus	1.09	0.278	0.925	1.000	1.01	0.317	0.958	1.000	1.35	0.181	0.904	1.000	1.37	0.175	0.811	1.000
Left Middle Occipital Gyrus	0.93	0.353	0.925	1.000	0.84	0.401	0.958	1.000	0.66	0.512	0.904	1.000	0.00	0.998	0.998	1.000
Right Inferior Occipital Gyrus	0.03	0.975	0.989	1.000	0.08	0.941	0.965	1.000	0.17	0.867	0.930	1.000	-0.35	0.724	0.985	1.000
Left Inferior Occipital Gyrus	-1.06	0.294	0.925	1.000	-1.21	0.231	0.958	1.000	-0.82	0.415	0.904	1.000	0.34	0.735	0.985	1.000
Right Lingual Gyrus	-1.58	0.118	0.925	1.000	-1.94	0.056	0.936	1.000	-0.81	0.421	0.904	1.000	-1.21	0.229	0.876	1.000
Left Lingual Gyrus	-0.59	0.558	0.963	1.000	-0.70	0.488	0.958	1.000	-0.18	0.854	0.928	1.000	-0.71	0.481	0.972	1.000
Right Cuneus	-1.00	0.320	0.925	1.000	-0.93	0.356	0.958	1.000	-0.75	0.458	0.904	1.000	0.56	0.575	0.976	1.000
Left Cuneus	0.02	0.985	0.989	1.000	0.12	0.908	0.965	1.000	-0.31	0.756	0.925	1.000	1.22	0.226	0.876	1.000
Right Insula	-0.03	0.975	0.989	1.000	-0.37	0.713	0.958	1.000	-0.20	0.841	0.928	1.000	0.19	0.851	0.985	1.000
Left Insula	-1.31	0.194	0.925	1.000	-1.57	0.120	0.936	1.000	-2.03	0.046	0.904	1.000	-1.45	0.152	0.787	1.000
Right Caudate Nucleus	-0.41	0.687	0.989	1.000	-0.29	0.773	0.958	1.000	-0.36	0.723	0.925	1.000	0.74	0.464	0.972	1.000
Left Caudate Nucleus	-0.40	0.693	0.989	1.000	-0.47	0.637	0.958	1.000	0.56	0.578	0.904	1.000	1.48	0.142	0.781	1.000
Right Putamen	-0.99	0.324	0.925	1.000	-1.30	0.197	0.936	1.000	-0.92	0.362	0.904	1.000	-1.64	0.104	0.698	1.000
Left Putamen	-0.04	0.973	0.989	1.000	-0.25	0.804	0.958	1.000	-1.21	0.229	0.904	1.000	-1.99	0.050	0.638	1.000
Right Globus Pallidus	-0.28	0.782	0.989	1.000	-0.36	0.724	0.958	1.000	-0.44	0.664	0.925	1.000	-0.56	0.577	0.976	1.000
Left Globus Pallidus	-1.33	0.188	0.925	1.000	-1.29	0.202	0.936	1.000	-2.25	0.027	0.821	1.000	-1.94	0.056	0.638	1.000
Right Nucleus Accumbens	0.49	0.629	0.989	1.000	0.62	0.538	0.958	1.000	0.61	0.547	0.904	1.000	1.61	0.111	0.698	1.000
Left Nucleus Accumbens	2.50	0.014	0.925	1.000	2.57	0.012	0.936	1.000	1.70	0.094	0.904	1.000	1.66	0.101	0.698	1.000
Right Thalamus	-0.60	0.553	0.963	1.000	-0.90	0.370	0.958	1.000	-0.27	0.788	0.925	1.000	-0.24	0.808	0.985	1.000
Left Thalamus	-0.13	0.896	0.989	1.000	-0.35	0.730	0.958	1.000	0.44	0.660	0.925	1.000	0.85	0.399	0.972	1.000
Right Superior colliculus	0.93	0.357	0.925	1.000	0.77	0.445	0.958	1.000	0.24	0.808	0.928	1.000	0.19	0.850	0.985	1.000
Left Superior colliculus	0.65	0.521	0.963	1.000	0.59	0.559	0.958	1.000	0.28	0.778	0.925	1.000	-0.06	0.951	0.998	1.000
Right Inferior colliculus	0.37	0.710	0.989	1.000	0.19	0.849	0.958	1.000	-0.29	0.775	0.925	1.000	-0.48	0.635	0.985	1.000
Left Inferior colliculus	-0.28	0.779	0.989	1.000	-0.25	0.807	0.958	1.000	-2.09	0.278	0.904	1.000	-2.33	0.187	0.813	1.000
Right mamillary body	-0.17	0.863	0.989	1.000	-0.23	0.823	0.958	1.000	-1.40	0.165	0.904	1.000	-1.02	0.313	0.931	1.000
Left mamillary body	-1.31	0.192	0.925	1.000	-1.35	0.180	0.936	1.000	-1.64	0.105	0.904	1.000	-0.57	0.569	0.976	1.000
Brainstem	0.10	0.918	0.989	1.000	0.34	0.735	0.958	1.000	-0.93	0.356	0.904	1.000	-1.92	0.058	0.638	1.000
Cerebellum	-0.99	0.324	0.925	1.000	-0.92	0.362	0.958	1.000	-2.07	0.290	0.904	1.000	-1.12	0.265	0.897	1.000
