# Supplementary material for: The Heterogeneity in Retrieved Relations between the Personality Trait ‘Harm Avoidance’ and Gray Matter Volumes Due to Variations in the VBM and ROI Labeling Processing Settings
Source: PLoS One. 2016 Apr 20;11(4):e0153865. doi: 10.1371/journal.pone.0153865 (PMC4838261; doi:10.1371/journal.pone.0153865)
Supplement: S3 File — None of the correlations survived multiple comparisons correction. (PDF) [file pone.0153865.s003.pdf]

| | | Significance HA as predictor in the model | | | | | | | | | | | | | | | |
|---|---|---|---|---|---|---|---|---|---|---|---|---|---|---|---|---|---|
| | | M1 | | | | M2 | | | | M3 | | | | M4 | | | |
| | | t | punc | pFDR | pBonf | t | punc | pFDR | pBonf | t | punc | pFDR | pBonf | t | punc | pFDR | pBonf |
| Atlas: aseg | Left Thalamus Proper | 0.17 | 0.866 | 0.989 | 1.000 | 0.21 | 0.837 | 0.937 | 1.000 | -0.24 | 0.813 | 0.961 | 1.000 | -0.75 | 0.033 | 0.599 | 1.000 |
| | Left Caudate | -0.14 | 0.886 | 0.989 | 1.000 | -0.35 | 0.725 | 0.937 | 1.000 | -0.19 | 0.852 | 0.973 | 1.000 | 0.28 | 0.784 | 0.973 | 1.000 |
| | Left Putamen | -0.30 | 0.763 | 0.989 | 1.000 | -0.50 | 0.618 | 0.907 | 1.000 | -0.93 | 0.354 | 0.926 | 1.000 | -0.81 | 0.421 | 0.973 | 1.000 |
| | Left Pallidum | -0.69 | 0.491 | 0.907 | 1.000 | -1.04 | 0.300 | 0.896 | 1.000 | -0.53 | 0.600 | 0.926 | 1.000 | -0.16 | 0.874 | 0.973 | 1.000 |
| | Brainstem | 0.51 | 0.610 | 0.977 | 1.000 | 0.58 | 0.565 | 0.896 | 1.000 | -0.17 | 0.866 | 0.973 | 1.000 | -0.75 | 0.457 | 0.973 | 1.000 |
| | Left Hippocampus | -0.83 | 0.407 | 0.907 | 1.000 | -0.65 | 0.519 | 0.896 | 1.000 | -0.43 | 0.671 | 0.926 | 1.000 | -0.62 | 0.539 | 0.973 | 1.000 |
| | Left Amygdala | 0.20 | 0.845 | 0.989 | 1.000 | 0.24 | 0.810 | 0.937 | 1.000 | -0.13 | 0.901 | 0.973 | 1.000 | 0.07 | 0.949 | 0.990 | 1.000 |
| | Left Accumbens Area | -1.85 | 0.067 | 0.666 | 1.000 | -1.88 | 0.064 | 0.629 | 1.000 | -0.98 | 0.331 | 0.926 | 1.000 | -0.67 | 0.502 | 0.973 | 1.000 |
| | Right Thalamus Proper | 0.18 | 0.857 | 0.989 | 1.000 | 0.29 | 0.770 | 0.937 | 1.000 | -0.69 | 0.495 | 0.926 | 1.000 | -1.17 | 0.247 | 0.928 | 1.000 |
| | Right Caudate | -0.30 | 0.763 | 0.989 | 1.000 | -0.48 | 0.636 | 0.907 | 1.000 | -0.33 | 0.746 | 0.961 | 1.000 | 0.53 | 0.597 | 0.973 | 1.000 |
| | Right Putamen | -0.50 | 0.618 | 0.977 | 1.000 | -0.66 | 0.508 | 0.896 | 1.000 | -0.55 | 0.581 | 0.926 | 1.000 | -0.30 | 0.764 | 0.973 | 1.000 |
| | Right Pallidum | -1.82 | 0.072 | 0.676 | 1.000 | -2.05 | 0.043 | 0.581 | 1.000 | -2.19 | 0.031 | 0.416 | 1.000 | -1.92 | 0.058 | 0.665 | 1.000 |
| | Right Hippocampus | -0.81 | 0.419 | 0.907 | 1.000 | -0.62 | 0.536 | 0.896 | 1.000 | -0.18 | 0.857 | 0.973 | 1.000 | 0.32 | 0.751 | 0.973 | 1.000 |
| | Right Amygdala | -0.02 | 0.983 | 0.995 | 1.000 | -0.10 | 0.919 | 0.964 | 1.000 | 0.24 | 0.813 | 0.961 | 1.000 | 1.35 | 0.179 | 0.864 | 1.000 |
| | Right Accumbens Area | -1.72 | 0.089 | 0.716 | 1.000 | -1.53 | 0.131 | 0.896 | 1.000 | -1.01 | 0.315 | 0.926 | 1.000 | -0.40 | 0.693 | 0.973 | 1.000 |
| Atlas: Desikan-Killiany | Left Caudal Anterior Cingulate | -1.59 | 0.116 | 0.784 | 1.000 | -1.47 | 0.144 | 0.896 | 1.000 | -1.61 | 0.112 | 0.789 | 1.000 | -1.88 | 0.063 | 0.665 | 1.000 |
| | Left Caudal Middle Frontal | 0.04 | 0.973 | 0.991 | 1.000 | -0.13 | 0.898 | 0.957 | 1.000 | 0.76 | 0.451 | 0.926 | 1.000 | 0.24 | 0.811 | 0.973 | 1.000 |
| | Left Cuneus | -0.33 | 0.740 | 0.989 | 1.000 | -0.49 | 0.622 | 0.907 | 1.000 | -1.12 | 0.265 | 0.926 | 1.000 | -1.02 | 0.310 | 0.950 | 1.000 |
| | Left Entorhinal | 0.99 | 0.323 | 0.907 | 1.000 | 0.95 | 0.343 | 0.896 | 1.000 | 0.58 | 0.561 | 0.926 | 1.000 | 1.84 | 0.069 | 0.686 | 1.000 |
| | Left Fusiform | 0.72 | 0.473 | 0.907 | 1.000 | 0.88 | 0.383 | 0.896 | 1.000 | 0.71 | 0.481 | 0.926 | 1.000 | 0.62 | 0.539 | 0.973 | 1.000 |
| | Left Inferior Parietal | 0.05 | 0.962 | 0.989 | 1.000 | 0.32 | 0.751 | 0.937 | 1.000 | 0.12 | 0.905 | 0.973 | 1.000 | 0.60 | 0.548 | 0.973 | 1.000 |
| | Left Inferior Temporal | 0.68 | 0.497 | 0.907 | 1.000 | 0.84 | 0.402 | 0.896 | 1.000 | 0.55 | 0.582 | 0.926 | 1.000 | 0.82 | 0.418 | 0.973 | 1.000 |
| | Left Isthmus Cingulate | 0.04 | 0.966 | 0.989 | 1.000 | 0.07 | 0.941 | 0.976 | 1.000 | -0.11 | 0.909 | 0.973 | 1.000 | 0.49 | 0.629 | 0.973 | 1.000 |
| | Left Lateral Occipital | -0.96 | 0.342 | 0.907 | 1.000 | -0.70 | 0.487 | 0.896 | 1.000 | -1.28 | 0.206 | 0.926 | 1.000 | -0.64 | 0.522 | 0.973 | 1.000 |
| | Left Lateral Orbitofrontal | -0.61 | 0.545 | 0.940 | 1.000 | -0.73 | 0.468 | 0.896 | 1.000 | 0.46 | 0.648 | 0.926 | 1.000 | 0.82 | 0.413 | 0.973 | 1.000 |
| | Left Lingual | -0.65 | 0.515 | 0.907 | 1.000 | -0.58 | 0.563 | 0.896 | 1.000 | -0.45 | 0.657 | 0.926 | 1.000 | 0.24 | 0.812 | 0.973 | 1.000 |
| | Left Medial Orbitofrontal | 0.20 | 0.846 | 0.989 | 1.000 | 0.39 | 0.698 | 0.937 | 1.000 | 0.98 | 0.330 | 0.926 | 1.000 | 0.06 | 0.955 | 0.990 | 1.000 |
| | Left Middle Temporal | 0.28 | 0.783 | 0.989 | 1.000 | 0.46 | 0.644 | 0.907 | 1.000 | -0.30 | 0.763 | 0.961 | 1.000 | -0.88 | 0.379 | 0.973 | 1.000 |
| | Left Parahippocampal | -0.78 | 0.439 | 0.907 | 1.000 | -0.65 | 0.518 | 0.896 | 1.000 | 0.63 | 0.529 | 0.926 | 1.000 | 1.00 | 0.321 | 0.950 | 1.000 |
| | Left Paracentral | -2.00 | 0.049 | 0.631 | 1.000 | -2.05 | 0.430 | 0.896 | 1.000 | -1.33 | 0.187 | 0.926 | 1.000 | -1.49 | 0.140 | 0.816 | 1.000 |
| | Left Pars Opercularis | 0.72 | 0.473 | 0.907 | 1.000 | 0.73 | 0.471 | 0.896 | 1.000 | 0.48 | 0.635 | 0.926 | 1.000 | 1.18 | 0.241 | 0.926 | 1.000 |
| | Left Pars Orbitalis | -0.82 | 0.413 | 0.907 | 1.000 | -1.00 | 0.321 | 0.896 | 1.000 | -1.42 | 0.159 | 0.896 | 1.000 | -1.51 | 0.136 | 0.816 | 1.000 |
| | Left Pars Triangularis | 3.11 | 0.002 | 0.084 | 0.338 | 2.90 | 0.005 | 0.169 | 0.845 | 1.91 | 0.059 | 0.499 | 1.000 | 1.65 | 0.102 | 0.739 | 1.000 |
| | Left Pericalcarine | 0.26 | 0.792 | 0.989 | 1.000 | 0.22 | 0.829 | 0.937 | 1.000 | 0.25 | 0.806 | 0.961 | 1.000 | 0.38 | 0.705 | 0.973 | 1.000 |
| | Left Postcentral | -1.12 | 0.264 | 0.907 | 1.000 | -0.93 | 0.357 | 0.896 | 1.000 | -0.51 | 0.612 | 0.926 | 1.000 | -0.33 | 0.741 | 0.973 | 1.000 |
| | Left Posterior Cingulate | -1.54 | 0.127 | 0.795 | 1.000 | -1.29 | 0.201 | 0.896 | 1.000 | -1.08 | 0.281 | 0.926 | 1.000 | -0.34 | 0.733 | 0.973 | 1.000 |
| | Left Precentral | -0.93 | 0.358 | 0.907 | 1.000 | -0.90 | 0.373 | 0.896 | 1.000 | -1.02 | 0.313 | 0.926 | 1.000 | -0.77 | 0.445 | 0.973 | 1.000 |
| | Left Precuneus | 0.84 | 0.403 | 0.907 | 1.000 | 0.70 | 0.485 | 0.896 | 1.000 | 0.26 | 0.795 | 0.961 | 1.000 | 0.54 | 0.591 | 0.973 | 1.000 |
| | Left Rostral Anterior Cingulate | -2.27 | 0.026 | 0.399 | 1.000 | -2.10 | 0.039 | 0.581 | 1.000 | -2.61 | 0.011 | 0.292 | 1.000 | -2.26 | 0.027 | 0.599 | 1.000 |
| | Left Rostral Middle Frontal | 2.00 | 0.049 | 0.631 | 1.000 | 1.96 | 0.053 | 0.581 | 1.000 | 2.72 | 0.008 | 0.292 | 1.000 | 2.75 | 0.007 | 0.592 | 1.000 |
| | Left Superior Frontal | -0.29 | 0.771 | 0.989 | 1.000 | -0.61 | 0.544 | 0.896 | 1.000 | 1.34 | 0.185 | 0.926 | 1.000 | 1.20 | 0.235 | 0.924 | 1.000 |
| | Left Superior Parietal | 0.41 | 0.684 | 0.989 | 1.000 | 0.39 | 0.701 | 0.937 | 1.000 | -0.67 | 0.507 | 0.926 | 1.000 | -0.09 | 0.925 | 0.983 | 1.000 |
| | Left Superior Temporal | 0.49 | 0.626 | 0.977 | 1.000 | 0.54 | 0.594 | 0.896 | 1.000 | 1.45 | 0.151 | 0.896 | 1.000 | 1.40 | 0.166 | 0.855 | 1.000 |
| | Left Supramarginal | -0.50 | 0.620 | 0.977 | 1.000 | -0.64 | 0.524 | 0.896 | 1.000 | -1.13 | 0.263 | 0.926 | 1.000 | -0.57 | 0.573 | 0.973 | 1.000 |
| | Left Frontal Pole | -0.10 | 0.921 | 0.989 | 1.000 | -0.27 | 0.790 | 0.937 | 1.000 | -0.21 | 0.833 | 0.971 | 1.000 | -0.23 | 0.817 | 0.973 | 1.000 |
| | Left Temporal Pole | 0.54 | 0.591 | 0.977 | 1.000 | 0.57 | 0.569 | 0.896 | 1.000 | 0.47 | 0.637 | 0.926 | 1.000 | 0.28 | 0.782 | 0.973 | 1.000 |
| | Left Transverse Temporal | -1.50 | 0.137 | 0.812 | 1.000 | -1.46 | 0.148 | 0.896 | 1.000 | -0.79 | 0.434 | 0.926 | 1.000 | -0.02 | 0.984 | 0.993 | 1.000 |
| | Left Insula | 1.59 | 0.115 | 0.784 | 1.000 | 1.50 | 0.136 | 0.896 | 1.000 | 0.82 | 0.415 | 0.926 | 1.000 | 0.86 | 0.392 | 0.973 | 1.000 |
| | Right Caudal Anterior Cingulate | 0.24 | 0.810 | 0.989 | 1.000 | 0.55 | 0.586 | 0.896 | 1.000 | 0.87 | 0.388 | 0.926 | 1.000 | 1.21 | 0.232 | 0.924 | 1.000 |
| | Right Caudal Middle Frontal | -0.15 | 0.881 | 0.989 | 1.000 | -0.18 | 0.855 | 0.937 | 1.000 | 0.33 | 0.741 | 0.961 | 1.000 | -0.51 | 0.611 | 0.973 | 1.000 |
| | Right Cuneus | -0.21 | 0.832 | 0.989 | 1.000 | -0.27 | 0.787 | 0.937 | 1.000 | -1.21 | 0.230 | 0.926 | 1.000 | -0.69 | 0.494 | 0.973 | 1.000 |
| | Right Entorhinal | -1.26 | 0.211 | 0.907 | 1.000 | -1.38 | 0.172 | 0.896 | 1.000 | -2.17 | 0.032 | 0.416 | 1.000 | -1.46 | 0.147 | 0.828 | 1.000 |
| | Right Fusiform | -0.44 | 0.660 | 0.986 | 1.000 | -0.55 | 0.584 | 0.896 | 1.000 | -1.17 | 0.245 | 0.926 | 1.000 | -1.27 | 0.207 | 0.924 | 1.000 |
| | Right Inferior Parietal | 0.78 | 0.439 | 0.907 | 1.000 | 0.83 | 0.411 | 0.896 | 1.000 | 1.22 | 0.225 | 0.926 | 1.000 | 2.18 | 0.032 | 0.599 | 1.000 |
| | Right Inferior Temporal | -0.18 | 0.861 | 0.989 | 1.000 | 0.02 | 0.988 | 0.992 | 1.000 | -0.01 | 0.994 | 0.996 | 1.000 | -0.10 | 0.921 | 0.983 | 1.000 |
| | Right Isthmus Cingulate | -0.12 | 0.902 | 0.989 | 1.000 | -0.15 | 0.880 | 0.947 | 1.000 | -0.40 | 0.690 | 0.931 | 1.000 | 0.19 | 0.849 | 0.973 | 1.000 |
| | Right Lateral Occipital | -0.28 | 0.779 | 0.989 | 1.000 | -0.21 | 0.831 | 0.937 | 1.000 | -0.40 | 0.689 | 0.931 | 1.000 | 0.52 | 0.604 | 0.973 | 1.000 |
| | Right Lateral Orbitofrontal | -0.12 | 0.903 | 0.989 | 1.000 | -0.19 | 0.853 | 0.937 | 1.000 | 0.91 | 0.367 | 0.926 | 1.000 | 1.11 | 0.271 | 0.950 | 1.000 |
| | Right Lingual | -1.03 | 0.305 | 0.907 | 1.000 | -0.78 | 0.439 | 0.896 | 1.000 | -1.15 | 0.254 | 0.926 | 1.000 | -0.83 | 0.409 | 0.973 | 1.000 |
| | Right Medial Orbitofrontal | -0.61 | 0.544 | 0.940 | 1.000 | -0.56 | 0.575 | 0.896 | 1.000 | 0.42 | 0.674 | 0.926 | 1.000 | -0.07 | 0.941 | 0.990 | 1.000 |
| | Right Middle Temporal | -1.66 | 0.100 | 0.768 | 1.000 | -1.31 | 0.195 | 0.896 | 1.000 | -0.98 | 0.331 | 0.926 | 1.000 | -1.26 | 0.210 | 0.924 | 1.000 |
| | Right Parahippocampal | 1.05 | 0.295 | 0.907 | 1.000 | 1.05 | 0.298 | 0.896 | 1.000 | 0.48 | 0.631 | 0.926 | 1.000 | 0.63 | 0.528 | 0.973 | 1.000 |
| | Right Paracentral | -0.54 | 0.594 | 0.977 | 1.000 | -0.52 | 0.607 | 0.907 | 1.000 | -0.01 | 0.992 | 0.996 | 1.000 | 0.11 | 0.912 | 0.982 | 1.000 |
| | Right Pars Opercularis | 0.88 | 0.380 | 0.907 | 1.000 | 0.92 | 0.359 | 0.896 | 1.000 | 0.74 | 0.464 | 0.926 | 1.000 | 1.04 | 0.303 | 0.950 | 1.000 |
| | Right Pars Orbitalis | -3.27 | 0.002 | 0.084 | 0.338 | -3.56 | 0.001 | 0.084 | 0.169 | -2.80 | 0.006 | 0.292 | 1.000 | -1.40 | 0.164 | 0.855 | 1.000 |
| | Right Pars Triangularis | 1.48 | 0.144 | 0.812 | 1.000 | 1.50 | 0.136 | 0.896 | 1.000 | 2.38 | 0.019 | 0.292 | 1.000 | 2.23 | 0.029 | 0.599 | 1.000 |
| | Right Pericalcarine | -0.70 | 0.484 | 0.907 | 1.000 | -0.63 | 0.528 | 0.896 | 1.000 | -0.94 | 0.352 | 0.926 | 1.000 | -1.21 | 0.229 | 0.924 | 1.000 |
| | Right Postcentral | -1.10 | 0.273 | 0.907 | 1.000 | -1.00 | 0.319 | 0.896 | 1.000 | -0.50 | 0.615 | 0.926 | 1.000 | -0.35 | 0.730 | 0.973 | 1.000 |
| | Right Posterior Cingulate | -1.30 | 0.198 | 0.907 | 1.000 | -1.19 | 0.238 | 0.896 | 1.000 | -0.52 | 0.608 | 0.926 | 1.000 | -0.11 | 0.909 | 0.982 | 1.000 |
| | Right Precentral | -2.31 | 0.023 | 0.399 | 1.000 | -2.41 | 0.018 | 0.338 | 1.000 | -2.38 | 0.019 | 0.292 | 1.000 | -1.81 | 0.074 | 0.695 | 1.000 |
| | Right Precuneus | -0.23 | 0.817 | 0.989 | 1.000 | -0.28 | 0.780 | 0.937 | 1.000 | -0.26 | 0.794 | 0.961 | 1.000 | 0.26 | 0.800 | 0.973 | 1.000 |
| | Right Rostral Anterior Cingulate | -0.33 | 0.740 | 0.989 | 1.000 | -0.12 | 0.903 | 0.957 | 1.000 | 0.33 | 0.745 | 0.961 | 1.000 | 0.54 | 0.588 | 0.973 | 1.000 |
| | Right Rostral Middle Frontal | 1.94 | 0.056 | 0.631 | 1.000 | 1.95 | 0.055 | 0.581 | 1.000 | 2.00 | 0.049 | 0.499 | 1.000 | 1.94 | 0.056 | 0.665 | 1.000 |
| | Right Superior Frontal | -0.76 | 0.452 | 0.907 | 1.000 | -0.93 | 0.355 | 0.896 | 1.000 | -0.22 | 0.830 | 0.971 | 1.000 | -0.49 | 0.625 | 0.973 | 1.000 |
| | Right Superior Parietal | 0.84 | 0.404 | 0.907 | 1.000 | 0.73 | 0.466 | 0.896 | 1.000 | -0.01 | 0.990 | 0.996 | 1.000 | 0.24 | 0.808 | 0.973 | 1.000 |
| | Right Superior Temporal | -0.07 | 0.944 | 0.989 | 1.000 | 0.02 | 0.982 | 0.992 | 1.000 | 0.51 | 0.611 | 0.926 | 1.000 | 1.11 | 0.272 | 0.950 | 1.000 |
| | Right Supramarginal | 0.84 | 0.404 | 0.907 | 1.000 | 0.76 | 0.448 | 0.896 | 1.000 | 1.10 | 0.273 | 0.926 | 1.000 | 0.98 | 0.330 | 0.950 | 1.000 |
| | Right Frontal Pole | 0.31 | 0.756 | 0.989 | 1.000 | 0.26 | 0.792 | 0.937 | 1.000 | 0.01 | 0.996 | 0.996 | 1.000 | 0.21 | 0.835 | 0.973 | 1.000 |
| | Right Temporal Pole | 0.12 | 0.907 | 0.989 | 1.000 | 0.37 | 0.715 | 0.937 | 1.000 | 0.46 | 0.650 | 0.926 | 1.000 | 0.36 | 0.723 | 0.973 | 1.000 |
| | Right Transverse Temporal | 0.30 | 0.762 | 0.989 | 1.000 | 0.25 | 0.805 | 0.937 | 1.000 | -0.70 | 0.485 | 0.926 | 1.000 | -0.34 | 0.732 | 0.973 | 1.000 |
| | Right Insula | 0.01 | 0.991 | 0.996 | 1.000 | -0.19 | 0.851 | 0.937 | 1.000 | 0.71 | 0.482 | 0.926 | 1.000 | 0.16 | 0.875 | 0.973 | 1.000 |

| | | Significance HA as predictor in the model | | | | | | | | | | | | | | | |
|---|---|---|---|---|---|---|---|---|---|---|---|---|---|---|---|---|---|
| | | M1 | | | | M2 | | | | M3 | | | | M4 | | | |
| | | t | punc | pFDR | pBonf | t | punc | pFDR | pBonf | t | punc | pFDR | pBonf | t | punc | pFDR | pBonf |
| Atlas: Desikan-Killiany-Tourville | Left Caudal Anterior Cingulate | -0.86 | 0.395 | 0.907 | 1.000 | -0.72 | 0.471 | 0.896 | 1.000 | -0.19 | 0.850 | 0.973 | 1.000 | -0.04 | 0.967 | 0.990 | 1.000 |
| | Left Caudal Middle Frontal | -0.10 | 0.920 | 0.989 | 1.000 | -0.25 | 0.802 | 0.937 | 1.000 | 0.67 | 0.503 | 0.926 | 1.000 | 0.14 | 0.888 | 0.973 | 1.000 |
| | Left Cuneus | -0.81 | 0.936 | 0.989 | 1.000 | -0.19 | 0.850 | 0.937 | 1.000 | -0.96 | 0.339 | 0.926 | 1.000 | -0.96 | 0.339 | 0.950 | 1.000 |
| | Left Entorhinal | 0.67 | 0.507 | 0.907 | 1.000 | 0.62 | 0.535 | 0.896 | 1.000 | 0.39 | 0.697 | 0.931 | 1.000 | 1.51 | 0.134 | 0.816 | 1.000 |
| | Left Fusiform | 1.31 | 0.193 | 0.907 | 1.000 | 1.44 | 0.152 | 0.896 | 1.000 | 0.84 | 0.405 | 0.926 | 1.000 | 0.62 | 0.537 | 0.973 | 1.000 |
| | Left Inferior Parietal | 0.06 | 0.953 | 0.989 | 1.000 | 0.27 | 0.790 | 0.937 | 1.000 | -0.01 | 0.996 | 0.996 | 1.000 | 0.41 | 0.683 | 0.973 | 1.000 |
| | Left Inferior Temporal | 0.31 | 0.761 | 0.989 | 1.000 | 0.49 | 0.623 | 0.907 | 1.000 | 0.30 | 0.768 | 0.961 | 1.000 | 0.52 | 0.607 | 0.973 | 1.000 |
| | Left Isthmus Cingulate | 0.33 | 0.743 | 0.989 | 1.000 | 0.37 | 0.709 | 0.937 | 1.000 | 0.17 | 0.869 | 0.973 | 1.000 | 0.83 | 0.411 | 0.973 | 1.000 |
| | Left Lateral Occipital | -1.09 | 0.281 | 0.907 | 1.000 | -0.83 | 0.411 | 0.896 | 1.000 | -1.33 | 0.186 | 0.926 | 1.000 | -0.57 | 0.569 | 0.973 | 1.000 |
| | Left Lateral Orbitofrontal | -0.25 | 0.803 | 0.989 | 1.000 | -0.35 | 0.728 | 0.937 | 1.000 | 0.69 | 0.491 | 0.926 | 1.000 | 0.46 | 0.644 | 0.973 | 1.000 |
| | Left Lingual | -0.66 | 0.511 | 0.907 | 1.000 | -0.60 | 0.551 | 0.896 | 1.000 | -0.47 | 0.641 | 0.926 | 1.000 | 0.23 | 0.819 | 0.973 | 1.000 |
| | Left Medial Orbitofrontal | 0.01 | 0.996 | 0.996 | 1.000 | 0.24 | 0.813 | 0.937 | 1.000 | 0.87 | 0.386 | 0.926 | 1.000 | -0.25 | 0.806 | 0.973 | 1.000 |
| | Left Middle Temporal | 0.45 | 0.652 | 0.984 | 1.000 | 0.67 | 0.508 | 0.896 | 1.000 | 0.05 | 0.957 | 0.996 | 1.000 | -0.24 | 0.814 | 0.973 | 1.000 |
| | Left Parahippocampal | -0.87 | 0.388 | 0.907 | 1.000 | -0.74 | 0.460 | 0.896 | 1.000 | 0.64 | 0.527 | 0.926 | 1.000 | 0.95 | 0.343 | 0.950 | 1.000 |
| | Left Paracentral | -2.27 | 0.026 | 0.399 | 1.000 | -2.33 | 0.022 | 0.372 | 1.000 | -1.48 | 0.141 | 0.896 | 1.000 | -1.64 | 0.105 | 0.739 | 1.000 |
| | Left Pars Opercularis | 0.84 | 0.401 | 0.907 | 1.000 | 0.87 | 0.388 | 0.896 | 1.000 | 0.49 | 0.629 | 0.926 | 1.000 | 1.29 | 0.202 | 0.924 | 1.000 |
| | Left Pars Orbitalis | 0.08 | 0.934 | 0.989 | 1.000 | -0.04 | 0.970 | 0.992 | 1.000 | -0.09 | 0.929 | 0.981 | 1.000 | -0.28 | 0.777 | 0.973 | 1.000 |
| | Left Pars Triangularis | 2.72 | 0.008 | 0.225 | 1.000 | 2.50 | 0.014 | 0.338 | 1.000 | 1.67 | 0.099 | 0.727 | 1.000 | 1.73 | 0.088 | 0.739 | 1.000 |
| | Left Pericalcarine | 0.14 | 0.891 | 0.989 | 1.000 | 0.12 | 0.906 | 0.957 | 1.000 | 0.11 | 0.913 | 0.973 | 1.000 | 0.26 | 0.796 | 0.973 | 1.000 |
| | Left Postcentral | -1.23 | 0.223 | 0.907 | 1.000 | -1.04 | 0.302 | 0.896 | 1.000 | -0.69 | 0.490 | 0.926 | 1.000 | -0.48 | 0.634 | 0.973 | 1.000 |
| | Left Posterior Cingulate | -1.24 | 0.218 | 0.907 | 1.000 | -0.99 | 0.326 | 0.896 | 1.000 | -0.76 | 0.451 | 0.926 | 1.000 | -0.04 | 0.965 | 0.990 | 1.000 |
| | Left Precentral | -0.87 | 0.387 | 0.907 | 1.000 | -0.84 | 0.401 | 0.896 | 1.000 | -0.96 | 0.339 | 0.926 | 1.000 | -0.71 | 0.479 | 0.973 | 1.000 |
| | Left Precuneus | 0.70 | 0.488 | 0.907 | 1.000 | 0.54 | 0.589 | 0.896 | 1.000 | 0.14 | 0.888 | 0.973 | 1.000 | 0.55 | 0.582 | 0.973 | 1.000 |
| | Left Rostral Anterior Cingulate | -1.47 | 0.145 | 0.812 | 1.000 | -1.28 | 0.205 | 0.896 | 1.000 | -1.92 | 0.058 | 0.499 | 1.000 | -1.72 | 0.089 | 0.739 | 1.000 |
| | Left Rostral Middle Frontal | 2.59 | 0.011 | 0.266 | 1.000 | 2.50 | 0.014 | 0.338 | 1.000 | 2.52 | 0.013 | 0.292 | 1.000 | 2.61 | 0.011 | 0.599 | 1.000 |
| | Left Superior Frontal | -0.44 | 0.665 | 0.986 | 1.000 | -0.73 | 0.469 | 0.896 | 1.000 | 1.14 | 0.256 | 0.926 | 1.000 | 0.98 | 0.328 | 0.950 | 1.000 |
| | Left Superior Parietal | 0.66 | 0.509 | 0.907 | 1.000 | 0.64 | 0.524 | 0.896 | 1.000 | -0.28 | 0.777 | 0.961 | 1.000 | 0.14 | 0.889 | 0.973 | 1.000 |
| | Left Superior Temporal | 0.71 | 0.481 | 0.907 | 1.000 | 0.75 | 0.458 | 0.896 | 1.000 | 1.53 | 0.129 | 0.872 | 1.000 | 1.54 | 0.127 | 0.816 | 1.000 |
| | Left Supramarginal | -0.43 | 0.672 | 0.988 | 1.000 | -0.57 | 0.570 | 0.896 | 1.000 | -1.00 | 0.321 | 0.926 | 1.000 | -0.36 | 0.722 | 0.973 | 1.000 |
| | Left Transverse Temporal | -1.23 | 0.221 | 0.907 | 1.000 | -1.20 | 0.234 | 0.896 | 1.000 | -0.75 | 0.456 | 0.926 | 1.000 | -0.06 | 0.952 | 0.990 | 1.000 |
| | Left Insula | -0.48 | 0.630 | 0.977 | 1.000 | -0.45 | 0.651 | 0.909 | 1.000 | -0.51 | 0.609 | 0.926 | 1.000 | 0.18 | 0.855 | 0.973 | 1.000 |
| | Right Caudal Anterior Cingulate | 0.35 | 0.725 | 0.989 | 1.000 | 0.65 | 0.517 | 0.896 | 1.000 | 1.02 | 0.309 | 0.926 | 1.000 | 1.38 | 0.172 | 0.855 | 1.000 |
| | Right Caudal Middle Frontal | -0.14 | 0.892 | 0.989 | 1.000 | -0.16 | 0.874 | 0.947 | 1.000 | 0.29 | 0.772 | 0.961 | 1.000 | -0.53 | 0.597 | 0.973 | 1.000 |
| | Right Cuneus | -0.58 | 0.565 | 0.964 | 1.000 | -0.66 | 0.511 | 0.896 | 1.000 | -1.68 | 0.096 | 0.727 | 1.000 | -1.20 | 0.234 | 0.924 | 1.000 |
| | Right Entorhinal | -1.76 | 0.082 | 0.701 | 1.000 | -1.86 | 0.067 | 0.629 | 1.000 | -2.66 | 0.009 | 0.292 | 1.000 | -1.65 | 0.103 | 0.739 | 1.000 |
| | Right Fusiform | -0.08 | 0.940 | 0.989 | 1.000 | -0.18 | 0.859 | 0.937 | 1.000 | -0.77 | 0.446 | 0.926 | 1.000 | -0.97 | 0.333 | 0.950 | 1.000 |
| | Right Inferior Parietal | 0.52 | 0.607 | 0.977 | 1.000 | 0.58 | 0.561 | 0.896 | 1.000 | 0.95 | 0.347 | 0.926 | 1.000 | 2.11 | 0.038 | 0.599 | 1.000 |
| | Right Inferior Temporal | -0.52 | 0.604 | 0.977 | 1.000 | -0.34 | 0.737 | 0.937 | 1.000 | -0.43 | 0.665 | 0.926 | 1.000 | -0.50 | 0.615 | 0.973 | 1.000 |
| | Right Isthmus Cingulate | 0.46 | 0.649 | 0.984 | 1.000 | 0.38 | 0.704 | 0.937 | 1.000 | 0.12 | 0.904 | 0.973 | 1.000 | 1.08 | 0.283 | 0.950 | 1.000 |
| | Right Lateral Occipital | -0.05 | 0.960 | 0.989 | 1.000 | -0.01 | 0.992 | 0.992 | 1.000 | -0.29 | 0.772 | 0.961 | 1.000 | 0.55 | 0.585 | 0.973 | 1.000 |
| | Right Lateral Orbitofrontal | -0.35 | 0.726 | 0.989 | 1.000 | -0.41 | 0.684 | 0.937 | 1.000 | 0.72 | 0.471 | 0.926 | 1.000 | 0.76 | 0.449 | 0.973 | 1.000 |
| | Right Lingual | -1.03 | 0.307 | 0.907 | 1.000 | -0.77 | 0.445 | 0.896 | 1.000 | -1.17 | 0.247 | 0.926 | 1.000 | -0.80 | 0.426 | 0.973 | 1.000 |
| | Right Medial Orbitofrontal | -1.11 | 0.270 | 0.907 | 1.000 | -1.11 | 0.269 | 0.896 | 1.000 | 0.43 | 0.671 | 0.926 | 1.000 | -0.03 | 0.976 | 0.993 | 1.000 |
| | Right Middle Temporal | -1.31 | 0.194 | 0.907 | 1.000 | -0.96 | 0.341 | 0.896 | 1.000 | -0.58 | 0.567 | 0.926 | 1.000 | -0.93 | 0.354 | 0.963 | 1.000 |
| | Right Parahippocampal | 0.80 | 0.428 | 0.907 | 1.000 | 0.79 | 0.434 | 0.896 | 1.000 | 0.24 | 0.812 | 0.961 | 1.000 | -0.01 | 0.994 | 0.994 | 1.000 |
| | Right Paracentral | -0.50 | 0.616 | 0.977 | 1.000 | -0.47 | 0.640 | 0.907 | 1.000 | 0.07 | 0.942 | 0.989 | 1.000 | 0.15 | 0.883 | 0.973 | 1.000 |
| | Right Pars Opercularis | 0.67 | 0.506 | 0.907 | 1.000 | 0.74 | 0.463 | 0.896 | 1.000 | 0.44 | 0.663 | 0.926 | 1.000 | 0.54 | 0.593 | 0.973 | 1.000 |
| | Right Pars Orbitalis | -3.27 | 0.002 | 0.084 | 0.338 | -3.55 | 0.001 | 0.084 | 0.169 | -2.94 | 0.004 | 0.292 | 0.676 | -1.96 | 0.054 | 0.665 | 1.000 |
| | Right Pars Triangularis | 1.46 | 0.149 | 0.812 | 1.000 | 1.46 | 0.149 | 0.896 | 1.000 | 2.44 | 0.017 | 0.292 | 1.000 | 2.38 | 0.019 | 0.599 | 1.000 |
| | Right Pericalcarine | -0.73 | 0.465 | 0.907 | 1.000 | -0.65 | 0.520 | 0.896 | 1.000 | -0.97 | 0.335 | 0.926 | 1.000 | -1.25 | 0.214 | 0.924 | 1.000 |
| | Right Postcentral | -1.29 | 0.200 | 0.907 | 1.000 | -1.19 | 0.239 | 0.896 | 1.000 | -0.62 | 0.535 | 0.926 | 1.000 | -0.46 | 0.645 | 0.973 | 1.000 |
| | Right Posterior Cingulate | -1.17 | 0.246 | 0.907 | 1.000 | -1.04 | 0.301 | 0.896 | 1.000 | -0.39 | 0.700 | 0.931 | 1.000 | -0.18 | 0.857 | 0.973 | 1.000 |
| | Right Precentral | -2.28 | 0.025 | 0.399 | 1.000 | -2.40 | 0.018 | 0.338 | 1.000 | -2.40 | 0.018 | 0.292 | 1.000 | -1.89 | 0.062 | 0.665 | 1.000 |
| | Right Precuneus | -0.29 | 0.769 | 0.989 | 1.000 | -0.33 | 0.741 | 0.937 | 1.000 | -0.28 | 0.780 | 0.961 | 1.000 | 0.16 | 0.872 | 0.973 | 1.000 |
| | Right Rostral Anterior Cingulate | 0.08 | 0.938 | 0.989 | 1.000 | 0.32 | 0.751 | 0.937 | 1.000 | 0.78 | 0.440 | 0.926 | 1.000 | 1.01 | 0.314 | 0.950 | 1.000 |
| | Right Rostral Middle Frontal | 2.89 | 0.005 | 0.169 | 0.845 | 2.86 | 0.005 | 0.169 | 0.845 | 2.57 | 0.012 | 0.292 | 1.000 | 2.77 | 0.007 | 0.592 | 1.000 |
| | Right Superior Frontal | -0.89 | 0.375 | 0.907 | 1.000 | -0.99 | 0.325 | 0.896 | 1.000 | -0.43 | 0.669 | 0.926 | 1.000 | -0.67 | 0.508 | 0.973 | 1.000 |
| | Right Superior Parietal | 1.11 | 0.272 | 0.907 | 1.000 | 1.01 | 0.315 | 0.896 | 1.000 | 0.27 | 0.791 | 0.961 | 1.000 | 0.54 | 0.592 | 0.973 | 1.000 |
| | Right Superior Temporal | 0.15 | 0.882 | 0.989 | 1.000 | 0.25 | 0.803 | 0.937 | 1.000 | 0.58 | 0.565 | 0.926 | 1.000 | 0.99 | 0.325 | 0.950 | 1.000 |
| | Right Supramarginal | 0.86 | 0.391 | 0.907 | 1.000 | 0.76 | 0.451 | 0.896 | 1.000 | 1.34 | 0.185 | 0.926 | 1.000 | 1.08 | 0.285 | 0.950 | 1.000 |
| | Right Transverse Temporal | 0.09 | 0.926 | 0.989 | 1.000 | 0.02 | 0.987 | 0.992 | 1.000 | -0.81 | 0.420 | 0.926 | 1.000 | -0.41 | 0.685 | 0.973 | 1.000 |
| | Right Insula | -0.37 | 0.715 | 0.989 | 1.000 | -0.47 | 0.637 | 0.907 | 1.000 | 0.81 | 0.420 | 0.926 | 1.000 | 0.37 | 0.713 | 0.973 | 1.000 |
| Atlas: Talairach | Left BA 1 | -1.22 | 0.226 | 0.907 | 1.000 | -1.10 | 0.276 | 0.896 | 1.000 | -0.43 | 0.671 | 0.926 | 1.000 | -0.41 | 0.685 | 0.973 | 1.000 |
| | Left BA 2 | -0.89 | 0.376 | 0.907 | 1.000 | -0.83 | 0.407 | 0.896 | 1.000 | -0.54 | 0.592 | 0.926 | 1.000 | -0.69 | 0.491 | 0.973 | 1.000 |
| | Left BA 3a | -0.17 | 0.865 | 0.989 | 1.000 | 0.06 | 0.955 | 0.984 | 1.000 | -0.71 | 0.480 | 0.926 | 1.000 | -0.66 | 0.512 | 0.973 | 1.000 |
| | Left BA 3b | -1.11 | 0.269 | 0.907 | 1.000 | -0.88 | 0.380 | 0.896 | 1.000 | -0.76 | 0.447 | 0.926 | 1.000 | -0.36 | 0.722 | 0.973 | 1.000 |
| | Left BA 4a | -1.28 | 0.205 | 0.907 | 1.000 | -1.29 | 0.200 | 0.896 | 1.000 | -1.16 | 0.249 | 0.926 | 1.000 | -1.14 | 0.260 | 0.950 | 1.000 |
| | Left BA 4p | -0.71 | 0.478 | 0.907 | 1.000 | -0.67 | 0.506 | 0.896 | 1.000 | -1.43 | 0.157 | 0.896 | 1.000 | -0.87 | 0.386 | 0.973 | 1.000 |
| | Left BA 6 | -1.61 | 0.111 | 0.784 | 1.000 | -1.72 | 0.089 | 0.752 | 1.000 | -0.54 | 0.593 | 0.926 | 1.000 | -0.78 | 0.435 | 0.973 | 1.000 |
| | Left BA 44 | 0.97 | 0.337 | 0.907 | 1.000 | 0.94 | 0.349 | 0.896 | 1.000 | 0.76 | 0.450 | 0.926 | 1.000 | 0.92 | 0.359 | 0.963 | 1.000 |
| | Left BA 45 | 3.22 | 0.002 | 0.084 | 0.338 | 3.03 | 0.003 | 0.169 | 0.507 | 1.96 | 0.053 | 0.499 | 1.000 | 2.31 | 0.023 | 0.599 | 1.000 |
| | Left V1 | 0.26 | 0.796 | 0.989 | 1.000 | 0.18 | 0.856 | 0.937 | 1.000 | 0.55 | 0.583 | 0.926 | 1.000 | 0.78 | 0.439 | 0.973 | 1.000 |
| | Left V2 | -0.87 | 0.389 | 0.907 | 1.000 | -0.77 | 0.442 | 0.896 | 1.000 | -1.18 | 0.241 | 0.926 | 1.000 | -0.47 | 0.639 | 0.973 | 1.000 |
| | Left MT | 0.87 | 0.388 | 0.907 | 1.000 | 1.12 | 0.264 | 0.896 | 1.000 | 0.14 | 0.890 | 0.973 | 1.000 | 0.82 | 0.417 | 0.973 | 1.000 |
| | Left Perirhinal | -0.73 | 0.465 | 0.907 | 1.000 | -0.73 | 0.468 | 0.896 | 1.000 | -0.62 | 0.539 | 0.926 | 1.000 | 0.14 | 0.892 | 0.973 | 1.000 |
| | Right BA 1 | -0.82 | 0.412 | 0.907 | 1.000 | -0.75 | 0.453 | 0.896 | 1.000 | -0.17 | 0.869 | 0.973 | 1.000 | 0.24 | 0.815 | 0.973 | 1.000 |
| | Right BA 2 | 0.05 | 0.959 | 0.989 | 1.000 | 0.10 | 0.924 | 0.964 | 1.000 | 0.45 | 0.651 | 0.926 | 1.000 | 0.18 | 0.859 | 0.973 | 1.000 |
| | Right BA 3a | 0.46 | 0.650 | 0.984 | 1.000 | 0.43 | 0.666 | 0.923 | 1.000 | -0.02 | 0.987 | 0.996 | 1.000 | 0.28 | 0.783 | 0.973 | 1.000 |
| | Right BA 3b | -1.02 | 0.313 | 0.907 | 1.000 | -0.90 | 0.371 | 0.896 | 1.000 | -0.03 | 0.976 | 0.996 | 1.000 | 0.23 | 0.822 | 0.973 | 1.000 |
| | Right BA 4a | -1.85 | 0.067 | 0.666 | 1.000 | -1.99 | 0.049 | 0.581 | 1.000 | -2.11 | 0.038 | 0.459 | 1.000 | -1.38 | 0.171 | 0.855 | 1.000 |
| | Right BA 4p | -0.79 | 0.434 | 0.907 | 1.000 | -1.00 | 0.319 | 0.896 | 1.000 | -1.43 | 0.156 | 0.896 | 1.000 | -1.02 | 0.313 | 0.950 | 1.000 |
| | Right BA 6 | -1.93 | 0.056 | 0.631 | 1.000 | -2.03 | 0.046 | 0.581 | 1.000 | -0.82 | 0.413 | 0.926 | 1.000 | -0.46 | 0.644 | 0.973 | 1.000 |
| | Right BA 44 | -0.29 | 0.770 | 0.989 | 1.000 | -0.23 | 0.822 | 0.937 | 1.000 | -0.11 | 0.915 | 0.973 | 1.000 | -0.14 | 0.887 | 0.973 | 1.000 |
| | Right BA 45 | 1.25 | 0.214 | 0.907 | 1.000 | 1.21 | 0.228 | 0.896 | 1.000 | 1.91 | 0.059 | 0.499 | 1.000 | 2.10 | 0.039 | 0.599 | 1.000 |
| | Right V1 | -0.81 | 0.421 | 0.907 | 1.000 | -0.69 | 0.492 | 0.896 | 1.000 | -1.06 | 0.292 | 0.926 | 1.000 | -0.84 | 0.402 | 0.973 | 1.000 |
| | Right V2 | -0.77 | 0.442 | 0.907 | 1.000 | -0.63 | 0.529 | 0.896 | 1.000 | -1.14 | 0.258 | 0.926 | 1.000 | -0.02 | 0.987 | 0.993 | 1.000 |
| | Right MT | 1.75 | 0.083 | 0.701 | 1.000 | 1.68 | 0.096 | 0.773 | 1.000 | 1.73 | 0.087 | 0.700 | 1.000 | 1.68 | 0.097 | 0.739 | 1.000 |
| | Right Perirhinal | -1.56 | 0.123 | 0.795 | 1.000 | -1.78 | 0.079 | 0.703 | 1.000 | -2.00 | 0.049 | 0.499 | 1.000 | -1.55 | 0.124 | 0.816 | 1.000 |
