# Supplementary material for: The Heterogeneity in Retrieved Relations between the Personality Trait ‘Harm Avoidance’ and Gray Matter Volumes Due to Variations in the VBM and ROI Labeling Processing Settings
Source: PLoS One. 2016 Apr 20;11(4):e0153865. doi: 10.1371/journal.pone.0153865 (PMC4838261; doi:10.1371/journal.pone.0153865)
Supplement: S4 File — None of the correlations survived multiple comparisons correction. (PDF) [file pone.0153865.s004.pdf]

	Significance HA as predictor in the model															
	M1				M2				M3				M4			
	t	p(unc)	p(FDR)	p(FWE)	t	p(unc)	p(FDR)	p(FWE)	t	p(unc)	p(FDR)	p(FWE)	t	p(unc)	p(FDR)	p(FWE)
Precentral L	1,15	0,251	0,993	1,000	0,96	0,339	0,989	1,000	0,59	0,555	0,970	1,000	0,91	0,364	0,997	1,000
Precentral R	0,79	0,431	0,993	1,000	0,75	0,455	0,989	1,000	1,08	0,281	0,970	1,000	0,87	0,389	0,997	1,000
Frontal Sup L	0,37	0,709	0,993	1,000	0,31	0,757	0,989	1,000	0,83	0,410	0,970	1,000	0,69	0,495	0,997	1,000
Frontal Sup R	0,03	0,980	0,993	1,000	0,06	0,957	0,989	1,000	0,74	0,459	0,970	1,000	0,55	0,582	0,997	1,000
Frontal Sup Orb L	0,61	0,543	0,993	1,000	0,44	0,659	0,989	1,000	1,42	0,159	0,970	1,000	1,21	0,229	0,997	1,000
Frontal Sup Orb R	-0,07	0,945	0,993	1,000	-0,05	0,963	0,989	1,000	0,81	0,420	0,970	1,000	0,28	0,781	0,997	1,000
Frontal Mid L	0,69	0,492	0,993	1,000	0,69	0,493	0,989	1,000	1,11	0,270	0,970	1,000	1,08	0,284	0,997	1,000
Frontal Mid R	0,46	0,650	0,993	1,000	0,49	0,626	0,989	1,000	0,86	0,391	0,970	1,000	0,56	0,578	0,997	1,000
Frontal Mid Orb L	0,14	0,893	0,993	1,000	0,09	0,930	0,989	1,000	0,76	0,449	0,970	1,000	0,61	0,545	0,997	1,000
Frontal Mid Orb R	-0,78	0,435	0,993	1,000	-0,67	0,504	0,989	1,000	-0,15	0,885	0,970	1,000	-0,86	0,393	0,997	1,000
Frontal Inf Oper L	-0,54	0,592	0,993	1,000	-0,60	0,549	0,989	1,000	0,46	0,651	0,970	1,000	0,29	0,774	0,997	1,000
Frontal Inf Oper R	-0,01	0,993	0,993	1,000	0,04	0,971	0,989	1,000	0,42	0,675	0,970	1,000	-0,27	0,790	0,997	1,000
Frontal Inf Tri L	0,32	0,748	0,993	1,000	0,40	0,688	0,989	1,000	0,83	0,407	0,970	1,000	1,06	0,292	0,997	1,000
Frontal Inf Tri R	-0,21	0,837	0,993	1,000	-0,17	0,869	0,989	1,000	0,26	0,797	0,970	1,000	-0,36	0,720	0,997	1,000
Frontal Inf Orb L	-0,30	0,769	0,993	1,000	-0,31	0,756	0,989	1,000	0,30	0,764	0,970	1,000	0,11	0,910	0,997	1,000
Frontal Inf Orb R	-0,24	0,812	0,993	1,000	-0,14	0,886	0,989	1,000	0,45	0,657	0,970	1,000	-0,05	0,957	0,997	1,000
Rolandic Oper L	0,35	0,729	0,993	1,000	0,31	0,755	0,989	1,000	0,15	0,885	0,970	1,000	0,42	0,678	0,997	1,000
Rolandic Oper R	-1,09	0,281	0,993	1,000	-0,91	0,366	0,989	1,000	-0,19	0,849	0,970	1,000	-0,73	0,465	0,997	1,000
Supp Motor Area L	-0,34	0,732	0,993	1,000	-0,44	0,663	0,989	1,000	0,56	0,576	0,970	1,000	0,09	0,931	0,997	1,000
Supp Motor Area R	0,07	0,941	0,993	1,000	-0,03	0,980	0,989	1,000	0,82	0,415	0,970	1,000	0,55	0,581	0,997	1,000
Olfactory L	0,07	0,948	0,993	1,000	0,03	0,976	0,989	1,000	0,11	0,909	0,970	1,000	0,02	0,981	0,997	1,000
Olfactory R	0,47	0,638	0,993	1,000	0,38	0,705	0,989	1,000	0,71	0,479	0,970	1,000	0,71	0,481	0,997	1,000
Frontal Sup Medial L	-0,92	0,359	0,993	1,000	-0,95	0,344	0,989	1,000	0,38	0,704	0,970	1,000	-0,18	0,857	0,997	1,000
Frontal Sup Medial R	0,41	0,686	0,993	1,000	0,45	0,655	0,989	1,000	1,09	0,279	0,970	1,000	0,94	0,348	0,997	1,000
Frontal Med Orb L	0,56	0,578	0,993	1,000	0,50	0,616	0,989	1,000	1,77	0,081	0,970	1,000	1,52	0,134	0,997	1,000
Frontal Med Orb R	-0,39	0,696	0,993	1,000	-0,44	0,662	0,989	1,000	0,21	0,832	0,970	1,000	-0,06	0,950	0,997	1,000
Rectus L	-0,39	0,697	0,993	1,000	-0,29	0,770	0,989	1,000	0,61	0,544	0,970	1,000	-0,01	0,996	0,997	1,000
Rectus R	-0,36	0,722	0,993	1,000	-0,19	0,854	0,989	1,000	0,64	0,526	0,970	1,000	0,34	0,733	0,997	1,000
Insula L	-1,06	0,294	0,993	1,000	-1,07	0,287	0,989	1,000	-0,93	0,353	0,970	1,000	-0,33	0,744	0,997	1,000
Insula R	-1,14	0,259	0,993	1,000	-1,04	0,302	0,989	1,000	-0,40	0,692	0,970	1,000	-0,62	0,537	0,997	1,000
Cingulum Ant L	0,68	0,500	0,993	1,000	0,63	0,531	0,989	1,000	1,19	0,239	0,970	1,000	0,88	0,381	0,997	1,000
Cingulum Ant R	1,70	0,092	0,993	1,000	1,57	0,119	0,989	1,000	1,63	0,107	0,970	1,000	1,42	0,159	0,997	1,000
Cingulum Mid L	0,22	0,828	0,993	1,000	0,30	0,765	0,989	1,000	0,50	0,617	0,970	1,000	0,14	0,888	0,997	1,000
Cingulum Mid R	0,20	0,843	0,993	1,000	0,25	0,803	0,989	1,000	0,54	0,592	0,970	1,000	0,26	0,794	0,997	1,000
Cingulum Post L	-0,54	0,592	0,993	1,000	-0,33	0,742	0,989	1,000	0,17	0,863	0,970	1,000	-0,34	0,736	0,997	1,000
Cingulum Post R	-0,38	0,708	0,993	1,000	-0,19	0,850	0,989	1,000	0,18	0,856	0,970	1,000	0,00	0,997	0,997	1,000
Hippocampus L	-0,34	0,735	0,993	1,000	-0,42	0,679	0,989	1,000	-0,55	0,587	0,970	1,000	-0,27	0,788	0,997	1,000
Hippocampus R	-1,77	0,810	0,993	1,000	-1,67	0,098	0,989	1,000	-1,00	0,322	0,970	1,000	-1,54	0,127	0,997	1,000
ParaHippocampal L	-1,78	0,078	0,993	1,000	-1,63	0,107	0,989	1,000	-1,19	0,236	0,970	1,000	-0,62	0,538	0,997	1,000
ParaHippocampal R	-1,07	0,287	0,993	1,000	-0,95	0,343	0,989	1,000	-0,76	0,447	0,970	1,000	-1,15	0,254	0,997	1,000
Amygdala L	-0,41	0,681	0,993	1,000	-0,36	0,723	0,989	1,000	-0,20	0,842	0,970	1,000	0,09	0,925	0,997	1,000
Amygdala R	-0,10	0,922	0,993	1,000	-0,12	0,903	0,989	1,000	-0,28	0,779	0,970	1,000	-0,17	0,865	0,997	1,000
Calcarine L	-0,19	0,847	0,993	1,000	-0,08	0,935	0,989	1,000	-0,08	0,935	0,979	1,000	0,24	0,815	0,997	1,000
Calcarine R	1,47	0,145	0,993	1,000	1,42	0,159	0,989	1,000	0,81	0,423	0,970	1,000	1,42	0,160	0,997	1,000
Cuneus L	0,12	0,907	0,993	1,000	0,21	0,835	0,989	1,000	0,17	0,862	0,970	1,000	-0,12	0,906	0,997	1,000
Cuneus R	2,04	0,045	0,993	1,000	1,88	0,064	0,989	1,000	1,37	0,173	0,970	1,000	0,86	0,394	0,997	1,000
Lingual L	-1,30	0,197	0,993	1,000	-1,16	0,249	0,989	1,000	-0,52	0,608	0,970	1,000	-0,92	0,363	0,997	1,000
Lingual R	-0,74	0,464	0,993	1,000	-0,59	0,554	0,989	1,000	-0,20	0,838	0,970	1,000	-1,23	0,222	0,997	1,000
Occipital Sup L	0,69	0,494	0,993	1,000	0,82	0,416	0,989	1,000	0,78	0,435	0,970	1,000	0,35	0,729	0,997	1,000
Occipital Sup R	0,08	0,936	0,993	1,000	0,25	0,806	0,989	1,000	0,77	0,445	0,970	1,000	0,21	0,837	0,997	1,000
Occipital Mid L	0,75	0,453	0,993	1,000	0,84	0,405	0,989	1,000	1,32	0,190	0,970	1,000	1,21	0,228	0,997	1,000
Occipital Mid R	-0,23	0,817	0,993	1,000	-0,05	0,964	0,989	1,000	0,43	0,669	0,970	1,000	-0,12	0,904	0,997	1,000
Occipital Inf L	0,12	0,904	0,993	1,000	0,22	0,826	0,989	1,000	0,34	0,732	0,970	1,000	-0,45	0,652	0,997	1,000
Occipital Inf R	0,17	0,865	0,993	1,000	0,36	0,723	0,989	1,000	1,01	0,313	0,970	1,000	0,03	0,978	0,997	1,000
Fusiform L	0,45	0,656	0,993	1,000	0,43	0,668	0,989	1,000	0,97	0,333	0,970	1,000	0,39	0,694	0,997	1,000
Fusiform R	0,28	0,781	0,993	1,000	0,35	0,731	0,989	1,000	0,53	0,598	0,970	1,000	0,01	0,993	0,997	1,000
Postcentral L	-0,18	0,858	0,993	1,000	0,00	0,997	0,997	1,000	0,01	0,994	0,994	1,000	-0,26	0,796	0,997	1,000
Postcentral R	-0,12	0,908	0,993	1,000	0,11	0,917	0,989	1,000	0,54	0,591	0,970	1,000	-0,01	0,991	0,997	1,000
Parietal Sup L	0,04	0,972	0,993	1,000	0,17	0,864	0,989	1,000	0,52	0,601	0,970	1,000	0,13	0,899	0,997	1,000
Parietal Sup R	-0,28	0,781	0,993	1,000	-0,20	0,844	0,989	1,000	0,11	0,911	0,970	1,000	-0,57	0,572	0,997	1,000
Parietal Inf L	0,85	0,399	0,993	1,000	0,88	0,381	0,989	1,000	1,05	0,297	0,970	1,000	0,70	0,484	0,997	1,000
Parietal Inf R	-0,18	0,861	0,993	1,000	-0,10	0,922	0,989	1,000	0,40	0,689	0,970	1,000	-0,20	0,841	0,997	1,000
SupraMarginal L	-0,52	0,606	0,993	1,000	-0,53	0,599	0,989	1,000	0,13	0,895	0,970	1,000	0,54	0,592	0,997	1,000
SupraMarginal R	-0,03	0,973	0,993	1,000	0,10	0,924	0,989	1,000	0,92	0,358	0,970	1,000	0,24	0,811	0,997	1,000
Angular L	0,91	0,367	0,993	1,000	1,03	0,304	0,989	1,000	1,41	0,162	0,970	1,000	1,16	0,250	0,997	1,000
Angular R	-0,04	0,967	0,993	1,000	0,12	0,905	0,989	1,000	0,81	0,418	0,970	1,000	0,19	0,847	0,997	1,000
Precuneus L	-0,56	0,577	0,993	1,000	-0,41	0,680	0,989	1,000	0,14	0,889	0,970	1,000	-0,52	0,606	0,997	1,000
Precuneus R	-0,85	0,400	0,993	1,000	-0,66	0,510	0,989	1,000	-0,16	0,872	0,970	1,000	-0,55	0,587	0,997	1,000
Paracentral Lobule L	-1,01	0,315	0,993	1,000	-0,88	0,383	0,989	1,000	-0,21	0,837	0,970	1,000	-0,24	0,814	0,997	1,000
Paracentral Lobule R	-0,83	0,412	0,993	1,000	-0,71	0,482	0,989	1,000	0,05	0,959	0,984	1,000	0,09	0,930	0,997	1,000
Caudate L	1,27	0,207	0,993	1,000	1,20	0,232	0,989	1,000	1,29	0,199	0,970	1,000	0,66	0,511	0,997	1,000
Caudate R	-0,16	0,872	0,993	1,000	-0,27	0,788	0,989	1,000	0,01	0,989	0,994	1,000	-0,33	0,742	0,997	1,000
Putamen L	-0,89	0,376	0,993	1,000	-0,98	0,328	0,989	1,000	-1,53	0,131	0,970	1,000	-0,85	0,400	0,997	1,000
Putamen R	0,18	0,862	0,993	1,000	0,05	0,960	0,989	1,000	-0,63	0,528	0,970	1,000	-0,44	0,662	0,997	1,000
Pallidum L	-0,18	0,858	0,993	1,000	-0,30	0,767	0,989	1,000	-0,43	0,670	0,970	1,000	0,25	0,805	0,997	1,000
Pallidum R	0,77	0,446	0,993	1,000	0,59	0,560	0,989	1,000	0,17	0,862	0,970	1,000	0,69	0,495	0,997	1,000
Thalamus L	-0,10	0,918	0,993	1,000	0,03	0,979	0,989	1,000	0,17	0,869	0,970	1,000	-0,26	0,793	0,997	1,000
Thalamus R	-0,78	0,437	0,993	1,000	-0,59	0,554	0,989	1,000	-0,40	0,693	0,970	1,000	-0,54	0,590	0,997	1,000
Heschl L	0,82	0,413	0,993	1,000	0,63	0,530	0,989	1,000	0,07	0,948	0,982	1,000	0,49	0,628	0,997	1,000
Heschl R	2,19	0,031	0,993	1,000	1,92	0,058	0,989	1,000	1,74	0,085	0,970	1,000	2,02	0,046	0,997	1,000
Temporal Sup L	0,60	0,549	0,993	1,000	0,58	0,567	0,989	1,000	0,64	0,522	0,970	1,000	0,99	0,324	0,997	1,000
Temporal Sup R	-0,20	0,845	0,993	1,000	-0,14	0,890	0,989	1,000	0,50	0,617	0,970	1,000	0,01	0,993	0,997	1,000
Temporal Pole Sup L	-1,72	0,089	0,993	1,000	-1,71	0,090	0,989	1,000	-1,72	0,088	0,970	1,000	-1,33	0,189	0,997	1,000
Temporal Pole Sup R	-1,41	0,163	0,993	1,000	-1,29	0,200	0,989	1,000	-1,49	0,139	0,970	1,000	-2,26	0,027	0,997	1,000
Temporal Mid L	0,20	0,842	0,993	1,000	0,24	0,813	0,989	1,000	0,56	0,576	0,970	1,000	0,83	0,408	0,997	1,000
Temporal Mid R	-0,16	0,870	0,993	1,000	-0,05	0,963	0,989	1,000	0,64	0,527	0,970	1,000	0,23	0,820	0,997	1,000
Temporal Pole Mid L	0,36	0,717	0,993	1,000	0,60	0,550	0,989	1,000	0,86	0,392	0,970	1,000	0,36	0,718	0,997	1,000
Temporal Pole Mid R	-0,34	0,734	0,993	1,000	-0,90	0,928	0,989	1,000	-0,15	0,881	0,970	1,000	-0,60	0,549	0,997	1,000
Temporal Inf L	0,37	0,712	0,993	1,000	0,30	0,762	0,989	1,000	0,47	0,638	0,970	1,000	0,28	0,779	0,997	1,000
Temporal Inf R	-0,21	0,832	0,993	1,000	-0,04	0,967	0,989	1,000	0,25	0,806	0,970	1,000	-0,35	0,730	0,997	1,000
Cerebelum Crus1 L	0,01	0,991	0,993	1,000	0,05	0,958	0,989	1,000	0,74	0,459	0,970	1,000	0,57	0,569	0,997	1,000
Cerebelum Crus1 R	-0,28	0,778	0,993	1,000	-0,20	0,840	0,989	1,000	0,36	0,717	0,970	1,000	-0,13	0,898	0,997	1,000
Cerebelum Crus2 L	0,22	0,828	0,993	1,000	0,28	0,781	0,989	1,000	0,84	0,406	0,970	1,000	0,16	0,877	0,997	1,000
Cerebelum Crus2 R	-0,32	0,753	0,993	1,000	-0,21	0,838	0,989	1,000	0,24	0,812	0,970	1,000	-0,73	0,470	0,997	1,000
Cerebelum 3 L	-0,89	0,375	0,993	1,000	-0,76	0,451	0,989	1,000	-0,56	0,578	0,970	1,000	-0,75	0,458	0,997	1,000
Cerebelum 3 R	-1,19	0,236	0,993	1,000	-1,05	0,296	0,989	1,000	-0,64	0,524	0,970	1,000	-1,34	0,185	0,997	1,000
Cerebelum 4-5 L	-0,53	0,595	0,993	1,000	-0,48	0,632	0,989	1,000	-0,37	0,716	0,970	1,000	-0,44	0,660	0,997	1,000

	Significance HA as predictor in the model															
	M1				M2				M3				M4			
	t	p(unc)	p(FDR)	p(FWE)	t	p(unc)	p(FDR)	p(FWE)	t	p(unc)	p(FDR)	p(FWE)	t	p(unc)	p(FDR)	p(FWE)
Cerebelum 4-5 R	-0,28	0,781	0,993	1,000	-0,22	0,824	0,989	1,000	-0,14	0,887	0,970	1,000	-0,31	0,757	0,997	1,000
Cerebelum 6 L	0,17	0,863	0,993	1,000	0,21	0,836	0,989	1,000	0,39	0,701	0,970	1,000	0,64	0,527	0,997	1,000
Cerebelum 6 R	-0,11	0,915	0,993	1,000	-0,07	0,945	0,989	1,000	0,20	0,843	0,970	1,000	0,18	0,858	0,997	1,000
Cerebelum 7b L	-0,04	0,972	0,993	1,000	-0,05	0,961	0,989	1,000	0,31	0,756	0,970	1,000	-0,46	0,645	0,997	1,000
Cerebelum 7 b R	-0,63	0,530	0,993	1,000	-0,53	0,596	0,989	1,000	-0,29	0,775	0,970	1,000	-1,17	0,244	0,997	1,000
Cerebelum 8 L	-0,26	0,798	0,993	1,000	-0,20	0,840	0,989	1,000	0,16	0,875	0,970	1,000	-0,56	0,581	0,997	1,000
Cerebelum 8 R	-0,68	0,498	0,993	1,000	-0,55	0,582	0,989	1,000	-0,14	0,887	0,970	1,000	-1,00	0,319	0,997	1,000
Cerebelum 9 L	-0,38	0,706	0,993	1,000	-0,28	0,780	0,989	1,000	-0,08	0,937	0,979	1,000	-1,09	0,280	0,997	1,000
Cerebelum 9 R	-0,43	0,668	0,993	1,000	-0,31	0,758	0,989	1,000	-0,02	0,981	0,994	1,000	-0,99	0,324	0,997	1,000
Cerebelum 10 L	-1,01	0,316	0,993	1,000	-1,01	0,316	0,989	1,000	-0,91	0,364	0,970	1,000	-1,17	0,244	0,997	1,000
Cerebelum 10 R	-1,79	0,077	0,993	1,000	-1,69	0,095	0,989	1,000	-1,36	0,175	0,970	1,000	-1,92	0,059	0,997	1,000
Vermis 1-2	0,17	0,865	0,993	1,000	0,24	0,814	0,989	1,000	0,50	0,620	0,970	1,000	0,20	0,840	0,997	1,000
Vermis 3	-1,57	0,120	0,993	1,000	-1,33	0,188	0,989	1,000	-1,06	0,294	0,970	1,000	-1,29	0,202	0,997	1,000
Vermis 4-5	-1,38	0,172	0,993	1,000	-1,19	0,236	0,989	1,000	-0,73	0,468	0,970	1,000	-1,10	0,277	0,997	1,000
Vermis 6	-0,01	0,992	0,993	1,000	0,10	0,920	0,989	1,000	0,39	0,700	0,970	1,000	0,01	0,993	0,997	1,000
Vermis 7	0,86	0,390	0,993	1,000	0,91	0,364	0,989	1,000	1,15	0,255	0,970	1,000	0,49	0,625	0,997	1,000
Vermis 8	0,22	0,829	0,993	1,000	0,25	0,804	0,989	1,000	0,37	0,710	0,970	1,000	-0,69	0,494	0,997	1,000
Vermis 9	-0,11	0,912	0,993	1,000	-0,05	0,964	0,989	1,000	0,30	0,769	0,970	1,000	-0,91	0,367	0,997	1,000
Vermis 10	-1,51	0,134	0,993	1,000	-1,32	0,191	0,989	1,000	-0,78	0,437	0,970	1,000	-1,96	0,054	0,997	1,000
